# Epidemiology of interstitial lung diseases and their progressive-fibrosing behaviour in six European countries

**DOI:** 10.1183/23120541.00597-2021

**Published:** 2022-01-24

**Authors:** Ole Hilberg, Anna-Maria Hoffmann-Vold, Vanessa Smith, Demosthenes Bouros, Maritta Kilpeläinen, Julien Guiot, Antonio Morais, Susana Clemente, Zoe Daniil, Despina Papakosta, Havard Fretheim, Sofia Neves, Tiago M. Alfaro, Katerina M. Antoniou, Neus Valveny, Guus Asijee, Stéphane Soulard, Wim Wuyts

**Affiliations:** 1 IRS-centre, Lillebælt Hospital, Vejle, Denmark; 2 Dept of Rheumatology, Oslo University Hospital – Rikshospitalet, Oslo, Norway; 3 Dept of Rheumatology, Ghent University Hospital, Ghent, Belgium; 4 Dept of Pneumonology, Athens Medical Centre, Maroussi, Greece; 5 National and Kapodistrian University of Athens, Athens, Greece; 6 Dept of Pulmonary Diseases, Turku University Hospital and University of Turku, Turku, Finland; 7 Dept of Respiratory Medicine, Liege University Hospital Centre, Liege, Belgium; 8 Pulmonary Dept, Sao Joao University Hospital Centre, Porto, Portugal; 9 Pulmonary Dept, Beatriz Angelo Hospital, Loures, Portugal; 10 Dept of Respiratory Medicine, University Hospital of Larissa, Faculty of Medicine, School of Health Sciences, University of Thessaly, Larissa, Greece; 11 Dept of Respiratory Medicine, Aristotle University of Thessaloniki, George Papanikolaou General Hospital, Thessaloniki, Greece; 12 Pulmonary Dept, Vila Nova de Gaia/Espinho Hospital Centre, Vila Nova de Gaia, Portugal; 13 Pulmonary Dept, Centro Hospitalar e Universitario de Coimbra, Coimbra, Portugal; 14 Dept of Respiratory Medicine, Faculty of Medicine, University of Crete, Crete, Greece; 15 Trial Form Support S.L., Barcelona, Spain; 16 Boehringer Ingelheim B.V., Amsterdam, The Netherlands; 17 Dept of Respiratory Medicine, Leuven University Hospital, Leuven, Belgium

## Abstract

The PERSEIDS study aimed to estimate incidence/prevalence of interstitial lung diseases (ILDs), fibrosing interstitial lung diseases (F-ILDs), idiopathic pulmonary fibrosis (IPF), systemic sclerosis-associated ILD (SSc-ILD), other non-IPF F-ILDs and their progressive-fibrosing (PF) forms in six European countries, as current data are scarce.

This retrospective, two-phase study used aggregate data (2014–2018). In Phase 1, incident/prevalent cases of ILDs above were identified from clinical databases through an algorithm based on codes/keywords, and incidence/prevalence was estimated. For non-IPF F-ILDs, the relative percentage of subtypes was also determined. In Phase 2, a subset of non-IPF F-ILD cases was manually reviewed to determine the percentage of PF behaviour and usual interstitial pneumonia-like (UIP-like) pattern. A weighted mean percentage of progression was calculated for each country and used to extrapolate incidence/prevalence of progressive-fibrosing ILDs (PF-ILDs).

In 2018, incidence/10^5^ person-years ranged between 9.4 and 83.6 (ILDs), 7.7 and 76.2 (F-ILDs), 0.4 and 10.3 (IPF), 6.6 and 71.7 (non-IPF F-ILDs), and 0.3 and 1.5 (SSc-ILD); and prevalence/10^5^ persons ranged between 33.6 and 247.4 (ILDs), 26.7 and 236.8 (F-ILDs), 2.8 and 31.0 (IPF), 22.3 and 205.8 (non-IPF F-ILDs), and 1.4 and 10.1 (SSc-ILD). Among non-IPF F-ILDs, sarcoidosis was the most frequent subtype. PF behaviour and UIP-like pattern were present in a third of non-IPF F-ILD cases each and hypersensitivity pneumonitis showed the highest percentage of progressive behaviour. Incidence of PF-ILDs ranged between 2.1 and 14.5/10^5^ person-years, and prevalence between 6.9 and 78.0/10^5^ persons.

To our knowledge, PERSEIDS is the first study assessing incidence, prevalence and rate of progression of ILDs across several European countries. Still below the threshold for orphan diseases, the estimates obtained were higher and more variable than reported in previous studies, but differences in study design/population must be considered.

## Abstract 2

**The PERSEIDS study provides updated and detailed epidemiological data for ILDs across six European countries. Prevalences ranged between 26.7–236.8 (F-ILDs), 2.8–31.0 (IPF), 22.3–205.8 (non-IPF F-ILDs). A third of non-IPF F-ILD cases showed PF behaviour.** https://bit.ly/3wLH7pF

## Introduction

Interstitial lung diseases (ILDs) display varying degrees of inflammation, fibrosis or both, and a wide range of clinical courses and prognoses. Idiopathic pulmonary fibrosis (IPF) is the prototypic progressive-fibrosing ILD (PF-ILD), characterised by a usual interstitial pneumonia (UIP) pattern on high-resolution computed tomography (HRCT) or lung biopsy [1]. Its pulmonary fibrosis (PF) behaviour is associated with a significant reduction in quality of life and early mortality [2, 3]. Although the introduction of antifibrotics in the last decade has improved survival among patients with IPF to a median of 5 years, it remains the ILD with the worst prognosis [4]. Non-IPF fibrosing ILDs (F-ILDs) may have a prognosis similar to IPF when progression and/or UIP are present [5]. Progression of non-IPF F-ILDs has been associated with higher healthcare resource use and worst quality of life [6]. Of non-IPF F-ILD subtypes, systemic sclerosis-associated ILD (SSc-ILD) is of particular interest as it presents early in the disease course [7] and impacts both mortality and quality of life [8, 9].

Despite their major impact on mortality, quality of life and resource use, in Europe the epidemiological data available for ILDs and particularly their PF forms is scarce [10, 11]. Therefore, the primary aim of the PERSEIDS study was to obtain detailed information on the epidemiology of ILDs in six European countries.

## Design and methods

**FIGURE 1:**
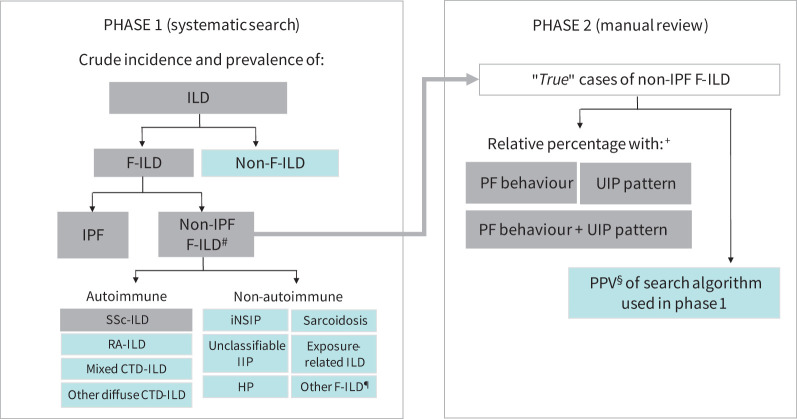
Study design. Grey squares correspond to primary outcomes and light blue squares to secondary outcomes. CTD-ILD: connective tissue disease-associated interstitial lung disease; F-ILD: fibrosing interstitial lung disease; HP: hypersensitivity pneumonitis; ILD: interstitial lung disease; iNSIP: idiopathic nonspecific interstitial pneumonia; IPF: idiopathic pulmonary fibrosis; non-IPF F-ILD: fibrosing interstitial lung disease other than idiopathic pulmonary fibrosis; PF-ILD: progressive-fibrosing interstitial lung disease; RA-ILD: rheumatoid arthritis-associated interstitial lung disease; SSc-ILD: systemic sclerosis-associated interstitial lung disease; PPV: positive predictive value; IIP: idiopathic interstitial pneumonia; UIP: usual interstitial pneumonia. ^#^: secondary outcomes of Phase 1 also included the relative percentage of each non-IPF F-ILD subtype. ^¶^: unspecified ILDs and other ILDs with fibrosis, including ICD-10 codes J84.10 (*Pulmonary fibrosis, unspecified*) and J84.89 (*Other specified interstitial pulmonary diseases*), ICD-9 codes 515 (*Postinflammatory pulmonary fibrosis*) and 516.9 (*Unspecified alveolar and parietoalveolar pneumonopathy*) and keywords such as “interstitial pulmonary disease”, “lung fibrosis”, “fibrotic lung”, among others (see supplementary material Section A). ^+^: also in Phase 2, a weighted mean percentage of PF behaviour was calculated for each country and used to extrapolate incidence and prevalence of PF-ILD. ^§^: the PPVs obtained in each country were used to adjust the incidence and prevalence estimates obtained in Phase 1 and Phase 2.

A retrospective, database study with a two-phase design (figure 1) was conducted in pulmonary and rheumatology departments at 14 centres in Belgium, Denmark, Finland, Greece, Norway and Portugal (table 1). The study was based on data collected between January 1, 2014 and December 31, 2018, which was extracted between May 15, 2019 and August 31, 2020. As only aggregate data were used, the Ethics Committee at each centre issued a waiver for informed consent.

**TABLE 1:** Characteristics of the participating centres

**Country**	**Centre**	**Centre level^#^**	**Reference centre**	**Department**	**Phase**	**Data source Phase 1**	**Algorithm based on**	**Incident cases reported for**	**Prevalent cases reported for**	**Cases reviewed in Phase 2**
**Belgium**	Leuven University Hospital	Tertiary	Yes	Pulmonary	2	NA	NA	NA	NA	100
	Ghent University Hospital	Tertiary	Only for SSc-ILD^¶^	Rheumatology	1+2	Departmental database	NA	2015–2018	2014–2018	100
	Liege University Hospital Centre	Tertiary	No	Pulmonary^+^	1	Institutional database (EMR)	ICD-10 Keywords	2014–2018	2014–2018	NA
**Denmark**	Lillebælt Hospital	Tertiary	No	Pulmonary	1+2	National registry^§^	Local codes	2014–2017	2014–2017	100
**Finland**	Turku University Hospital	Secondary	Yes	Pulmonary	1+2	Regional registry^ƒ^	ICD-10 Keywords	2014–2018	2014–2018	166
**Greece**	Heraklion University Hospital	Tertiary	Yes	Pulmonary	1	Institutional database (EMR)	ICD-10 Keywords	2014–2018	2014–2018	NA
	University Hospital of Larissa	Tertiary	Yes	Pulmonary^+^	1+2	Institutional database (EMR)	ICD-10 Keywords	2014–2018	2014–2018	118
	General Hospital of Thessaloniki	Tertiary	Yes	Pulmonary	1+2	Institutional database (paper records)	ICD-10	2014–2018	2014–2018	100
	Athens Medical Centre	Secondary	Yes	Pulmonary	1+2	Institutional database (EMR)	ICD-10 Keywords	2015–2018	2015–2018	100
**Norway**	Oslo University Hospital	Tertiary	Yes	Rheumatology^+^	1+2	Institutional database (EMR)	ICD-10	2015–2018	2014–2018	153
**Portugal**	Coimbra Hospital and University Centre	Tertiary	Yes	Pulmonary^+^	1	Institutional database (EMR)	Keywords	2018	2018	NA
	São João University Hospital Centre	Tertiary	Yes	Pulmonary^+^	1+2	Institutional database (EMR)	ICD-10 Keywords	2014–2018	2014–2018	131
	Vila Nova de Gaia/Espinho Hospital Centre	Tertiary	No	Pulmonary^+^	1	Institutional database (EMR)	Keywords	2014–2018	2018	NA
	Beatriz Ângelo Hospital	Secondary	No	Pulmonary	1+2	Institutional database (EMR)	ICD-9 Keywords	2014–2018	2014–2018	159

EMR: electronic medical records; ICD-9/-10: International Classification of Diseases 9th/10th revision; NA: not applicable; SSc-ILD: systemic sclerosis-associated interstitial lung disease. ^#^: secondary: specialised centres receiving referrals from Primary Care; tertiary: specialised centres with recognised expertise, receiving referrals from both Primary Care and secondary centres; ^¶^: this was a reference centre for SSc-ILD, and only provided data for this condition. A systematic search was not required as a departmental database was available that included all patients with SSc-ILD presenting for assessment during each year; ^+^: the department could retrieve most cases of ILD managed at the centre; ^§^: Danish National Patient Registry [12]. As the coverage of the database was wider than that of Lillebælt Hospital, the manual review of Phase 2 was limited to patients listed in the Hospital's database; ^ƒ^: Hospital District of Southwest Finland Database. The data was extracted by the Auria Centre for Clinical informatics at Turku University Hospital [13]. The coverage area of the database matched that of the Turku University Hospital.

### Phase 1

The main objective was to investigate the annual incidence and prevalence of ILDs and their time trends over the study period. The focus was placed on F-ILDs as these show a greater risk of progression, but the epidemiology of non-F-ILDs was also assessed to provide a broad picture of ILDs in the participating countries. The primary outcome was crude incidence/prevalence of ILDs, F-ILDs, IPF, non-IPF F-ILDs and SSc-ILD by country and overall, for the whole study period and annually. Secondary outcomes were: a) crude incidence/prevalence of non-IPF F-ILD subtypes (other than SSc-ILD), including ILDs associated with rheumatoid arthritis (RA-ILD), mixed connective tissue diseases (mixed CTD-ILDs) and other connective tissue diseases (other CTD-ILDs), idiopathic nonspecific interstitial pneumonia (iNSIP), unclassifiable idiopathic interstitial pneumonias (uIIPs), hypersensitivity pneumonitis (HP), exposure-related ILDs, sarcoidosis and other F-ILDs; b) relative percentage of each non-IPF F-ILD subtype; and c) crude incidence/prevalence of non-fibrosing ILDs (non-F-ILDs).

To identify incident/prevalent cases of ILD, 10 of the 13 centres participating in Phase 1 conducted a systematic search of the institution's clinical database (nine electronic records, one paper), and the remaining three in other clinical databases (departmental database, regional registry and national registry, respectively) (table 1). The search encompassed all patients ≥18 years old listed in the database/registry at each year of the study period. To adapt to each centre's coding particularities, ILD cases could be identified by either 9th/10th revision of International Classification of Diseases (ICD-9/ICD-10) codes, local codes and/or keywords (table 1 and supplementary material Section A). These codes/keywords were searched in all centres following a common algorithm, specifically designed to classify cases in ILD subtypes and subsequently, in overarching ILD categories while avoiding duplicates (supplementary material Figure 1). The methodology for obtaining prevalence/incidence estimates (minimum and maximum), as well as alternative methods and adjustments applied due to centre/country particularities, are detailed in the supplementary material Section B. The relative percentages of non-IPF F-ILD subtypes were obtained by dividing the prevalent cases of each subtype by total prevalent cases of non-IPF F-ILD.

### Phase 2

The aim was to further characterise non-IPF F-ILDs. The primary outcome was the relative percentage of cases within each non-IPF F-ILD subtype presenting PF behaviour alone (without UIP-like pattern), UIP-like pattern alone (without PF behaviour), both PF behaviour and UIP-like pattern, and none of them, overall for all countries. Secondary outcomes included: a) crude prevalence/incidence of PF-ILDs by country and overall, for the whole study period and annually; b) positive predictive value (PPV) of the algorithm used in Phase 1, by centre and country; and c) adjusted (by PPV) values for incidence/prevalence estimates obtained in both study phases.

Each of the 10 centres participating in Phase 2 reviewed the medical records of their first 100 prevalent non-IPF F-ILD cases identified in Phase 1, sorted by date of healthcare encounter from January 1, 2016 onwards.

The review encompassed the pulmonary function tests and HRCT results available in each patient's file, and either confirmed (true positive) or ruled out (false positive) the presence of the fibrosing (non-IPF) condition. The primary outcome was determined on true positives, and it was calculated that at least 80 were required to reach enough precision (supplementary material Section C). If necessary, centres could review additional cases to the first 100 until reaching this number. These additional cases were considered when calculating the relative percentages of the primary outcome.

For the primary outcome, PF behaviour was defined as a relative decline ≥10% in forced vital capacity (FVC % predicted) within the 2-year period following the healthcare encounter, or between 5 and <10% but with any of the following: ≥1 ILD-related hospitalisation (excluding emergency visits), increasing extent of fibrosis on HRCT, starting or increasing oxygen use, or death due to respiratory event. Owing to the lack of uniformly accepted criteria for PF-ILD, the use of relative decline in FVC was based on previously published expert recommendations [14]. The definition used for UIP-like pattern essentially equated to a UIP or probable UIP pattern: honeycomb lung destruction with basal and peripheral predominance in the absence of atypical features, and/or presence of reticular abnormality and traction bronchiectasis consistent with fibrosis with basal and peripheral predominance in the absence of atypical features. The incidence/prevalence of PF-ILDs in each country was obtained by multiplying non-IPF F-ILD incidence/prevalence estimates by the country percentage of PF behaviour. A weighted percentage was used to account for over-representation of certain subtypes in reference centres, and for differences in the proportion of PF behaviour among subtypes. To obtain the weighted percentage, the number of cases of each subtype were divided by total cases of non-IPF F-ILD and multiplied by the percentage of progression of the subtype (pooled for all countries), and results for all subtypes were summed.

True and false positives obtained during the Phase 2 review were also used to assess the specificity of the search algorithm at each centre, by calculating its PPV. The PPV was obtained by dividing true positives by the sum of true positives and false positives. Only the first 100 prevalent cases reviewed at each centre (not additional ones, if any) were considered. PPVs of centres in each country were averaged to obtain a country PPV. Simple (not weighted) means were calculated under the assumption that each centre's PPV contributed equally to the country mean.

Finally, to account for possible overestimation of incidences/prevalences arising from the use of codes and/or keywords in the systematic search, crude estimates for each country were multiplied by the country PPV to obtain adjusted estimates. Some exceptions to this methodology were made based on country particularities (supplementary material Section D).

### Statistical methods

Sample size considerations are presented in the supplementary material Section C. Continuous variables were summarised as means and standard deviations; or medians, ranges and interquartile ranges (IQR). Categorical variables were summarised as absolute and relative frequencies, and 95% confidence intervals. Pairwise comparisons were conducted to assess differences in the percentage of progression between non-IPF F-ILD subtypes and centre levels (secondary *versus* tertiary). Significance was set at the 0.05 level.

Missing values were completed when possible (see supplementary material Section B, *Alternative methods and adjustments*). When not, estimates were obtained based on available data.

The sensitivity analyses performed, and their corresponding statistical methods, are described in the supplementary material Section E.

## Results

### Positive predictive value for the search algorithm

The PPV was >70% in all countries except for Finland (49%). The centre/country PPVs are provided in the supplementary material sTable 1.

### Incidence and prevalence of ILDs

**FIGURE 2:**
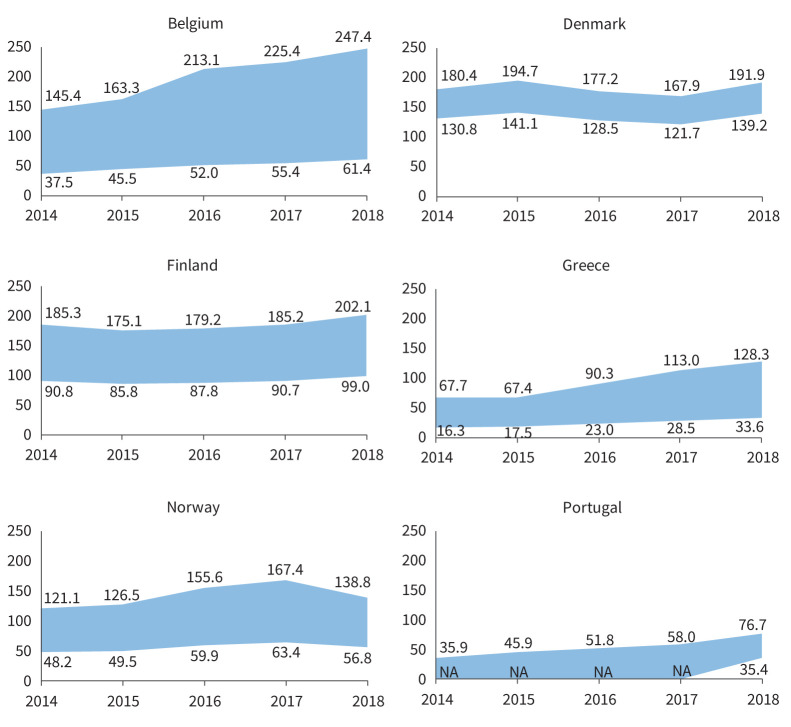
Annual prevalence (per 10^5^ persons) of ILDs in the participating countries during the study period (2014–2018). Areas show the widest variability observed in the primary plus sensitivity analyses. In Belgium, Greece, Norway and Portugal, this means the range between the minimum adjusted and the maximum crude estimates. In Denmark and Finland, there was only one participating centre that searched a national or regional database (respectively), so there were no reference and extended populations, but a single population (*i.e.* no maximum–minimum estimates, but a single estimate). In both countries, the area shows the range between the single crude and adjusted estimates. In Portugal, only one of the participating centres reported an extended population, and only for 2018. Therefore, minimum estimates could not be obtained for 2014–2017. For these years, the area shows the range from 0 to maximum crude estimates. ILDs: interstitial lung diseases; NA: not available.

**FIGURE 3:**
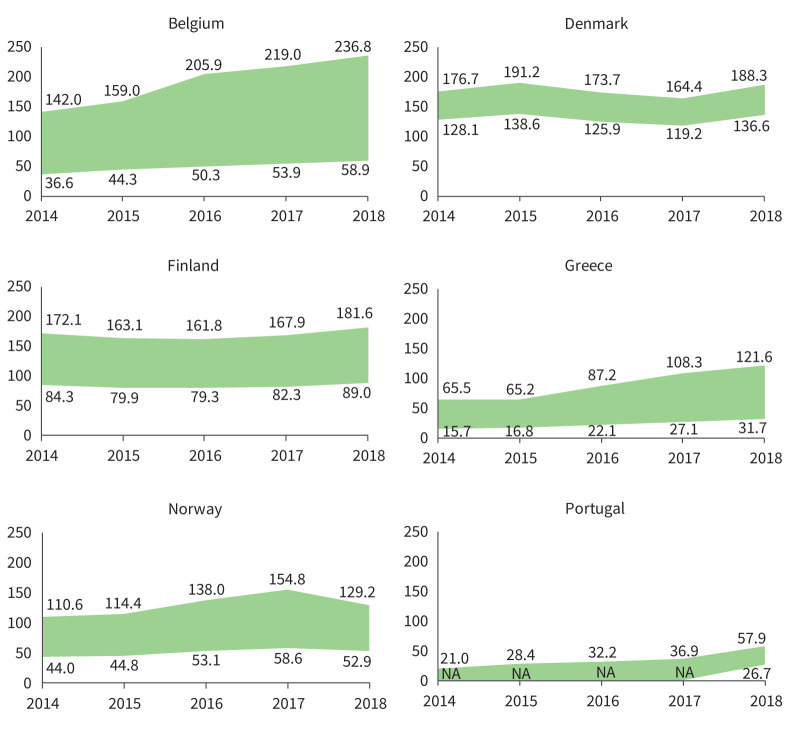
Annual prevalence (per 10^5^ persons) of F-ILDs in the participating countries during the study period (2014–2018). Areas show the widest variability observed in the primary plus sensitivity analyses. In Belgium, Greece, Norway and Portugal, this means the range between the minimum adjusted and the maximum crude estimates. In Denmark and Finland, there was only one participating centre that searched a national or regional database (respectively), so there were no reference and extended populations, but a single population (*i.e.* no maximum–minimum estimates, but a single estimate). In both countries, the area shows the range between the single crude and adjusted estimates. In Portugal, only one of the participating centres reported an extended population, and only for 2018. Therefore, minimum estimates could not be obtained for 2014–2017. For these years, the area shows the range from 0 to maximum crude estimates. F-ILDs: fibrosing interstitial lung diseases; NA: not available.

**FIGURE 4:**
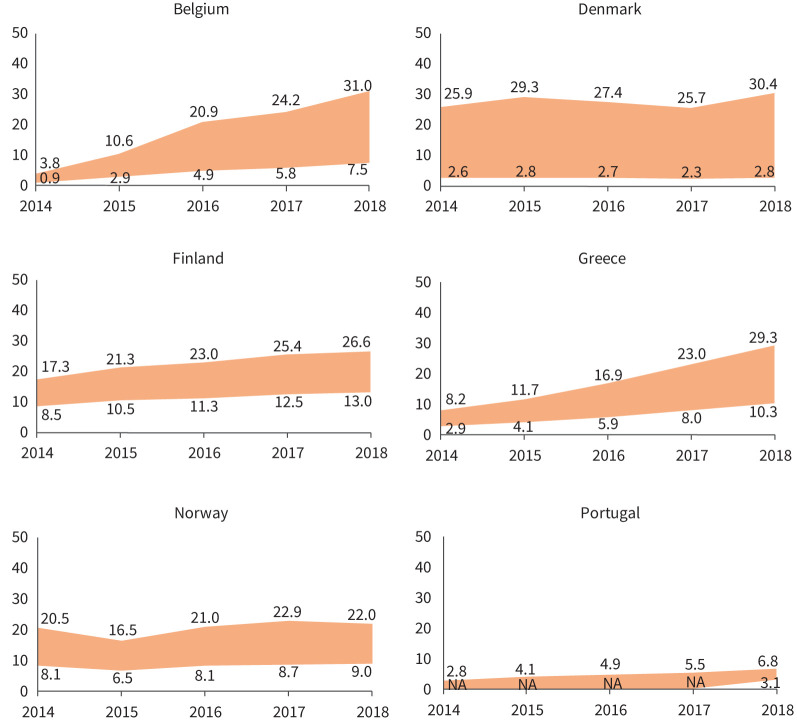
Annual prevalence (per 10^5^ persons) of IPF in the participating countries during the study period (2014–2018). Areas show the widest variability observed in the primary plus sensitivity analyses. In Belgium, Greece, Norway and Portugal, this means the range between the minimum adjusted and the maximum crude estimates. In Denmark and Finland, there was only one participating centre that searched a national or regional database (respectively), so there were no reference and extended populations, but a single population (*i.e.* no maximum–minimum estimates, but a single estimate). In both countries, the area shows the range between the single crude and adjusted estimates. In Portugal, only one of the participating centres reported an extended population, and only for 2018. Therefore, minimum estimates could not be obtained for 2014–2017. For these years, the area shows the range from 0 to maximum crude estimates. IPF: idiopathic pulmonary fibrosis; NA: not available.

**FIGURE 5:**
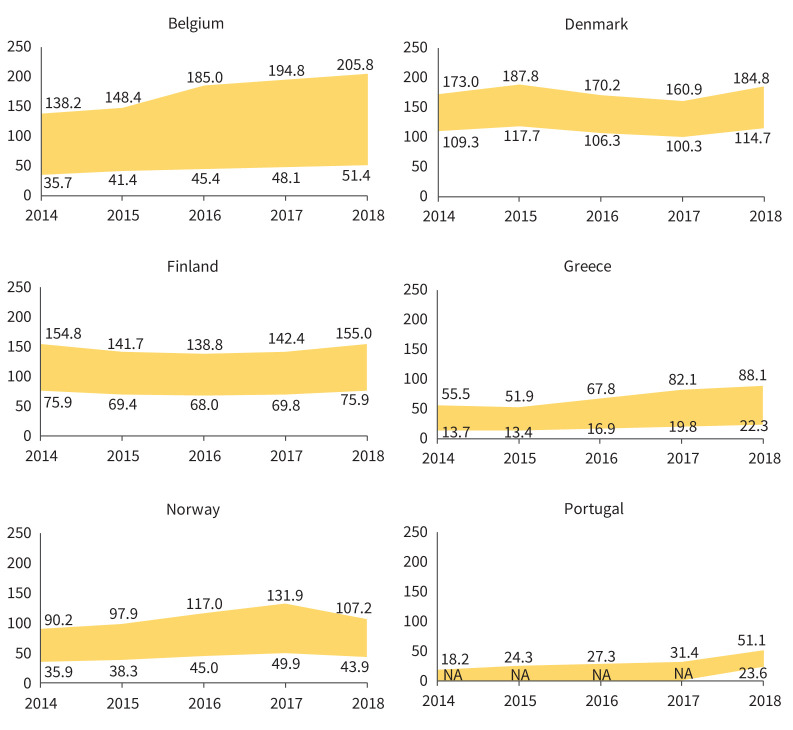
Annual prevalence (per 10^5^ persons) of non-IPF F-ILDs in the participating countries during the study period (2014–2018). Areas show the widest variability observed in the primary plus sensitivity analyses. In Denmark and Finland, there was only one participating centre that searched a national or regional database (respectively), so there were no reference and extended populations, but a single population (*i.e.* no maximum–minimum estimates, but a single estimate). In both countries, the area shows the range between the single crude and adjusted estimates. In Belgium, Greece, Norway and Portugal, this means the range between the minimum adjusted and the maximum crude estimates. In Portugal, only one of the participating centres reported an extended population, and only for 2018. Therefore, minimum estimates could not be obtained for 2014–2017. For these years, the area shows the range from 0 to maximum crude estimates. F-ILDs: fibrosing interstitial lung diseases; IPF: idiopathic pulmonary fibrosis; NA: not available.

In 2018, the latest year assessed, the lowest (minimum adjusted by PPV) incidence estimates for ILDs, F-ILDs, non-IPF F-ILDs and SSc-ILD were obtained in Portugal, and for IPF in Denmark. The highest (maximum crude) incidence estimates for ILDs, F-ILDs and non-IPF F-ILDs were obtained in Belgium, for IPF in Denmark and for SSc-ILD in Norway. The overall (mean for all countries) incidence (cases /10^5^ person-years) ranged between 20.0 and 42.5 (ILDs), 17.9 and 38.3 (F-ILDs), 2.1 and 6.3 (IPF), 14.7 and 33.9 (non-IPF F-ILDs), and 0.5 and 1.0 (SSc-ILD) (supplementary material sTable 2). The annual prevalences of ILDs in each country are presented in figures 2–5 and in supplementary material sFigure 2. The overall prevalence (cases/10^5^ persons) ranged between 72.1 and 164.2 (ILDs), 66.8 and 152.6 (F-ILDs), 7.8 and 24.3 (IPF), 56.2 and 132.0 (non-IPF F-ILDs) and 2.8 and 5.7 (SSc-ILD). Throughout the study period, incidence/prevalence were generally stable within each country, with few exceptions in Finland, Belgium and Greece (supplementary material sTable 2 and sTable 3).

### Incidence and prevalence of non-IPF F-ILD subtypes

**FIGURE 6:**
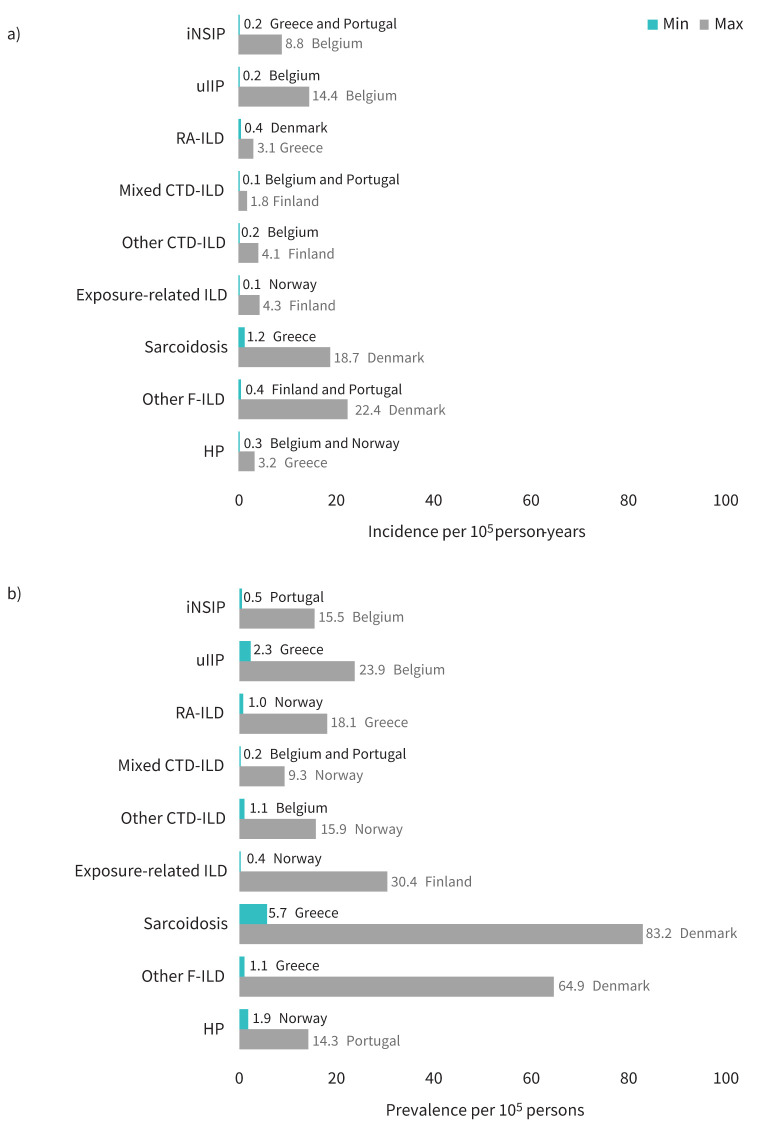
a) Incidence and b) prevalence of non-IPF F-ILD subtypes in 2018 (all countries). Minimum and maximum values show the widest variability observed in the primary plus sensitivity analyses (*i.e.* minimum adjusted–maximum crude) and across all participating countries. Countries where these minimum and maximum values were observed are indicated alongside data labels. CTD-ILD: connective tissue disease-associated interstitial lung disease; F-ILDs: fibrosing interstitial lung disease; HP: hypersensitivity pneumonitis; iNSIP: idiopathic nonspecific interstitial pneumonia; IPF: idiopathic pulmonary fibrosis; RA-ILD: rheumatoid arthritis-associated interstitial lung disease; SSc-ILD: systemic sclerosis-associated interstitial lung disease; uIIP: unclassifiable idiopathic interstitial pneumonia.

Figure 6 shows the lowest and highest incidence/prevalence estimates for non-IPF F-ILD subtypes found across countries in 2018. Incidences remained generally stable throughout the study period within each country, though seemed to decrease for exposure-related ILDs in Finland and to increase for other F-ILDs in Belgium, with prevalences changing accordingly. Other apparent changes in prevalence were an increase of other F-ILD in Denmark and sarcoidosis in Belgium and Portugal, and a decrease of sarcoidosis in Norway. The remaining prevalence estimates were rather constant (supplementary material sTable 4 and sTable 5).

### Relative percentage of non-IPF F-ILD subtypes

In 2018, sarcoidosis was the most frequent non-IPF F-ILD subtype in most countries (52.6% Denmark, 39.2% Finland, 36.1% Portugal and 25.6% Greece), except for Belgium (RA-ILD, 46.0%) and Norway (other F-ILD, 20.8%). In these two countries, however, sarcoidosis was the second most common subtype (15.6% in Belgium; 14.8% in Norway, along with 14.9% of other CTD). In the remaining countries, the second most common subtypes were other F-ILDs (24.3% Denmark), exposure-related ILDs (21.2% Finland) and HP (22.2% Portugal, 18.0% Greece). The most prevalent conditions within the other F-ILD category were only assessed in Denmark; in 2017, they were unspecified ILDs (DJ849, 49.5%) and other ILDs with fibrosis (DJ841, 37.6%). Relative percentages annually and for the whole study period are provided in the supplementary material sTable 6.

### Incidence and prevalence of non-F-ILDs

The minimum adjusted/maximum crude incidence of non-F-ILDs by country and overall for the whole study period and annually is shown in supplementary material sTable 7.

### Relative percentage of PF behaviour and UIP-like pattern

**FIGURE 7:**
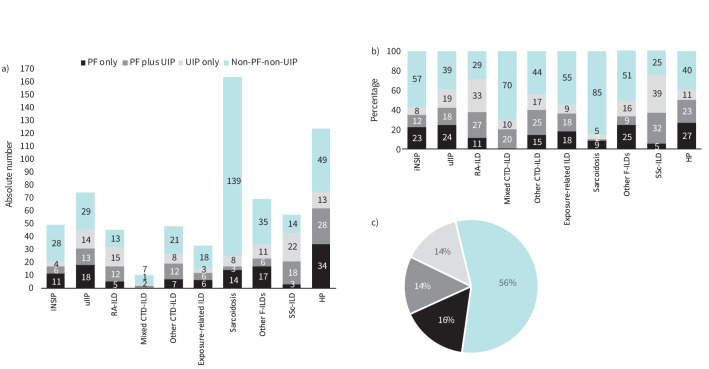
a) Absolute number and b) relative percentage in each non-IPF F-ILD subtype, and c) relative percentage in total non-IPF F-ILDs of cases with PF behaviour only, UIP-like pattern only, both and none (all countries). Percentages were calculated based on the total number of cases for each subtype (a and b) or the total number of cases of non-IPF F-ILDs (c). Only centres participating in Phase 2 and reporting complete data were considered (number of cases reviewed in Phase 2 at each centre are shown in parentheses): Belgium – Leuven (100); Denmark – Lillebælt (100); Finland – Turku (166); Greece – Larissa (118) and Thessaloniki (100); Portugal – São João (131) and Beatriz Angelo (159). CI: confidence interval; CTD-ILD: connective tissue disease-associated interstitial lung disease; F-ILDs: fibrosing interstitial lung disease; HP: hypersensitivity pneumonitis; iNSIP: idiopathic nonspecific interstitial pneumonia; IPF: idiopathic pulmonary fibrosis; PF: progressive-fibrosing; RA-ILD: rheumatoid arthritis-associated interstitial lung disease; SSc-ILD: systemic sclerosis-associated interstitial lung disease; uIIP: unclassifiable idiopathic interstitial pneumonia; UIP: usual interstitial pneumonia.

Absolute numbers and relative percentages of non-IPF F-ILD subtypes are shown in figure 7a and b, and full data are provided in the supplementary material sTable 8. Of total non-IPF F-ILD cases, approximately a third showed PF behaviour; 43% of these had a UIP-like pattern (figure 7c). Of note, the non-IPF F-ILD case-mix in Phase 2 was different from Phase 1, with more HP, SSc-ILD, uIIP and iNSIP cases reviewed in Phase 2 compared to the relative distribution of subtypes in Phase 1. Also, the case-mix was different in tertiary centres *versus* secondary, with more RA-ILD and SSc-ILD cases reviewed in the former. Pairwise comparisons revealed significant differences in the percentage of PF behaviour between subtypes (supplementary material sTable 9), but not between tertiary and secondary centres (p=0.6181).

### Incidence and prevalence of PF-ILDs

**FIGURE 8:**
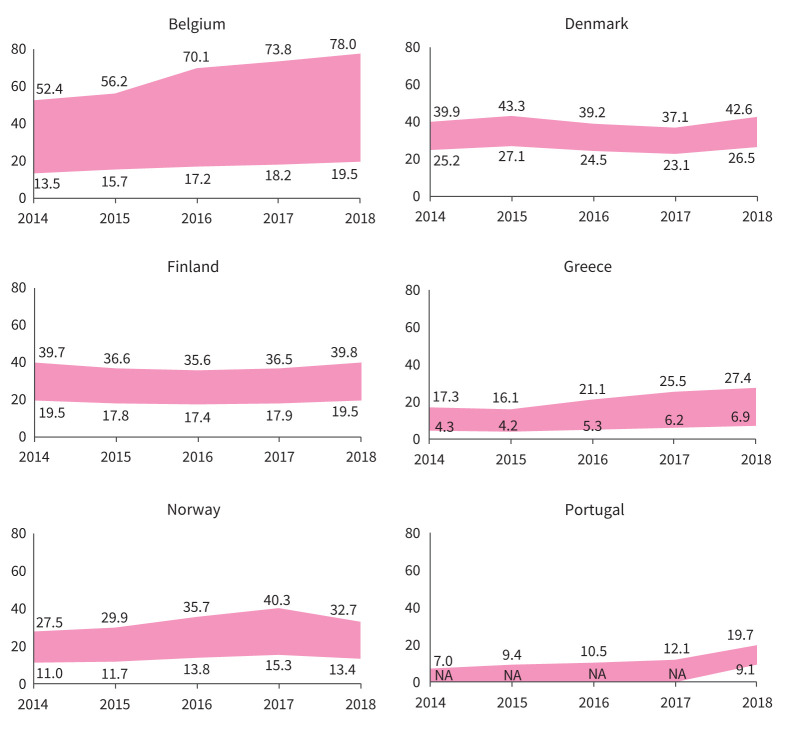
Annual prevalence (per 10^5^ persons) of PF-ILDs in the participating countries during the study period (Primary analysis). Areas show the widest variability observed in the primary plus sensitivity analyses. In Belgium, Greece, Norway and Portugal, this means the range between the minimum adjusted and the maximum crude estimates. In Denmark and Finland, there was only one participating centre that searched a national or regional database (respectively), so there were no reference and extended populations, but a single population (*i.e.* no maximum–minimum estimates, but a single estimate). In both countries, the area shows the range between the single crude and adjusted estimates. In Portugal, only one of the participating centres reported an extended population, and only for 2018. Therefore, minimum estimates could not be obtained for 2014–2017. For these years, the area shows the range from 0 to maximum crude estimates. NA: not available; PF-ILDs: progressive-fibrosing interstitial lung diseases.

The incidence in 2018 across the participating countries, assessed with the primary methodology, is show in figure 8. The overall incidence ranged between 4.4 and 8.6/10^5^ person-years and prevalence between 15.8 and 40.0/10^5^ persons. Supplementary material sTable 10 (incidence) and sTable 11 (prevalence) show the widest variability observed in the primary and sensitivity analyses, annually and for the whole study period. Within each country, the results of the sensitivity analyses were very similar, and the results for the primary analysis were within the range defined by sensitivity analyses. The I^2^-statistic obtained in the random effects model for Method 3 (90%) (see supplementary material Section E) revealed substantial heterogeneity in PF behaviour among countries.

## Discussion

PERSEIDS addressed the paucity of detailed and updated incidence/prevalence data for ILDs and its PF forms. The study has various strengths: the parallel implementation in six countries and the participation of both rheumatology and pulmonology units provides a broad, contemporaneous picture of the burden of ILDs across countries and grants a study population large enough to obtain sufficiently precise initial estimates. Also, the period of 5 years considered was wide enough to observe temporal trends and between-country differences.

The few epidemiological studies on ILDs conducted so far in countries participating in PERSEIDS show quite homogeneous estimates. Incidence data were available for Belgium (1.0/10^5^ persons/year in 1998) [15], Greece (4.63/10^5^ persons/year in 2004) [16], Denmark (19.36–34.34/10^5^ person-years between 1995 and 2005, 3.8–6.6 per 10^5^/year between 2003 and 2009) [17, 18], and the Nordics including Finland and Norway (1.4–20.0/10^5^ persons/year in 2009) [19]. The Belgian and Greek studies above also provided prevalence estimates for ILDs (6.27/10^5^ and 17.3/10^5^, respectively) [15, 16]. In contrast, PERSEIDS estimates were higher and more variable among countries. IPF incidence estimates available so far for Belgium (0.22/10^5^/year), Greece (0.93/10^5^/year), Denmark (7.27/10^5^ person-years between 1995 and 2005 and 1.3/10^5^/year between 2003 and 2009) and the Nordics (0.4–10/10^5^/year) were lower than in PERSEIDS, as was IPF prevalence reported previously for Belgium (1.25/10^5^) and Greece (3.38/10^5^) [15–19]. A Norwegian nationwide cohort study estimated the incidence of SSc-ILD at 1.0–1.3/10^5^ persons between 2005 and 2012 and the prevalence at 13/10^5^ persons in 2013 [20], both in line with PERSEIDS estimates for Norway. Nevertheless, comparing the results of prior studies with PERSEIDS is difficult due to several differences in data collection periods, data sources, population composition/size, disease definitions, diagnostic criteria, availability of HRCT scans, specialists involved or even the ILDs considered. Of note, despite the comparatively higher estimates, in PERSEIDS the overall prevalence of individual ILD subtypes (IPF and non-IPF F-ILDs) remained below the European Union threshold for orphan diseases (50/10^5^ persons [21]).

Highest maximum crude estimates for ILDs, F-ILDs and non-IPF F-ILDs were consistently found in Belgium, and lowest in Portugal. Multiple factors may explain these findings. In Belgium, Liege Hospital was the only centre providing data for all ILDs in Phase 1. As rheumatological ILDs are referred to this centre from all over the country, we adjusted the reference population in these ILDs only. However, non-rheumatological referrals cannot be ruled out, so there may have been overestimation of these conditions and of the overarching ILD categories. Other contributing factors may be the concentration of heavy industries in the province of Limburg [15] and the high percentage of smokers among the Belgian population [22]. As for Portugal, the largest city (Lisbon) was not represented, and most patients with ILD are routinely managed in several hospitals in this country, possibly hindering the ability of the participating centres to identify incident/prevalent cases more accurately. The prevalence of other F-ILDs was high in Denmark, probably because the nationwide, comprehensive search conducted had a greater potential to detect these subtypes. For IPF, interestingly, the highest and lowest incidence estimates were consistently observed in the same country (Denmark) throughout the study period. In this country, maximum incidence was obtained based on incident cases identified by the systematic search (probably overestimated due to coding issues) and minimum incidence based on down-adjusted incident cases (see supplementary material Section B). The extent of overestimation and the strictness of the adjustment applied may explain the highest/lowest estimates compared to other countries.

Incidence of ILDs was relatively high compared to prevalence. This may point to a high mortality rate; but there may be other factors contributing, such as the aged population structure in Europe [17] and a better ascertainment of ILDs [23], in part due to an increasing use of HRCT [24]. Throughout the study period, incidence/prevalence were rather constant in most countries. The changes observed in Finland and Belgium seemed mainly driven by non-IPF F-ILDs, with exposure-related ILDs showing a downward trend in Finland, and other F-ILD and sarcoidosis increasing in Belgium. This suggests changes in occupational exposure and in ILDs classification. In Greece, the increasing prevalence (but not incidence) of F-ILD seemed to be driven by both IPF and non-IPF F-ILDs, perhaps pointing to improved survival. The relevance of these trends is not clear, as the study was not designed to assess the statistical significance of changes throughout the study period.

The relative distribution of non-IPF F-ILDs subtypes observed may help clinicians discern which patients should be screened for ILDs, to achieve prompt diagnoses and improved outcomes; and for the most prevalent subtypes, should trigger the development of proper recommendations for management, as they are currently lacking except for SSc-ILD [25]. In most countries, sarcoidosis was the most frequent subtype, as reported previously [10, 15, 16]. The exceptions were Belgium (RA-ILD) and Norway (other F-ILD). In Belgium, the only centre providing data for all subtypes in Phase 1 (Liege) was of reference for rheumatological ILDs (including RA-ILD), while in Norway, “other F-ILD” may have included other ILD subtypes due to the coding issues described in the supplementary material. The expected bias was reduced by using wider reference populations for calculating incidence/prevalence, applying a different PPV to rheumatological ILDs (Liège) and estimating F-ILDs incidence/prevalence based on data from surrounding countries (Norway), but some bias may have remained. The high percentage of exposure-related ILDs in Finland may due to its prominent ship-building industry [26], with its extensive use of asbestos throughout the 20th century [27]. The exposure–diagnosis lag-time may explain the still high proportion of exposure-related ILDs, while the effect of asbestos bans may be behind the progressive incidence/prevalence reduction observed in PERSEIDS. In Portugal, the high percentage of HP may be partly explained by the importance of the cork industry and the widespread practice of pigeon breeding [28, 29], both associated with HP [30–32]. The incidence of some non-IPF F-ILD subtypes exceeded that of IPF (sarcoidosis in most countries, but also other F-ILD in Belgium and Denmark, and iNSIP and uIIP in Belgium). The refinement of IPF definition and the improvement of diagnostic procedures in later years [33] may have reduced misdiagnoses of non-IPF F-ILDs as IPF, and is expected to further increase non-IPF F-ILDs estimates in the future.

Sarcoidosis was the most frequent non-IPF F-ILD subtype in Phase 1. Though rarely fibrotic in early stages, sarcoidosis was included among non-IPF F-ILDs in PERSEIDS because fibrosis ultimately affects 20% of patients with stage IV disease [14]. The codes/keywords used for sarcoidosis in Phase 1 were specific for lung involvement but could not discern between fibrotic and non-fibrotic disease, probably resulting in overestimated crude incidence/prevalence. This was later amended by the HRCT review in Phase 2, where false fibrotic cases were detected and accounted for (through the PPV) to adjust incidence/prevalence estimates for ILDs and PF-ILDS. The high proportion of PF behaviour in Phase 2 may be partly explained by differences in case-mix, which in Phase 2 favoured more progressive subtypes. PF behaviour definition in PERSEIDS was similar to the one used in the INBUILD trial [34], but being a retrospective study it was difficult to assess worsening of symptoms in patients with a moderate FVC decline (5 to <10% predicted). In these patients, surrogates such as hospitalisations and increasing fibrosis were used instead. PF behaviour was present in 30% of non-IPF F-ILD cases, as observed in the recent French PROGRESS study among patients not receiving antifibrotic treatment [35]. The percentage of PF behaviour among individual subtypes was generally consistent with that reported previously [10, 35, 36] except for SSc-ILD, though differences in PF behaviour definition must be considered [37]. Overall, PERSEIDS results point at subtypes more prone to progression (HP, other F-ILDs, uIIP and iNSIP) which could benefit most from a thorough follow-up and antifibrotic treatment. The proportion of UIP-like pattern in PERSEIDS was highest among SSc-ILD patients (70%), though generally <10% of SSc-ILDs show a definite UIP pattern [38, 39]. Also, UIP-like features were present in approximately a third of non-IPF F-ILD cases and in almost half of cases with PF behaviour. It must be considered that the UIP definition used in PERSEIDS included UIP-like HRCT patterns, and true UIP was not confirmed with histology. In the centre where most SSc-ILD cases with UIP-like pattern were reported, the percentage approached that previously reported for nonspecific idiopathic interstitial pneumonia (NSIP), a pattern far more common in SSc-ILD [40]. There is some overlap between NSIP (especially when fibrotic) and UIP features, which can be difficult to distinguish between [41]. The broader, UIP-like pattern used in PERSEIDS might have facilitated misclassification and thus the important overestimation of UIP in this centre.

Differences in the relative distribution of non-IPF F-ILD subtypes among countries and in their percentage of progression explain the variability in incidence/prevalence estimates of PF-ILDs, and reinforce the primary methodology chosen (weighted mean percentage of progression). Also, primary and sensitivity analyses gave similar results in most countries, further supporting the strength of results. Nevertheless, the primary approach had some limitations, as the weighted percentage was calculated by pooling progression data from all countries, and the I^2^ statistic revealed a considerable heterogeneity among them.

Despite the efforts to reduce bias, the study has other limitations. It was not always possible to use a homogeneous methodology due to centre/country particularities, which may limit the comparability of results. Coding particularities/issues may explain some of the variability found between countries, as it may have influenced the ability to capture incident/prevalent cases and may have led to more misclassification in specific countries. Some centres (*e.g.* Portugal, Norway) did not provide incident/prevalent cases for the whole study period, precluding comparisons among countries for missing years and possibly affecting overall estimates. Between-country differences in ILD management, previously highlighted [18], also made comparisons difficult. Finally, the ratio of participating to total reference centres in each country and some participating centres being of reference for specific ILDs may have affected the representativeness of results at the country level. In this sense, future epidemiological studies should engage a higher percentage of centres managing ILDs within each country or use national registries where available.

Other limitations were not country-specific. ILDs are often rare and diagnosis can be difficult, leading to over/underestimation and misdiagnosis. Also, the use of ICD codes for ILDs has known limitations [42], but the PERSEIDS approach was reasonable as currently there is no standard methodology for ILD identification based on diagnostic codes. Some code/keywords for ILD-associated conditions were excluded from the search, but being extremely infrequent and/or unspecific, a relevant effect on results is not expected. Although the search algorithm was not previously validated, it was designed *ad hoc* following expert advice to maximise specificity and sensibility, and the high PPVs obtained in most countries are reassuring. The low PPV found in Finland may be partly explained by the ∼40% of sarcoidoses found in Phase 1, many of which were found to be non-fibrosing after the review of Phase 2. When calculating incidence/prevalence, populations to be used as denominators were not always clear, especially in centres receiving referrals, which can lead to under/overestimation. Furthermore, referrals were not always systematic, or varied during the study period. These uncertainties were addressed through sensitivity analyses wherever possible. Adjusting estimates by a PPV calculated based on non-IPF F-ILDs implied assuming the same rate of false positives across all ILDs, which may not be the case. Finally, non-IPF F-ILD cases reviewed during Phase 2 may represent a small proportion of total prevalent cases identified in the systematic search (especially in centres searching national or regional registries). However, as ≥100 consecutive cases were reviewed per centre, the sample is expected to be representative.

### Conclusions

To our knowledge, PERSEIDS is the first study assessing the epidemiology and progression of ILDs in parallel across several European countries. The incidence and prevalence of ILDs was higher and more variable between countries than previously reported, but below the threshold for orphan diseases. Overall, approximately a third of non-IPF F-ILDs cases showed a PF behaviour, and nearly half of those (43%) had a UIP-like pattern. The prevalence and incidence of PF-ILDs ranged between 2.1–14.5/10^5^ person-years and 6.9–78.0/10^5^ persons, with differences between countries probably explained by the relative distribution of non-IPF F-ILD subtypes and their different rates of progression.

## Supplementary material

10.1183/23120541.00597-2021.Supp1 **Please note:** supplementary material is not edited by the Editorial Office, and is uploaded as it has been supplied by the author.
Supplementary material 00597-2021.SUPPLEMENT
