# Supplementary material for: Epidemiology of interstitial lung diseases and their progressive-fibrosing behaviour in six European countries
Source: ERJ Open Res. 2022 Jan 24;8(1):00597-2021. doi: 10.1183/23120541.00597-2021 (PMC8784757; doi:10.1183/23120541.00597-2021)
Supplement: Supplementary file 1 [file 00597-2021.SUPPLEMENT.pdf]

SUPPLEMENTARY MATERIAL

SUPPLEMENTARY METHODS 2

Section A. List of codes/keywords used in the systematic search of Phase 1 2

Section B. Methodology for calculating prevalence, incidence and relative percentages in Phase 1 33

Section C. Sample size calculations 37

Section D. Exceptions to the general methodology for PPV calculation in Phase 2 38

Section E. Sensitivity analyses 38

SUPPLEMENTARY FIGURES 40

sFigure 1. Algorithm for the systematic search in Phase 1 40

sFigure 2. Annual prevalence (per 10^5 persons) of SSc-ILD in the participating countries during the study period (2014-2018) 41

SUPPLEMENTARY TABLES 42

sTable 1. PPVs by centre and country 42

sTable 2. Incidence per 10^5 person-years (95%CI) of ILDs, F-ILDs, IPF, non-IPF F-ILDs, and SSc-ILD in each country and overall, annually and for the whole study period 43

sTable 3. Prevalence per 10^5 persons (95%CI) of ILD, F-ILD, IPF, non-IPF F-ILD, and SSc-ILD in each country and overall, annually and for the whole study period 47

sTable 4. Incidence per 10^5 person-years (95%CI) of non-IPF F-ILD subtypes in each country and overall, annually and for the whole study period 51

sTable 5. Prevalence per 10^5 persons (95%CI) of non-IPF F-ILD subtypes in each country and overall, annually and for the whole study period 57

sTable 6. Relative percentage (95%CI) of each non-IPF F-ILD subtype in each country, annually and for the whole study period 63

sTable 7. Incidence per 10^5 person-years and prevalence per 10^5 persons (95%CI) of non-F-ILDs in each country and overall, annually and for the whole study period 67

sTable 8. Relative percentage (95% CI) of PF behaviour only, UIP-like pattern only, both or none among total non-IPF F-ILD cases and by subtype (all countries) 69

sTable 9. Exploratory p-values for pairwise comparisons of PF behaviour percentage between non-IPF F-ILD subtypes (all countries) 70

sTable 10. Incidence per 10^5 person-years (95%CI) of PF-ILDs in each country and overall, annually and for the whole study period. Primary analysis (Method 1) and sensitivity analyses (Methods 1b-3) 71

sTable 11. Prevalence per 10^5 persons (95%CI) of PF-ILDs in each country and overall, annually and for the whole study period. Primary analysis (Method 1) and sensitivity analyses (Methods 1b-3) 75

SUPPLEMENTARY METHODS

Section A. List of codes/keywords used in the systematic search of Phase 1

Codes

ICD-10[1]

Disease	***Underlying autoimmune disease***	***ILD Category***	***Underlying medical condition***
ILD	*Non-Autoimmune*	*ILD-iNSIP*	*J84.113 Idiopathic non-specific interstitial pneumonitis*
		ILD-IPF	*J84.112 Idiopathic pulmonary fibrosis*
		ILD-Unclassifiable IIP	*J84.111 Idiopathic interstitial pneumonia, not otherwise specified*
		ILD-Hypersensitivity Pneumonitis	*J67.x Hypersensitivity pneumonitis due to organic dust*
		ILD-Exposure-related ILD	*J60 Coal Workers' Pneumoconiosis*
			J61 Asbestosis
			J62.8 Pneumoconiosis due to other dust containing silica
			J63.x Pneumoconiosis due to other inorganic dusts
			J64 Unspecified pneumoconiosis
			J66.0 Byssinosis

[1] Some subtypes without a specific ICD-10 code were defined by the corresponding ICD-10CM, which contains more granularity, to account for variability in coding practices.

Disease	***Underlying autoimmune disease***	***ILD Category***	***Underlying medical condition***
		ILD-Sarcoidosis	*D86.0 Sarcoidosis of the lung*
			D86.2 Sarcoidosis of the lung and lymph nodes
		ILD-Other fibrosing	*J84.10 Pulmonary fibrosis, unspecified*
			J84.89 Other specified interstitial pulmonary diseases
			J84.9 Interstitial pulmonary disease, unspecified
		ILD-Other (non-fibrotic)	*J84.114 Acute interstitial pneumonitis*
			J84.116 Cryptogenic organizing pneumonia
			J84.117 Desquamative interstitial pneumonia
			D76.x Langerhans 'cell histiocytosis
			J84.82 Adult Pulmonary Langerhans histiocytosis
			J84.83 Surfactant mutations of the lung
			J84.115 Respiratory bronchiolitis interstitial lung disease
	Autoimmune/CTD-ILD	*ILD-Rheumatoid Arthritis associated ILD*	*M05.1 Rheumatoid lung disease with rheumatoid arthritis*
		ILD-SSc-ILD	*M34.81 Systemic sclerosis with lung involvement*
			M34.x at any time within the last 10 years + any fibrotic code (J84.10/J84.89/J84.9)
		ILD-Mixed Connective Tissue Disease	*M35.1 Other overlap syndromes*

Disease	***Underlying autoimmune disease***	***ILD Category***	***Underlying medical condition***
		ILD-Other diffuse CTD	*M32.13 Lung involvement in SLE*
			M33.01 Juvenile dermatopolymyositis w lung involvement
			M33.11 Other dermatopolymyositis w lung involvement
			M33.91 Dermatopolymyositis unsp w lung involvement
			M33.21 Polymyositis with respiratory involvement
			M35.02 Sicca syndrome with lung involvement

NOTE: ".x" in a code denotes that all numeric subcodes are included

ICD-9

Disease	***Underlying autoimmune disease***	***ILD Category***	***Underlying medical condition***
ILD	*Non-Autoimmune*	*ILD-iNSIP*	*516.32 Idiopathic non-specific interstitial pneumonitis*
		ILD-IPF	*516.31 Idiopathic pulmonary fibrosis*
		ILD-Unclassifiable IIP	*516.30 Idiopathic interstitial pneumonia, not otherwise specified*
		ILD-Hypersensitivity Pneumonitis	*495.x Hypersensitivity pneumonitis*
		ILD-Exposure-related ILD	*500 Coal Workers' Pneumoconiosis*
			501 Asbestosis
			502 Pneumoconiosis due to other silica or silicates

Disease	***Underlying autoimmune disease***	***ILD Category***	***Underlying medical condition***
			503 Pneumoconiosis due to other inorganic dust
			504 Pneumonopathy due to inhalation of other dust
			505 Pneumoconiosis, unspecified
		ILD-Sarcoidosis	*135 Sarcoidosis*
		ILD-Other fibrosing	*515 Postinflammatory pulmonary fibrosis*
			516.9 Unspecified alveolar and parietoalveolar pneumonopathy
		ILD-Other (non-fibrotic)	*516.33 Acute interstitial pneumonitis*
			516.36 Cryptogenic organizing pneumonia
			516.37 Desquamative interstitial pneumonia
			516.5 Adult Pulmonary Langerhans histiocytosis
			516.63 Surfactant mutations of the lung
			516.34 Respiratory bronchiolitis interstitial lung disease
	Autoimmune/CTD-ILD	*ILD-Rheumatoid Arthritis associated ILD*	*714.81 Rheumatoid lung*
		ILD-SSc-ILD	*517.2 Lung involvement in systemic sclerosis*
			710.1 at any time within the last 10 years + any fibrotic code (515/516.9)

**Disease**	**Underlying autoimmune disease**	**ILD Category**	**Underlying medical condition**
		ILD-Mixed Connective Tissue Disease	*710.8 Other specified diffuse diseases of connective tissue*
		ILD-Other diffuse CTD	*710.0 Systemic lupus erythematosus + 517.8 Lung involvement in other diseases classified elsewhere*
			710.3 Dermatomyositis + 517.8 Lung involvement in other diseases classified elsewhere
			710.4 Polymyositis + 517.8 Lung involvement in other diseases classified elsewhere
			710.2 Sicca syndrome + 517.8 Lung involvement in other diseases classified elsewhere

NOTE: ".x" in a code denotes that all numeric subcodes are included

Keywords

English transliteration

**Disease**	**Underlying autoimmune disease**	**ILD Category**	**Underlying medical condition**
ILD	*Non-Autoimmune*	*ILD-iNSIP*	*Idiopathic non-specific interstitial pneumonitis*
			NSIP
			Fibrotic NSIP
			Fibrotic non-specific interstitial pneumonia
			Fibrotic non specific interstitial pneumonia

Disease	***Underlying autoimmune disease***	***ILD Category***	***Underlying medical condition***
		ILD-IPF	*Idiopathic pulmonary fibrosis*
			IPF
		ILD-Unclassifiable IIP	*Idiopathic interstitial pneumonia, not otherwise specified*
			Unclassifiable IIP
		ILD-Hypersensitivity Pneumonitis	*Hypersensitivity pneumonitis*
			Hypersensitivity pneumonia
			Extrinsic allergic alveolitis
		ILD-Exposure-related ILD	*Coal Workers*
			Asbestosis
			Pneumoconiosis
			Byssinosis
		ILD-Sarcoidosis	*Sarcoidosis of the lung*
			Sarcoidosis + any ILD-other fibrosing keyword
		ILD-Other fibrosing	*Interstitial pulmonary disease*
			Interstitial pulmonary diseases
			Interstitial lung disease
			Pulmonary fibrosis
			Fibrotic interstitial lung disease

Disease	***Underlying autoimmune disease***	***ILD Category***	***Underlying medical condition***
			Pulmonary fibrosis
			Lung fibrosis
			Fibrosing alveolitis
			Interstitial pneumonia
			Interstitial pneumonias
			Interstitial pneumonitis
			Fibrotic lung
			IIP
			Idiopathic interstitial pneumonia
			Fibrotic interstitial pneumonia
			ILD
			Idiopathic lung disease
			Pulmonary fibrotic
			Restrictive lung disease
			UIP
			Usual interstitial pneumonia
			Diffuse parenchymal lung disease
			Interstitial alveolitis

Disease	***Underlying autoimmune disease***	***ILD Category***	***Underlying medical condition***
		ILD-Other (non-fibrotic)	*Acute interstitial pneumonitis*
			AIP
			Cryptogenic organizing pneumonia
			Desquamative interstitial pneumonia
			Langerhans 'cell histiocytosis
			Langerhans histiocytosis
			Surfactant mutations of the lung
			Respiratory bronchiolitis
	Autoimmune/CTD-ILD	*ILD-Rheumatoid Arthritis associated ILD*	*Rheumatoid lung disease*
			RA-ILD
			RA with lung involvement
		ILD-SSc-ILD	*Systemic sclerosis with lung involvement*
			Any ILD-other fibrosing keyword + any SSc keyword at any time within the last 10 years
		ILD-Mixed Connective Tissue Disease	*Mixed CTD-ILD*
			MCTD-ILD
		ILD-Other diffuse CTD	*SLE*
			Juvenile dermatopolymyositis w lung involvement

Disease	*Underlying autoimmune disease*	*ILD Category*	*Underlying medical condition*
			Other dermatopolymyositis w lung involvement
			Dermatopolymyositis unsp w lung involvement
			Polymyositis with respiratory involvement
			Sicca syndrome with lung involvement
			Sjogren

Belgium - French

Maladie	*Maladie auto-immune sous-jacente*	*MPI Catégorie*	*Condition médicale sous-jacente*
MPI	*Non auto-immune*	*MPI-PINS*	*Pneumopathie interstitielle non spécifique*
			PINS
			PINS fibrotique
			Fibrotic pneumopathie interstitielle non spécifique
		MPI -FPI	*Fibrose pulmonaire idiopathique*
			FPI
		MPI -Inclassable IIP	*Pneumonie interstitielle idiopathique, non spécifiée*
			Inclassable IIP

Maladie	Maladie auto-immune sous-jacente	MPI Catégorie	Condition médicale sous-jacente
		MIP- Pneumopathie d'hypersensibilité	Pneumopathie d'hypersensibilité
			Alvéolite allergique extrinsèque
		MPI- MPI liée à l'exposition	Charbonniers / pneumoconiose du charbon
			Asbestose
			Pneumoconiose
			Byssinose
		MPI- Sarcoïdose	Sarcoïdose du poumon
			Sarcoïdose + n'importe quel mot clé relative à MPI- autres fibroses
		MPI- autres fibroses	Maladie pulmonaire interstitielle / Maladies pulmonaires interstitielles
			Fibrose pulmonaire
			Maladie pulmonaire interstitielle fibrotique
			Alvéolite fibrosante
			Pneumonie interstitielle
			Pneumonies interstitielles
			Pneumopathie interstitielle
			Poumon fibrotique

Maladie	***Maladie auto-immune sous-jacente***	***MPI Catégorie***	***Condition médicale sous-jacente***
			PII
			Pneumonie interstitielle idiopathique
			Pneumonie interstitielle fibrotique
			MPI
			Maladie pulmonaire idiopathique
			Fibrotique pulmonaire
			Maladie pulmonaire restrictive
			UIP
			Pneumonie interstitielle usuelle
			Maladie pulmonaire diffuse du parenchyme
			Alvéolite interstitielle
		MPI- Autre (non fibrotique)	*Pneumopathie interstitielle aiguë*
			PIA
			Pneumonie organisatrice cryptogène
			Pneumonie interstitielle desquamative
			Histiocytose à cellules de Langerhans
			Histiocytose de Langerhans
			Mutations du surfactant du poumon

Maladie	***Maladie auto-immune sous-jacente***	***MPI Catégorie***	***Condition médicale sous-jacente***
			Bronchiolite respiratoire
	Auto-immune/MPI - Maladie du tissu conjonctif	*MPI- Maladie pulmonaire interstitielle associée à la polyarthrite rhumatoïde*	*Maladie pulmonaire rhumatoïde*
			PR-MPI
			Polyarthrite rhumatoïde avec atteinte pulmonaire
		MPI-SSc-MPI	*Sclérose systémique avec atteinte pulmonaire*
			N'importe quel mot clé relative à MPI ou autre "fibrosing" + autre mot clé relative à SSc à tout moment au cours des 10 dernières années
		MPI - Maladie mixte du tissu conjonctif	*Maladie mixte du tissu conjonctif*
		MPI- autres maladies diffuses du tissu conjonctif	*Lupus érythémateux disséminé LED*
			Dermatopolymyosite juvénile avec atteinte pulmonaire
			Autres dermatopolymyosites à atteinte pulmonaire
			Dermatopolymyosite non précisée avec atteinte pulmonaire
			Polymyosite avec atteinte respiratoire
			Syndrome de Sicca avec atteinte pulmonaire
			Sjogren / syndrome de Sjogren

Belgium - Dutch

Ziekte	*Onderliggende auto-immuunziekte*	*Categorie ILD*	*Onderliggende medische aandoening*
Interstitiële longziekte	*Niet auto-immuun*	*ILD-NSIP*	*Idiopathische niet-specifieke interstitiële pneumonitis*
			NSIP
			Fibrotische nsip
			Fibrotische niet-specifieke interstitiële pneumonie
		ILD-IPF	*Idiopathische pulmonale fibrose*
			IPF
		ILD-Niet-classificeerbare IIP	*Idiopathische interstitiële pneumonie, niet anders gespecificeerd*
			Niet-classificeerbare IIP
		ILD-Overgevoeligheidspneumonitis	*Overgevoeligheidspneumonitis*
			Overgevoeligheidspneumonie
			Extrinsieke allergische alveolitis
		ILD- Blootstelling Gerelateerde ILD	*Kolenarbeiders*
			Asbestose

Ziekte	***Onderliggende auto-immuunziekte***	***Categorie ILD***	***Onderliggende medische aandoening***
			Pneumoconiose
			Byssinosis
		ILD- Sarcoïdose	*Sarcoïdose van de long*
			Sarcoïdose + een ander ILD-fibrosing-sleutelwoord
		ILD-Ander fibrosing	*Interstitiële longziekte*
			Interstitiële longziekten
			Pulmonaire fibrose
			Fibrotische interstitiële longziekte
			Longfibrose
			Fibroserende alveolitis
			Interstitiële pneumonie
			Interstitiële pneumonieën
			Interstitiële pneumonitis
			Fibrotische long
			IIP
			Idiopathische interstitiële pneumonie
			Fibrotische interstitiële pneumonie
			ILD

Ziekte	***Onderliggende auto-immuunziekte***	***Categorie ILD***	***Onderliggende medische aandoening***
			Idiopathische longziekte
			Pulmonaire fibrotische
			Restrictieve longziekte
			UIP
			Usual interstitial pneumonia
			Diffuse parenchymale longziekte
			Interstitiële alveolitis
		ILD-Anders (niet-fibrotisch)	*Acute interstitiële pneumonitis*
			AIP
			Cryptogeen organiseren longontsteking
			Desquamative interstitiële pneumonitis
			Langerhans cel histiocytose
			Langerhans histiocytose
			Surfactant mutaties van de longen
			Luchtwegen bronchiolitis
	Auto immuun/CTD-ILD	*ILD- Reumatoïde artritis geassocieerde ILD*	*Reumatoïde longziekte*
			RA-ILD
			RA met longbetrokkenheid

Ziekte	***Onderliggende auto-immuunziekte***	***Categorie ILD***	***Onderliggende medische aandoening***
		ILD-SSc-ILD	*Systemische sclerose met longbetrokkenheid*
			Een ander ILD-fibrosing-sleutelwoord + een SSc sleutelwoord any SSc keyword op elk moment in de afgelopen 10 jaar
		ILD- Gemengde bindweefselziekte (MCTD)	*Gemengde bindweefselziekte*
			MCTD-ILD
		ILD-Andere diffuse CTD	*SLE*
			Juveniele dermatopolymyositis met longbetrokkenheid
			Andere dermatopolymyositis met longbetrokkenheid
			Dermatopolymyositis niet gespecificeerd met longbetrokkenheid
			Polymyositis met respiratoire betrokkenheid
			Sicca-syndroom met longbetrokkenheid
			Sjogren

Denmark

Sygdom	*Underliggende autoimmun sygdom*	*ILS Kategori*	*Underliggende sygdomstilstand*
ILS	*Ikke-Autoimmune*	*ILS-iNSIP*	*DJ841B Idiopa. lungefibrose m non-specific interstititial pneumonia*
		ILS-IPF	*DJ841C Idiopatisk lungefibrose UNS*
		ILS-Uklassificerbar interstitiel idiopatisk lungebetændelse	*DJ848 Anden interstitiel lungesygdom*
		ILS- Overfølsomhed Pneumonitis	*DJ67.x Pneumonitis f.a. hypersensitivitet overfor organisk støv*
		ILS- Eksponering-relaterede ILS	*DJ60 Støvlunge forårsaget af kulstøv*
			DJ61 Støvlunge forårsaget af asbest og andre mineralfibre
			DJ628 Anden form for silikose
			DJ63x Støvlunge forårsaget af andet uorganisk støv
			DJ649 Pneumokoniose UNS
			DJ660 Byssinose
		ILS-Sarkoidose	*DD860 Sarkoidose i lunger*
			DD862 Sarkoidose i både lunger og lymfeknuder
		ILS- Andet fibrosering	*DJ841 Anden interstitiel lungesygdom med fibrose*
			DJ849 Interstitiel lungesygdom UNS
		ILS- Andet (ikke-fibrotisk)	*DJ841D Akut interstitiel pneumoni*

**Sygdom**	**Underliggende autoimmun sygdom**	**ILS Kategori**	**Underliggende sygdomstilstand**
			DJ843 Bronchiolitis obliterans organiserende pneumoni (BOOP) or DJ841F
			DJ840A Proteinosis alveolaris pulmonum
			DJ841G Deskvamativ interstitiel pneumoni
			DD760 Histiocytose i de Langerhanske celler
	Autoimmune/ bindevævssygdomme -ILS	*ILS- Rheumatoid arthritis-associeret ILS*	*DM051 Reumatoid artritis med lungemanifestationer*
		ILS-SSc-ILS	*DM348 Anden form for systemisk sklerodermi + DJ84X for lung involvement*
			DM34x at any time within the last 10 years + any fibrotic code (DJ841/DJ848/DJ849)
		ILS- Blandet bindevævssygdom (+ DJ84X til lungeinddragelse)	*DM351 Andet blandingssyndrom ved generaliseret bindevævssygdom*
		ILS- Andre diffuse bindevævssygdomme	*DM321 Systemisk lupus erythematosus med organinvolvering*
			DM330 Dermatomyositis juvenilis
			DM331 Anden dermatomyositis
			DM339 Dermatopolymyositis UNS
			DM332 Polymyositis
			DM350 Sjögrens syndrom

Finland

Tauti	*Taustalla oleva autoimmuunisairaus*	*ILD-luokka*	*Lääketieteellisen tila: diagnoosi tai tekstilouhinta*
ILD: Interstitiaalinen keuhkosairaus	*Ei-autoimmuunisairaus*	*ILD-iNSIP*	*NSIP +J84.x*
		ILD-IPF	*J84.1 IPF/UIP, idiopaattinen keuhkofibroosi*
		ILD-Unclassifiable IIP	*NOT UIP/NSIP/COP/DIP/LIP/HP (NOT IPF)*
		ILD-Hypersensitivity Pneumonitis	*HP, hypersensitiviteetti pneumonia, allerginen alveoliitti, homepölykeuhko*
		ILD- Altistumiseen liittyvä interstitiaalinen keuhkosairaus	*J61, J67.0 (altiste tiedossa), J62.8*
		ILD- sarkoidoosi	*J86.0, D86.2*
		ILD- Muu fibroosi	*NSIP/COP/DIP + J84.9 or J84.8*
		ILD- Muu (ei-fibroottinen)	*NSIP/COP/DIP, ei IPF, ei UIP*
			COP, kryptogeeninen organisoituva pneumoia
			DIP

Tauti	*Taustalla oleva autoimmuunisairaus*	*ILD-luokka*	*Lääketieteellisen tila: diagnoosi tai tekstilouhinta*
			Deskvamatiivinen bronkioliitti
			RB-ILD
	Autoimmuunisairaus	*ILD- nivelreumaan liittyvän interstitiaalinen keuhkosairaus*	*M05.9, M06.0 + J99.0/J84.x/UIP/NSIP/fibroosi*
		ILD-SSc-ILD	*M34.x + J99.0/J84.x/UIP/NSIP/fibroosi*
		ILD- Sekamuotoiset sidekudossairaudet	*M35.8, M35.9 + J99.0/J84.x/UIP/NSIP/fibroosi*
		ILD-Muut diffuusi sidekudossairaudet	*M33.x/M34.xM35.0+J99.0 or J84.x/UIP/NSIP/fibroosi*

Greece

Nosos	*Ypokeimeni aftoanosi nosos*	*Katigoria diamesis pneumonopatheias*	*Ypokeimeni iatriki katastasi*
ILD	*Mi-aftoanosa*	*ILD-iNSIP*	*Idiopathis mi eidikí diámesi pnevmonia*
			NSIP
			NSIP inotikí
			Inotikí mi-eidikí diámesi pnevmonía
		ILD-IPF	*Idiopathis pnevmonikí ínosi*

Nosos	***Ypokeimeni aftoanosi nosos***	***Katigoria diamesis pneumonopatheias***	***Ypokeimeni iatriki katastasi***
			IPF
		ILD-Ataxinomiti idiopathis diamesi pnevmonia	*Idiopathis diámesi pnevmonía, mi prodiagrafómeni állos*
			Unclassifiable IIP
		ILD-Pnevmonítida yperevaisthisías	*Pnevmonítida yperevaisthisías*
			Pnevmonía yperevaisthisías
			Exogenoús allergikís kypselíditidas
		ILD-Diamesi pneumonopatheia schetizomeni me ekthesi	*Ergátes ánthraka*
			Amiántosi
			Pnevmoniokoníasi
			Vysinóosi
		ILD-Sarkoeídosi	*Sarkoeídosi tou pnévmona*
			Sarkoeídosi
		ILD-Alles inotikes	*Diámesi pnevmonopátheia*
			Diámeses pnevmonopátheies
			Pnevmonikí ínosi
			Inotikí diámesi pnevmonopátheia
			Inódis kypselíditida

Nosos	***Ypokeimeni aftoanosi nosos***	***Katigoria diamesis pneumonopatheias***	***Ypokeimeni iatriki katastasi***
			Diámesi pnevmonía
			Diámesi pnevmonítida
			Inódis pnévmona
			IIP
			Idiopathitis diámesi pnevmonía
			Inotikí diámesi pnevmonía
			ILD
			Idiopathis pnevmonikí nóso
			Pnevmonikí inotikí
			Perioristikí pnevmonikí nóso
			UIP
			Syníthis diámesi pnevmonía
			Diáchyti parenchymatikí pnevmonikí nóso
			Diámesi kypselíditida
		ILD-Alles (mi inotikes)	*Oxeía diámesi pnevmona*
			AIP
			Kryptogenis organoumeni pnevmonía

Nosos	***Ypokeimeni aftoanosi nosos***	***Katigoria diamesis pneumonopatheias***	***Ypokeimeni iatriki katastasi***
			Apofraktikí diámesi pnevmonía
			Istiokyttárosi kyttáron langerhans
			I istiokyttárosi tou langerhans
			Epifaneiodrastikés metalláxeis tou pnévmona
			Anapnefstikí vronchiolítida
	Aftoanosa/CTD-ILD	*ILD-Diamesi pneumonopatheia schetizomeni me revmatoeidi artritida*	*Revmatoeidís pnevmonopátheia*
			RA-ILD
		ILD-SSc-ILD	*Systimatikí sklírynsi me emplokí ton pnevmónon*
			Any ILD-other fibrosing keyword + any SSc keyword at any time within the last 10 years
		ILD-Mikti nosos tou syndetikou istou	*Mixed CTD-ILD diamesi pneumonopatheia apo mikti noso tou syndetikou istou*
			MCTD-ILD
		ILD-Alli diachyti nosos tou syndetikou istou	*SLE*
			Neanikí dermatopolymiozítida me emplokí ton pnevmónon
			Álli dermatopolyymosítida kai pnevmonikí emplokí

Nosos	*Ypokeimeni aftoanosi nosos*	*Katigoria diamesis pneumonopatheias*	*Ypokeimeni iatriki katastasi*
			Dermatopolymiozylítida mi kathorisméni me emplokí ton pnevmónon
			Polymyosítida me anapnefstikí anepárkeia
			Sýndromo Sicca me emplokí ton pnevmónon
			Sýndromo sjogren

Norway

Sykdom	*Underliggende autoimmun sykdom*	*ILD Kategori*	*Underliggende medisinsk tilstand*
ILD	*Ikke-Autoimmune*	*ILD-iNSIP*	*Idiopatisk non-spesifikk interstitiell pneumoni*
			NSIP
			Fibrotisk NSIP
			Fibrotisk ikke-spesifikk interstitial lungebetennelse
		ILD-IPF	*Idiopatisk lungefibrose*
			IPF
		ILD-Unclassifiable IIP	*Idiopatisk interstitial lungebetennelse, ikke spesifisert ellers*
			Unclassifiable IIP

Sykdom	***Underliggende autoimmun sykdom***	***ILD Kategori***	***Underliggende medisinsk tilstand***
		ILD- Overfølsomhet pneumonitt	*Overfølsomhet pneumonitt*
			Overfølsomhet lungebetennelse
			Ekstrinsisk allergisk alveolitis
		ILD- Eksponeringsrelatert interstitial lungesykdom	*Kullarbeidere / kullarbeidere pneumokoniose*
			Asbestose
			Pneumokoniose
			Byssinosis
		ILD- Sarcoidose	*Sarcoidose av lungen / Sarkoidose i lungene*
			Sarcoidose + any ILD-other fibrosing keyword
		ILD- Andre fibrosering	*Interstitiell lungesykdom / Interstitielle lungesykdommer*
			Lungfibrose
			Fibrotisk interstitiell lungesykdom
			Pulmonal fibrose
			Fibrosering alveolitis / fibrosing alveolitt
			Interstitial lungebetennelse
			Interstitial pneumonitt
			Fibrotisk lunge

Sykdom	***Underliggende autoimmun sykdom***	***ILD Kategori***	***Underliggende medisinsk tilstand***
			IIP
			Idiopatisk interstitial lungebetennelse
			Fibrotisk interstitial lungebetennelse
			ILD
			Idiopatisk lungesykdom
			Lungefibrotisk / pulmonal fibrotisk
			Restriktiv lungesykdom
			UIP
			Vanlig interstitial lungebetennelse
			Diffus parenkymal lungesykdom
			Interstitial alveolitis
		ILD- Annet (ikke fibrotisk)	*Akutt interstitial pneumonitt*
			AIP
			Kryptogen organisering lungebetennelse
			Desquamative interstitiell lungebetennelse
			Langerhans 'cell histiocytose
			Langerhans histiocytose

Sykdom	***Underliggende autoimmun sykdom***	***ILD Kategori***	***Underliggende medisinsk tilstand***
			Surfaktant mutasjoner av lungen
			Respiratorisk bronkiolit
	Autoimmune/ Bindevevssykdommer -ILD	*ILD- RA assosiert lungesykdom*	*Revmatoid lungesykdom*
			RA-ILD
			Reumatoid artritt med lungesamfunn
		ILD-SSc-ILD	*Systemisk sklerose med lungesamfunn*
			Any ILD-other fibrosing keyword + any SSc keyword at any time within the last 10 years
		ILD- Blandede bindevevssykdommer	*Mixed CTD-ILD*
			MCTD-ILD
		ILD-Andre diffuse bindevevssykdommer	*Systemisk lupus erythematosus SLE*
			Juvenil dermatopolymyositis med lungesamfunn
			Øvrig dermatopolymyositis med lungebetennelse
			Dermatopolymyositis unsp med lung involvering
			Polymyositis med respiratorisk involvering
			Sicca syndrom med lunge involvering
			Sjøgrens / sjogrens syndrom

Portugal

Doença	*Doença auto-imune subjacente*	*DPI Categoria*	*Condição médica subjacente*
DPI	*Não autoimune*	*DPI-PINEi*	*Pneumonia intersticial não específica idiopática*
			PINE<br>*NSIP*
			PINE fibrótica.<br>*NSIP*
			Pneumonia intersticial não específica fibrótica
		DPI-FPI	*Fibrose pulmonar idiopática*
			FPI
		DPI - Inclassificável PII	*Pneumonia intersticial idiopática, não especificada*
			Inclassificável PII
		DPI - Pneumonite de hipersensibilidade	*Pneumonite de hipersensibilidade*
			Pneumonia de hipersensibilidade
			Alveolite alérgica extrínseca
			Trabalhadores de Carvão
			Asbestose

Doença	*Doença auto-imune subjacente*	*DPI Categoria*	*Condição médica subjacente*
		DPI- Doença pulmonar intersticial relacionada à exposição	*Pneumoconiose*
			Bissinose
		DPI- Sarcoidose	*Sarcoidose do pulmão*
			Sarcoidose
		DPI-Other fibrosing	*Doença pulmonar intersticial*
			Doenças pulmonares intersticiais
			Fibrose pulmonar
			Doença pulmonar intersticial fibrótica
			Alveolite fibrosante
			Pneumonia intersticial
			Pneumonias intersticiais
			Pneumonite intersticial
			Pulmão fibrótico
			PII
			Pneumonia intersticial idiopática
			Pneumonia intersticial fibrótica

Doença	***Doença auto-imune subjacente***	***DPI Categoria***	***Condição médica subjacente***
			DPI
			Doença pulmonar idiopática
			Doença pulmonar restritiva
			PIU<br>*UIP*
			Pneumonia intersticial usual
			Doença pulmonar parenquimatosa difusa<br>*Doença difusa do parênquima pulmonar*
			Alveolite intersticial
		DPI-Other (non fibrotic)	*Pneumonite intersticial aguda*
			PIA<br>*AIP*
			Pneumonia organizativa criptogénica
			Pneumonia intersticial descamativa
			Histiocitose de células de Langerhans
			Histiocitose de Langerhans
			Mutações do surfactante do pulmão

Doença	***Doença auto-imune subjacente***	***DPI Categoria***	***Condição médica subjacente***
			Bronquiolite respiratória
	Autoimune/DPI- doença do tecido conjuntivo	*DPI - Artrite reumatóide com envolvimento pulmonar*	*Doença pulmonar reumatóide*
			AR-DPI
			Artrite reumatóide com envolvimento pulmonar
		DPI-SSc-ILD	*Esclerose sistémica com envolvimento pulmonar*<br>*Esclerodermia com envolvimento pulmonar*
			Outras DPI-fibróticas + esclerose sistémica nos últimos 10 anos
		DPI - Doença Mista do Tecido Conjuntivo	*DMTC-DIP*
		DPI-Outra doença do tecido conjuntivo difusa	*Lúpus eritematoso sistémico SLE*
			Dermatomiositis juvenil com envolvimento pulmonar
			Outras dermatomiosite com envolvimento pulmonary
			Dermatopomiosite não especificada com envolvimento pulmonar
			Polimiosite com envolvimento respiratório
			Síndrome Sicca com envolvimento pulmonar
			Síndrome de Sjögren

Section B. Methodology for calculating prevalence, incidence and relative percentages in Phase 1

Prevalence

In each participating centre, the annual crude prevalence was calculated as shown below. For the whole study period, it was the average of annual prevalences available in the period.

$$Prevalence = \frac{Number\ of\ prevalent\ cases}{Sum\ of\ persons\ at\ risk}$$

Prevalent cases (numerator) were those patients in the database ≥18 years presenting with at least one code and/or keyword for the conditions of interest between 01-January and 31-December of a specific year (including both inpatients and outpatients and excluding duplicates). Patients with codes and/or keywords for >1 condition within a given year (e.g. both systemic sclerosis-associated interstitial lung disease [SSc-ILD] and idiopathic pulmonary fibrosis [IPF]), were counted in each condition, but only once in overarching categories such as fibrosing interstitial lung diseases [F-ILDs]).
For each year, persons at risk were those living in the centre's area of influence (i.e. reference population) and who were alive and ≥18 years old at mid-year (i.e. 30-June). If only data from 01-January was available, the mean value between adjacent Januaries was used as a proxy for mid-year population.

Aside from this reference population, the participating centres receiving ILD cases from satellite centres (detailed in Table 1 in the main text) also had an extended (reference) population, formed by the population at risk in the centre's own area of influence plus the population at risk in the satellites' area of influence. When available, the main incidence calculations used this extended population as denominator, providing minimum estimates. However, as sensitivity analysis, maximum estimates were also obtained by using as denominator the reference population.

Both the reference and extended populations were provided by each centre. The total adult population for each country at each calendar year of the study period was obtained from the European Statistics Office (Eurostat) website.[1]

The prevalences obtained in the participating centres of each country were extrapolated to the whole country accounting for the relative size of the centres' population at risk with respect to the total adult population of the country.

Incidence

In each participating centre, the annual crude incidence was calculated as shown below. For the whole study period, it was the sum of annual incidences available in the period.

$$Incidence = \frac{Number\ of\ incident\ cases}{Sum\ of\ person - years\ at\ risk}$$

Incident cases (numerator) were those prevalent cases without any prior code and/or keyword for the same condition in the previous 10 years. As with prevalent cases, patients with codes and/or keywords for >1 condition within a given year (e.g. both SSc-ILD and IPF) were counted in each condition, but only once in overarching categories (e.g. F-ILDs).

The person-years at risk (denominator) were obtained considering that the follow-up for each individual present at mid-year (30-June) accounted for the whole year (i.e. 1 person-year for each person at risk in each calendar year).

As with prevalence, minimum and maximum estimates were obtained by using as denominator, respectively, the person-years at risk of the extended and reference populations; and the incidences obtained in the participating centres of each country were extrapolated to the whole country accounting for the relative size of the centres' population at risk with respect to the total adult population of the country.

Alternative methods and adjustments

Finland:

- The systematic search was performed in a database covering the entire population of the centre's region. Therefore, there was only one population at risk and a single incidence/prevalence estimate was obtained for each ILD.

Denmark:

- The 2017 data were incomplete due to a delay in the database update, and unavailable for 2018 due to database maintenance. As the number of cases had been quite constant in the previous years, and assuming the 2017 delay affected all ILDs equally, a correction factor of 1.28 (average decrease from 2016 to 2017 across subtypes) was applied to 2017 data. For 2018, the site calculated the expected numbers based on the former years back to 2010.

- The systematic search was performed in a database covering the entire country population (Danish National Patient Registry, DNPR), therefore, there was only one population at risk and a single incidence/prevalence estimate was obtained for each ILD. The exceptions were IPF, other F-ILDs and the overarching ILD categories containing them. Based on a yet unpublished review of clinical records,[2] and the review in Phase 2 of the present study, the Danish centre anticipated that a proportion of IPF cases would be recorded in DNPR with the same code as other F-ILDs (J849), probably because in many cases this general code was temporarily assigned at admission/referral but was not changed later, after the definitive diagnosis. According to the literature[2] approximately 28% of all ILDs (except sarcoidosis) are IPF in Denmark. Therefore, the number of IPF cases was adjusted upwards by subtracting the incident/prevalent cases of sarcoidosis to total incident/prevalent cases of ILD and multiplying by a factor of 0.28. Similarly, the number of other F-ILD cases was adjusted downwards, by subtracting the additional cases obtained for IPF (after adjusting) to the misclassified cases of other F-ILD. Misclassified and adjusted IPF cases were used as numerator to obtain minimum and maximum estimates, respectively; while misclassified and adjusted cases of other F-ILD were the numerator to obtain maximum and minimum estimates. As a result of these adjustments, overarching categories including IPF and other F-ILDs (non-IPF F-ILDs, F-ILDs and ILDs) also had minimum and maximum estimates in Denmark.
- As the searched database was nationwide, there was no need to extrapolate the incidence/prevalence to the whole country population.

Norway:

- The reference population primarily reported by Oslo University Hospital accounted for the Oslo municipality only. However, the hospital is a reference centre receiving 40% of ILD cases from the Oslo municipality (≈650,000 inhabitants), 40% from the rest of the South-East Health region (≈3,000,000 inhabitants; ≈2,350,000 excluding Oslo municipality) and 20% from the rest of Norway (≈5,300,000 inhabitants; ≈2,300,000 excluding the South-East region). Therefore, the population ratio for Oslo, the South-East region and whole Norway was 0.65/2.35/2.3 (i.e. 1/3.5/3.5), while the case ratio was 4/4/2 (i.e. 1/1/0.5). Assuming the centre caught 100% of cases in the Oslo municipality (i.e. that 4/0.65 was a valid relationship), the case ratio should had been

[2] Knarborg, et al. Idiopathic pulmonary fibrosis and other progressive fibrosing interstitial lung diseases in Denmark: clinical characteristics, treatment and economic consequences (PhD project, yet unpublished).

4/14/14. To achieve a case ratio of 1/1/0.5, the population ratio (denominator) was reduced to 0.65/0.65/0.32 thus obtaining a correction factor of 0.28 (0.65/2.35) for the South-East population and of 0.14 (0.32/2.3) for the Norwegian population. The reference population for each year of the study period was then calculated as follows: Oslo municipality + South-East region (excluding Oslo)*0.28 + Norway (excluding South-East region)*0.14. The whole South-East region population was used as extended population to obtain minimum estimates. Populations were obtained from the National Statistical Institute of Norway website.[3]

- ICD-10 codes used in the systematic search had only one decimal place, so cases of idiopathic non-specific interstitial pneumonia (iNSIP, coded J84.113), unclassifiable idiopathic interstitial pneumonias (uIIPs, coded J84.111) and other F-ILDs (coded J84.10) were merged.[3] To correct this misclassification, incident and prevalent cases of these three ILDs were estimated based on their relative percentage in the other Scandinavian countries.
- The incidence of non-F-ILDs, missing for the period between 2015-2018, was calculated for each year based on its prevalence and on the average incidence/prevalence ratio of the other ILDs in the country.

<u>Belgium</u>:

- The Liege University Hospital Centre was of reference for rheumatological ILDs, receiving patients not only from the centre's region (Wallonia), but also from the rest of the country and Luxembourg. To avoid overestimation, the incidence and prevalence of rheumatological ILDs were calculated using wider populations at risk than for other ILDs. The extended population encompassed the total adult population of Belgium and Luxembourg, while the reference population was limited to the adult population of the Wallonia region plus Luxembourg. The Wallonia region population was obtained from the Belgian Statistics Office[4] and the Luxemburg population from the country's Statistics Portal.[5]
- Ghent University Hospital, a reference centre for SSc-ILD, only provided data for this condition. Also, this centre could not provide its reference and extended populations. However, they reported that most patients managed at the department came from the

[3] The same occurred in Denmark and Finland. In Denmark, there were local codes in place which could be used in the systematic search to overcome this problem. In Finland, keywords could be used along with ICD-10 codes. In Norway, however, local codes and keywords could not be used.

regions of East and West Flanders. Therefore, the reference population considered was the sum of the total adult population for these regions (obtained from the Belgian Statistics Office[4]). This reference population was only used to obtain maximum incidence/prevalence estimates. Minimum estimates could not be obtained due to the lack of an extended population.

Greece:

- Only one of the four participating centres (University Hospital of Larissa) could capture most cases of rheumatological ILD.[4] The relative distribution of non-IPF F-ILD subtypes in Larissa was used to obtain a correction factor, which was applied to adjust upwards the number of non-IPF F-ILD cases in the three Greek centres with missing information to avoid underestimation. The correction factor was the inverse ratio of pulmonary and rheumatological cases of non-IPF F-ILD ([iNSIP + uIIPs + exposure-related ILDs + sarcoidosis + other F-ILDs + hypersensitivity pneumonitis (HP)] : [SSc-ILD + rheumatoid arthritis-associated ILD (RA-ILD) + mixed connective tissue disease (CTD)-ILD + other CTD-ILD]), and was multiplied by the total number of non-IPF F-ILD cases in each centre to obtain the number of rheumatological cases to be added (e.g. a ratio 2.5:1 would give a correction factor of 0.4. If the total number of non-IPF F-ILD cases was 71, then the final number after upwards adjustment would be 71+[71*0.4]=99).

Section C. Sample size calculations

The sample size for each study phase was calculated based on its primary objective. For Phase 1, the minimum population to obtain prevalence and incidence estimates with an acceptable precision was 100,000 persons, assuming most patients would have at least 1.5 years of follow-up. It was considered that, by including all subjects listed in each database from 01-January-2014 to 31-December-2018, this minimum sample would be exceeded. For Phase 2, assuming maximum uncertainty (p=q=50%) and a confidence of 95%, reviewing 100 prevalent cases per participating centre was calculated to offer a maximum margin of error (minimum precision) of 10% when describing the proportions of the primary variable.

[4] The pulmonary department in Beatriz Angelo Hospital (Portugal) was neither able to capture cases managed at other departments of the centre. However, this missing information was considered negligible, as cases managed at other departments were estimated to be less than 10%.

Section D. Exceptions to the general methodology for PPV calculation in Phase 2

Belgium:

- The only participating centre allowing positive predictive value (PPV) calculation (i.e. participating in both Phase 1 and 2) was the Ghent University Hospital. This centre, however, had two particularities: it was a reference centre for SSc-ILD and only provided data for this condition; and they did not conduct the systematic search, but retrieved cases directly from a departmental database which included all SSc-ILD patients presenting for assessment during each year. Thus, they theoretical PPV was 1.0. As using this PPV would have led to overestimation, a conservative approach was preferred instead, and the PPV used was the average for all countries (except Belgium). This PPV, however, was not used uniformly across Belgium. As the Liege University Hospital Centre was a reference centre for rheumatological ILDs, overestimation of RA-ILD, mixed CTD-ILD and other CTD-ILD incidence/prevalence was expected, and an alternative PPV was used. The centre selected a random sample of 100 patients from those listed in their database in 2018. This sample was searched manually for the main categories (ILDs, F-ILDs and non-IPF F-ILD), but also automatically by using a refined version of the study algorithm. This version retrieved only patients with at least three (instead of one) code and/or keyword for the condition of interest between 01-January and 31-December. A PPV of 59% was obtained, and the corresponding correction factor of 0.59 was applied to adjust incidence/prevalence of the rheumatological ILDs above.

Denmark:

- Due to the classification issues with IPF and other F-ILD detailed in Section A, in Phase 2 the consecutive non-IPF F-ILD cases used as source population were pre-reviewed in order to exclude misclassified ones. In total, 140 consecutive cases were reviewed to obtain a sample of 100 non-IPF F-ILD patients. Of the 40 excluded patients, two were considered not evaluable (one died before examination and one lacked data for the entire 2-year period of retrospective follow-up) and 38 were excluded (29 did not finally had an ILD, and 9 had IPF). This gave a PPV of 100/138 (73%, i.e. correction factor of 0.73).

Section E. Sensitivity analyses

Three sensitivity analyses were performed to address the uncertainties around estimates. First, in Phase 1, both minimum and maximum incidence/prevalence estimates were obtained to

account for potential under/overestimation arising from the population taken as reference for calculations (see Section B of this Supplementary Material). Second, in Phase 2, four additional methods were applied to obtain incidence/prevalence of progressive-fibrosing ILDs (PF-ILDs), to assess the impact of potentially centre-related biasing factors such as type (i.e. secondary or tertiary), receipt of referrals, or heterogeneity in referral/treatment patterns. These analyses used: (1b) The weighted country mean percentage of PF behaviour (as in the primary analysis) but applying a correction factor to the coverage areas of the sites participating in Phase 2 (i.e. which directly reported a higher/lower-than-mean percentage of PF behaviour); (2) An overall pooled percentage of PF behaviour across countries obtained through a random-effects model meta-analysis, thus not accounting for distribution of non-IPF F-ILDs within country (heterogeneity among countries was evaluated through the I^2-statistic); (2b) The overall pooled percentage of PF behaviour (as in Method 2), but applying a correction factor to the coverage area of sites participating in Phase 2 (as in Method 1b); (3) The arithmetic mean for percentages of PF behaviour from each country. Finally, to account for possible overestimations arising from the use of code and/or keywords in the systematic search, the country PPV was applied to crude incidence/prevalence estimates of Phase 1 and 2 to obtain adjusted estimates.

SUPPLEMENTARY FIGURES

sFigure 1. Algorithm for the systematic search in Phase 1

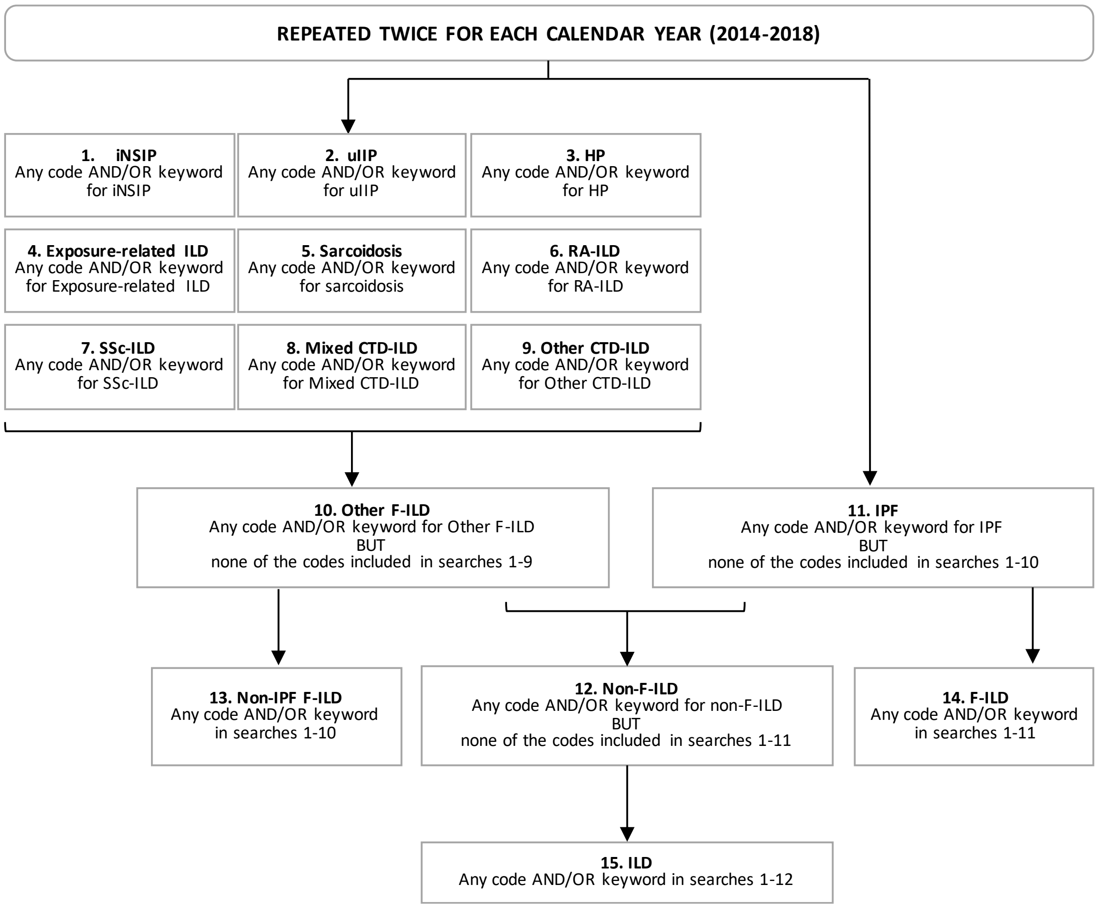

If a patient fulfilled the algorithm for more than one condition within a given year, the case was counted in each separate condition, but only once in overarching categories.
Abbreviations: CTD-ILD, connective tissue disease-associated interstitial lung disease; F-ILD, fibrosing interstitial lung disease; HP, hypersensitivity pneumonitis; ILD, interstitial lung disease; iNSIP, idiopathic non-specific interstitial pneumonia; IPF, idiopathic pulmonary fibrosis; RA-ILD; rheumatoid arthritis-associated interstitial lung disease; SSc-ILD, systemic sclerosis-associated interstitial lung disease; uIIP, idiopathic interstitial pneumonia; UIP, usual interstitial pneumonia.

sFigure 2. Annual prevalence (per 10^5 persons) of SSc-ILD in the participating countries during the study period (2014-2018)

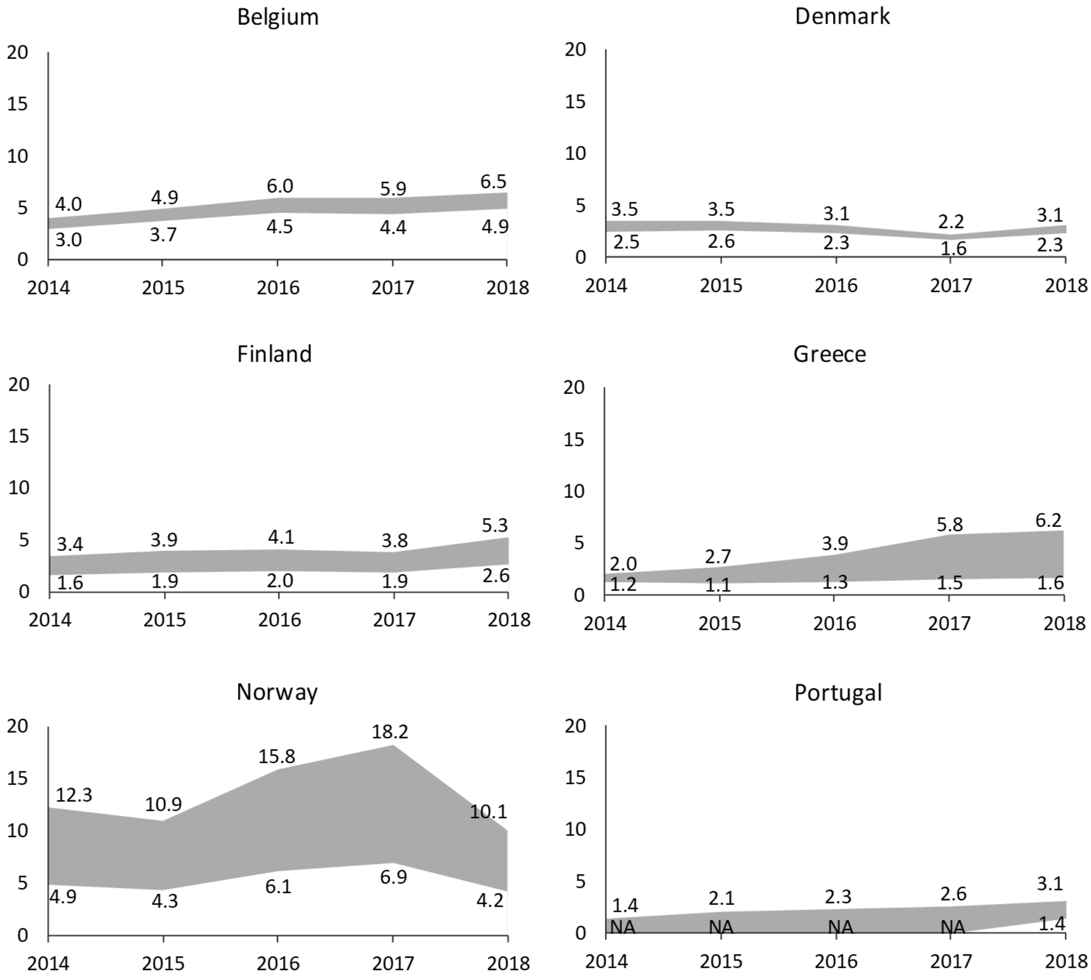

Areas show the widest variability observed in the primary plus sensitivity analyses. In Belgium, Greece, Norway and Portugal, this means the range between the minimum adjusted and the maximum crude estimates. In Denmark and Finland, there was only one participating centre which searched a national or regional database (respectively), so there were no reference and extended populations, but a single population (i.e. no maximum-minimum estimates, but a single estimate). In both countries, the area shows the range between the single crude and adjusted estimates. In Portugal, only one of the participating centres reported an extended population, and only for 2018. Therefore, minimum estimates could not be obtained for 2014-2017. For these years, the area shows the range from 0 to maximum crude estimates.

Abbreviations: SSc-ILD, systemic sclerosis-associated interstitial lung disease; NA, not available.

SUPPLEMENTARY TABLES

sTable 1. PPVs by centre and country

Country	Centre 1	Centre 2	Centre 3	Overall
Belgium	0.75			0.75
Denmark	0.73			0.73
Finland	0.49			0.49
Greece	0.64	0.93	0.80	0.79
Norway	0.75			0.75
Portugal	0.68	0.77		0.73

Belgium: Centre 1 - Ghent University Hospital.
Denmark: Centre 1 (Lillebælt Hospital).
Finland: Centre 1 (Turku University Hospital)
Greece: Centre 1 (University Hospital of Larissa), Centre 2 (General Hospital of Thessaloniki), Centre 3 (Athens Medical Centre).
Norway: Centre 1 (Oslo University Hospital, Rikshospitalet).
Portugal: Centre 1 (Sao Joao University Hospital Centre), Centre 2 (Beatriz Angelo Hospital).
Abbreviations: PPV, positive predictive value.

sTable 2. Incidence per 10^5 person-years (95%CI) of ILDs, F-ILDs, IPF, non-IPF F-ILDs, and SSc-ILD in each country and overall, annually and for the whole study period

	Country	Estimate	Study period	2014	2015	2016	2017	2018
**ILDs**	Belgium	Min	17.9 (16.9,18.9)	14.0 (12.3,16.0)	17.5 (15.4,19.9)	17.2 (15.1,19.5)	21.0 (18.7,23.7)	20.5 (18.1,23.1)
		Max	71.1 (67.7,74.7)	55.3 (49.3,62.1)	63.5 (56.8,71.1)	71.4 (63.9,79.7)	86.9 (78.4,96.3)	83.6 (75.2,93.0)
	Denmark	Min	41.3 (40.5,42.1)	41.0 (39.1,42.9)	43.8 (41.9,45.8)	43.1 (41.2,45.0)	38.5 (36.8,40.4)	40.1 (38.3,42.0)
		Max	56.9 (56.0,57.9)	56.5 (54.3,58.8)	60.4 (58.2,62.8)	59.4 (57.2,61.7)	53.1 (51.0,55.3)	55.3 (53.2,57.5)
	Finland	Min	26.2 (24.1,28.6)	36.7 (31.2,43.3)	20.8 (16.7,25.9)	22.6 (18.3,27.8)	24.8 (20.3,30.2)	26.3 (21.7,31.9)
		Max	53.5 (50.4,56.9)	75.0 (66.8,84.1)	42.4 (36.4,49.4)	46.1 (39.8,53.3)	50.6 (44.0,58.2)	53.7 (46.9,61.4)
	Greece	Min	10.6 (10.1,11.1)	5.8 (5.1,6.6)	10.0 (9.0,11.1)	11.4 (10.3,12.6)	13.3 (12.1,14.5)	12.5 (11.4,13.7)
		Max	24.1 (23.2,25.1)	20.2 (18.1,22.5)	21.7 (19.8,23.8)	23.2 (21.3,25.4)	29.2 (27.0,31.7)	25.2 (23.2,27.5)
	Norway	Min	12.4 (11.8,13.1)	NA	12.3 (11.0,13.7)	16.6 (15.2,18.2)	11.1 (10.0,12.4)	9.9 (8.8,11.1)
		Max	31.8 (30.4,33.2)	NA	31.4 (28.6,34.5)	43.2 (39.9,46.7)	29.4 (26.7,32.3)	24.2 (21.9,26.7)
	Portugal	Min	8.0 (7.6,8.5)	6.3 (5.3,7.3)	8.2 (7.1,9.4)	7.3 (6.3,8.4)	8.7 (7.6,9.9)	9.4 (8.3,10.5)
		Max	11.1 (10.5,11.7)	8.6 (7.5,9.9)	11.3 (10.1,12.7)	10.1 (8.9,11.4)	11.9 (10.7,13.4)	12.9 (11.7,14.2)
	Overall	Min	20.1 (18.3,21.8)	22.3 (20.5,24.2)	18.7 (17.0,20.4)	19.6 (17.9,21.3)	19.7 (17.9,21.4)	20.0 (18.2,21.8)
		Max	42.0 (39.1,44.9)	43.1 (40.1,46.1)	38.5 (35.7,41.3)	42.2 (39.3,45.1)	43.5 (40.6,46.5)	42.5 (39.7,45.3)
**F-ILDs**	Belgium	Min	16.7 (15.8,17.7)	13.3 (11.6,15.2)	16.4 (14.4,18.7)	16.0 (14.0,18.2)	19.9 (17.6,22.5)	18.7 (16.5,21.2)
		Max	66.4 (63.1,69.8)	52.3 (46.5,58.8)	59.5 (53.0,66.7)	66.3 (59.2,74.3)	82.2 (74.1,91.3)	76.2 (68.3,85.1)
	Denmark	Min	40.2 (39.4,41.1)	40.0 (38.2,41.9)	42.8 (40.9,44.8)	42.0 (40.1,43.9)	37.5 (35.7,39.3)	39.1 (37.3,40.9)
		Max	55.5 (54.5,56.5)	55.1 (53.0,57.4)	59.0 (56.8,61.3)	57.9 (55.7,60.1)	51.7 (49.6,53.8)	53.9 (51.8,56.0)
	Finland	Min	22.4 (20.4,24.6)	33.1 (27.8,39.3)	18.3 (14.5,23.1)	18.2 (14.4,23.0)	21.3 (17.2,26.4)	21.5 (17.4,26.6)
		Max	45.8 (42.9,48.9)	67.5 (59.8,76.2)	37.3 (31.7,43.9)	37.1 (31.6,43.7)	43.5 (37.4,50.5)	43.8 (37.8,50.9)
		Min	9.8 (9.4,10.3)	5.3 (4.6,6.1)	9.4 (8.5,10.5)	10.6 (9.6,11.7)	12.3 (11.2,13.5)	11.5 (10.4,12.7)

	Country	Estimate	Study period	2014	2015	2016	2017	2018
	Greece	Max	22.4 (21.5,23.4)	18.9 (16.9,21.1)	20.4 (18.6,22.5)	21.6 (19.7,23.7)	27.2 (25.1,29.6)	23.1 (21.1,25.2)
	Norway	Min	11.2 (10.6,11.8)	NA	11.0 (9.8,12.4)	14.5 (13.2,16.0)	10.1 (9.0,11.4)	9.1 (8.1,10.2)
		Max	28.5 (27.2,29.9)	NA	28.2 (25.6,31.1)	37.7 (34.7,41.0)	26.8 (24.3,29.6)	22.2 (20.1,24.6)
	Portugal	Min	6.3 (5.8,6.7)	4.6 (3.9,5.6)	6.0 (5.1,7.1)	5.4 (4.5,6.4)	7.2 (6.2,8.3)	7.7 (6.8,8.7)
		Max	8.6 (8.1,9.2)	6.4 (5.5,7.5)	8.3 (7.2,9.5)	7.4 (6.4,8.6)	9.9 (8.7,11.2)	10.6 (9.5,11.8)
	Overall	Min	18.3 (16.6,19.9)	20.6 (18.8,22.4)	17.2 (15.5,18.8)	17.5 (15.9,19.2)	18.0 (16.4,19.7)	17.9 (16.2,19.6)
		Max	38.4 (35.6,41.2)	40.0 (37.2,42.9)	35.5 (32.8,38.2)	38.0 (35.2,40.8)	40.2 (37.4,43.1)	38.3 (35.6,41.0)
**IPF**	Belgium	Min	0.9 (0.7,1.3)	0.7 (0.3,1.5)	1.1 (0.6,2.1)	0.6 (0.3,1.4)	1.2 (0.7,2.2)	1.1 (0.6,2.1)
		Max	3.8 (2.9,4.9)	2.7 (1.4,5.5)	4.1 (2.4,7.0)	2.5 (1.2,5.3)	5.0 (3.0,8.4)	4.6 (2.7,7.9)
	Denmark	Min	0.4 (0.4,0.5)	0.4 (0.4,0.5)	0.5 (0.5,0.6)	0.4 (0.4,0.5)	0.4 (0.3,0.4)	0.4 (0.4,0.5)
		Max	10.6 (10.2,11.1)	10.3 (9.4,11.3)	11.4 (10.4,12.4)	11.4 (10.5,12.4)	9.8 (8.9,10.7)	10.3 (9.4,11.2)
	Finland	Min	3.5 (2.7,4.4)	4.2 (2.6,6.8)	3.5 (2.1,6.0)	3.0 (1.7,5.3)	3.5 (2.1,5.9)	3.2 (1.9,5.6)
		Max	7.1 (6.0,8.4)	8.5 (6.1,12.0)	7.2 (5.0,10.4)	6.1 (4.1,9.2)	7.1 (4.9,10.3)	6.6 (4.5,9.7)
	Greece	Min	3.5 (3.3,3.8)	1.5 (1.2,2.0)	3.5 (3.0,4.2)	3.8 (3.2,4.5)	4.3 (3.6,5.0)	4.5 (3.9,5.3)
		Max	8.0 (7.4,8.5)	4.3 (3.4,5.4)	7.6 (6.5,8.9)	8.2 (7.0,9.5)	9.2 (8.0,10.6)	9.7 (8.5,11.2)
	Norway	Min	2.2 (1.9,2.5)	NA	2.1 (1.6,2.7)	2.4 (1.9,3.0)	2.2 (1.7,2.8)	2.0 (1.6,2.6)
		Max	5.5 (5.0,6.2)	NA	5.3 (4.2,6.6)	6.2 (5.0,7.6)	5.8 (4.7,7.2)	4.9 (4.0,6.2)
	Portugal	Min	0.9 (0.7,1.1)	0.7 (0.4,1.1)	0.7 (0.4,1.1)	0.6 (0.4,1.0)	1.2 (0.9,1.8)	1.1 (0.8,1.5)
		Max	1.2 (1.0,1.4)	1.0 (0.7,1.5)	0.9 (0.6,1.4)	0.9 (0.6,1.3)	1.7 (1.3,2.3)	1.5 (1.1,2.0)
	Overall	Min	2.0 (1.4,2.5)	1.7 (1.2,2.3)	2.0 (1.4,2.5)	1.8 (1.3,2.4)	2.2 (1.6,2.7)	2.1 (1.5,2.7)
		Max	6.0 (4.9,7.1)	5.4 (4.3,6.4)	6.1 (4.9,7.2)	5.9 (4.8,7.0)	6.4 (5.3,7.6)	6.3 (5.2,7.4)
	Belgium	Min	15.8 (14.9,16.7)	12.6 (11.0,14.4)	15.3 (13.3,17.5)	15.4 (13.5,17.5)	18.7 (16.6,21.2)	17.6 (15.4,20.0)
		Max	62.6 (59.5,65.9)	49.5 (43.9,55.8)	55.4 (49.3,62.3)	63.8 (56.9,71.6)	77.2 (69.4,86.0)	71.7 (64.1,80.1)

	Country	Estimate	Study period	2014	2015	2016	2017	2018
**Non-IPF F-ILDs**	Denmark	Min	32.5 (31.8,33.3)	32.5 (30.8,34.2)	34.6 (32.9,36.3)	33.7 (32.0,35.4)	30.4 (28.8,32.0)	31.6 (30.0,33.3)
		Max	54.9 (53.9,55.9)	54.6 (52.4,56.8)	58.3 (56.1,60.6)	57.3 (55.1,59.6)	51.1 (49.1,53.3)	53.3 (51.2,55.4)
	Finland	Min	19.0 (17.1,21.0)	28.9 (24.0,34.8)	14.7 (11.4,19.1)	15.2 (11.8,19.6)	17.8 (14.1,22.5)	18.2 (14.5,23.0)
		Max	38.7 (36.0,41.5)	58.9 (51.8,67.1)	30.1 (25.1,36.1)	31.0 (25.9,37.0)	36.4 (30.9,42.8)	37.2 (31.7,43.8)
	Greece	Min	6.3 (5.9,6.7)	3.8 (3.2,4.5)	5.9 (5.1,6.7)	6.8 (6.0,7.7)	8.0 (7.2,9.0)	6.9 (6.1,7.9)
		Max	15.5 (14.8,16.3)	15.2 (13.4,17.2)	13.5 (12.0,15.2)	14.4 (12.9,16.1)	19.6 (17.8,21.6)	14.8 (13.3,16.6)
	Norway	Min	9.0 (8.5,9.6)	NA	9.0 (7.9,10.2)	12.1 (10.9,13.5)	7.9 (7.0,9.0)	7.1 (6.2,8.1)
		Max	23.0 (21.8,24.2)	NA	22.9 (20.6,25.6)	31.5 (28.8,34.6)	21.0 (18.8,23.5)	17.3 (15.4,19.4)
	Portugal	Min	5.4 (5.0,5.8)	3.9 (3.2,4.8)	5.3 (4.5,6.3)	4.7 (4.0,5.7)	5.9 (5.0,7.0)	6.6 (5.7,7.6)
		Max	7.4 (7.0,7.9)	5.4 (4.6,6.4)	7.4 (6.4,8.5)	6.5 (5.6,7.6)	8.2 (7.1,9.4)	9.1 (8.1,10.2)
	Overall	Min	15.1 (13.6,16.7)	17.6 (15.9,19.2)	14.0 (12.5,15.5)	14.5 (13.0,16.0)	14.8 (13.3,16.3)	14.7 (13.2,16.3)
		Max	34.3 (31.7,36.9)	36.7 (34.0,39.5)	31.3 (28.8,33.8)	34.1 (31.5,36.7)	35.6 (32.9,38.3)	33.9 (31.4,36.4)
**SSc-ILD**	Belgium	Min	0.9 (0.7,1.0)	0.4 (0.2,0.8)	1.1 (0.8,1.6)	1.1 (0.8,1.6)	0.7 (0.4,1.1)	1.0 (0.6,1.4)
		Max	1.1 (1.0,1.4)	0.5 (0.3,0.9)	1.5 (1.1,2.0)	1.5 (1.1,2.1)	1.0 (0.6,1.4)	1.3 (0.9,1.8)
	Denmark	Min	0.5 (0.4,0.6)	0.5 (0.3,0.7)	0.5 (0.3,0.7)	0.4 (0.3,0.7)	0.5 (0.3,0.7)	0.5 (0.3,0.7)
		Max	0.6 (0.5,0.7)	0.7 (0.5,1.0)	0.6 (0.4,0.9)	0.6 (0.4,0.8)	0.6 (0.4,0.9)	0.6 (0.4,0.9)
	Finland	Min	0.3 (0.1,0.7)	0.4 (0.1,1.9)	0.1 (0.0,2.1)	0.4 (0.1,1.9)	0.2 (0.0,1.8)	0.5 (0.1,2.0)
		Max	0.7 (0.4,1.1)	0.8 (0.3,2.4)	0.3 (0.0,1.8)	0.8 (0.2,2.4)	0.5 (0.1,2.0)	1.0 (0.4,2.7)
	Greece	Min	0.4 (0.3,0.5)	0.4 (0.2,0.6)	0.4 (0.2,0.6)	0.4 (0.2,0.6)	0.5 (0.3,0.8)	0.4 (0.3,0.7)
		Max	1.1 (0.9,1.4)	1.4 (0.9,2.1)	1.0 (0.6,1.5)	0.7 (0.4,1.2)	1.7 (1.2,2.3)	1.0 (0.7,1.5)
	Norway	Min	0.9 (0.7,1.1)	NA	0.7 (0.4,1.1)	1.5 (1.1,2.0)	0.7 (0.5,1.1)	0.6 (0.4,1.0)
		Max	2.2 (1.9,2.6)	NA	1.8 (1.2,2.6)	3.8 (2.9,5.0)	1.9 (1.3,2.8)	1.5 (1.0,2.2)
		Min	0.3 (0.3,0.5)	0.3 (0.1,0.6)	0.5 (0.3,0.9)	0.3 (0.1,0.6)	0.3 (0.1,0.6)	0.3 (0.2,0.6)

Country	Estimate	Study period	2014	2015	2016	2017	2018
Portugal	Max	0.5 (0.4,0.6)	0.4 (0.2,0.7)	0.7 (0.5,1.2)	0.4 (0.2,0.8)	0.4 (0.2,0.8)	0.5 (0.3,0.8)
Overall	Min	0.5 (0.2,0.8)	0.4 (0.1,0.6)	0.5 (0.2,0.8)	0.6 (0.3,0.9)	0.5 (0.2,0.7)	0.5 (0.2,0.8)
	Max	1.0 (0.6,1.4)	0.7 (0.4,1.1)	1.0 (0.6,1.4)	1.3 (0.8,1.8)	1.0 (0.6,1.4)	1.0 (0.6,1.4)

The table shows the widest variability observed. In Belgium, Greece and Norway, this means the minimum adjusted and the maximum crude estimates (the participating centre in Norway could not retrieve incident cases for 2014, and thus incidence could not be estimated for that year). In Denmark and Finland, there was only one participating centre which searched a national or regional database (respectively), so there were no reference and extended populations, but a single population (i.e. no maximum-minimum estimates, but a single estimate). In these countries, the single crude and adjusted estimates are shown. In Portugal, only one of the participating centres reported an extended population, and only for 2018. Plus, incident cases for 2018 could not be retrieved in this centre, so minimum adjusted estimates could not be obtained for Portugal. Instead, the table shows the nearest estimates (maximum adjusted) as minimum values.

Abbreviations: CI, confidence interval; ILDs, interstitial lung diseases; F-ILDs, fibrosing interstitial lung diseases; IPF, idiopathic pulmonary fibrosis; NA, not available; SSc-ILD, systemic sclerosis-associated interstitial lung disease.

sTable 3. Prevalence per 10^5 persons (95%CI) of ILD, F-ILD, IPF, non-IPF F-ILD, and SSc-ILD in each country and overall, annually and for the whole study period

	Country	Estimate	Study period	2014	2015	2016	2017	2018
**ILDs**	Belgium	Min	49.8 (46.7,53.0)	37.5 (34.9,40.1)	45.5 (42.5,48.5)	52.0 (48.8,55.3)	55.4 (52.0,58.9)	61.4 (57.8,65.1)
		Max	195.1 (184.3,205.8)	145.4 (136.7,154.2)	163.3 (154.1,172.6)	213.1 (201.5,224.6)	225.4 (213.4,237.5)	247.4 (234.6,260.1)
	Denmark	Min	132.2 (128.9,135.6)	130.8 (127.4,134.2)	141.1 (137.6,144.6)	128.5 (125.2,131.8)	121.7 (118.5,124.9)	139.2 (135.7,142.6)
		Max	182.4 (178.5,186.3)	180.4 (176.5,184.4)	194.7 (190.6,198.7)	177.2 (173.3,181.1)	167.9 (164.1,171.6)	191.9 (187.9,195.9)
	Finland	Min	90.9 (81.4,100.3)	90.8 (81.3,100.3)	85.8 (76.6,95.0)	87.8 (78.5,97.1)	90.7 (81.3,100.1)	99.0 (89.2,108.8)
		Max	185.4 (172.0,198.9)	185.3 (171.8,198.9)	175.1 (162.0,188.3)	179.2 (166.0,192.5)	185.2 (171.7,198.6)	202.1 (188.1,216.1)
	Greece	Min	23.8 (21.0,26.6)	16.3 (14.0,18.6)	17.5 (15.1,19.8)	23.0 (20.3,25.8)	28.5 (25.4,31.5)	33.6 (30.3,36.9)
		Max	93.3 (85.0,101.5)	67.7 (60.7,74.7)	67.4 (60.4,74.4)	90.3 (82.2,98.4)	113.0 (103.9,122.1)	128.3 (118.6,138.0)
	Norway	Min	55.8 (53.0,58.5)	48.2 (45.5,50.8)	49.5 (46.8,52.1)	59.9 (57.0,62.7)	63.4 (60.5,66.3)	56.8 (54.1,59.5)
		Max	142.1 (136.0,148.2)	121.1 (115.3,126.9)	126.5 (120.6,132.4)	155.6 (149.1,162.0)	167.4 (160.8,174.0)	138.8 (133.1,144.5)
	Portugal	Min	31.3 (28.1,34.4)	NA	NA	NA	NA	35.4 (32.1,38.8)
		Max	52.4 (47.9,56.9)	35.9 (32.2,39.5)	45.9 (41.8,49.9)	51.8 (47.5,56.2)	58.0 (53.4,62.6)	76.7 (70.6,82.9)
	Overall	Min	65.8 (62.3,69.4)	60.1 (56.7,63.6)	63.3 (59.8,66.8)	65.8 (62.2,69.3)	67.9 (64.3,71.5)	72.1 (68.4,75.8)
		Max	142.6 (136.4,148.8)	122.6 (116.9,128.4)	128.8 (122.9,134.7)	144.5 (138.3,150.8)	152.8 (146.4,159.2)	164.2 (157.5,170.9)
**F-ILDs**	Belgium	Min	48.3 (45.2,51.4)	36.6 (34.1,39.2)	44.3 (41.4,47.2)	50.3 (47.2,53.5)	53.9 (50.5,57.2)	58.9 (55.3,62.4)
		Max	189.0 (178.5,199.5)	142.0 (133.4,150.6)	159.0 (149.9,168.0)	205.9 (194.6,217.1)	219.0 (207.2,230.8)	236.8 (224.5,249.1)
	Denmark	Min	129.7 (126.3,133.0)	128.1 (124.8,131.4)	138.6 (135.2,142.1)	125.9 (122.7,129.2)	119.2 (116.0,122.3)	136.6 (133.2,139.9)
		Max	178.8 (175.0,182.7)	176.7 (172.8,180.6)	191.2 (187.2,195.3)	173.7 (169.9,177.5)	164.4 (160.7,168.1)	188.3 (184.4,192.3)
	Finland	Min	83.0 (73.9,92.0)	84.3 (75.2,93.5)	79.9 (71.0,88.8)	79.3 (70.5,88.1)	82.3 (73.3,91.2)	89.0 (79.7,98.3)
		Max	169.3 (156.4,182.2)	172.1 (159.1,185.2)	163.1 (150.4,175.7)	161.8 (149.2,174.4)	167.9 (155.1,180.7)	181.6 (168.3,194.9)
		Min	22.7 (20.0,25.4)	15.7 (13.4,17.9)	16.8 (14.4,19.1)	22.1 (19.4,24.8)	27.1 (24.1,30.0)	31.7 (28.5,35.0)

	Country	Estimate	Study period	2014	2015	2016	2017	2018
	Greece	Max	89.5 (81.4,97.6)	65.5 (58.6,72.3)	65.2 (58.3,72.1)	87.2 (79.2,95.2)	108.3 (99.4,117.2)	121.6 (112.1,131.0)
	Norway	Min	50.9 (48.3,53.5)	44.0 (41.4,46.5)	44.8 (42.2,47.3)	53.1 (50.4,55.8)	58.6 (55.8,61.4)	52.9 (50.2,55.5)
		Max	129.7 (123.9,135.5)	110.6 (105.1,116.2)	114.4 (108.8,120.0)	138.0 (131.9,144.1)	154.8 (148.5,161.2)	129.2 (123.7,134.7)
	Portugal	Min	20.3 (17.8,22.8)	NA	NA	NA	NA	26.7 (23.8,29.6)
		Max	34.0 (30.4,37.6)	21.0 (18.2,23.7)	28.4 (25.1,31.6)	32.2 (28.8,35.6)	36.9 (33.2,40.5)	57.9 (52.5,63.2)
	Overall	Min	60.6 (57.2,64.0)	55.7 (52.4,59.0)	58.6 (55.2,62.0)	59.8 (56.4,63.2)	62.0 (58.6,65.5)	66.8 (63.2,70.4)
		Max	132.5 (126.5,138.5)	114.6 (109.1,120.2)	120.2 (114.5,125.9)	133.1 (127.2,139.1)	141.9 (135.7,148.1)	152.6 (146.1,159.0)
**IPF**	Belgium	Min	4.4 (3.0,5.8)	0.9 (0.3,1.6)	2.9 (1.8,4.0)	4.9 (3.5,6.4)	5.8 (4.2,7.4)	7.5 (5.7,9.3)
		Max	17.8 (12.9,22.6)	3.8 (1.5,6.0)	10.6 (7.0,14.2)	20.9 (15.5,26.2)	24.2 (18.5,30.0)	31.0 (24.5,37.4)
	Denmark	Min	2.7 (2.2,3.1)	2.6 (2.2,3.1)	2.8 (2.3,3.3)	2.7 (2.2,3.2)	2.3 (1.9,2.7)	2.8 (2.4,3.3)
		Max	27.7 (26.2,29.3)	25.9 (24.4,27.4)	29.3 (27.8,30.9)	27.4 (25.9,28.9)	25.7 (24.2,27.1)	30.4 (28.8,32.0)
	Finland	Min	11.2 (7.8,14.5)	8.5 (5.6,11.4)	10.5 (7.2,13.7)	11.3 (8.0,14.6)	12.5 (9.0,16.0)	13.0 (9.5,16.6)
		Max	22.8 (18.0,27.5)	17.3 (13.2,21.5)	21.3 (16.8,25.9)	23.0 (18.3,27.8)	25.4 (20.4,30.4)	26.6 (21.5,31.7)
	Greece	Min	6.2 (4.8,7.7)	2.9 (1.9,3.9)	4.1 (2.9,5.2)	5.9 (4.5,7.3)	8.0 (6.4,9.6)	10.3 (8.4,12.1)
		Max	17.8 (14.2,21.4)	8.2 (5.8,10.7)	11.7 (8.8,14.6)	16.9 (13.4,20.5)	23.0 (18.9,27.1)	29.3 (24.7,33.9)
	Norway	Min	8.1 (7.1,9.2)	8.1 (7.0,9.2)	6.5 (5.5,7.4)	8.1 (7.0,9.1)	8.7 (7.6,9.8)	9.0 (7.9,10.1)
		Max	20.7 (18.3,23.0)	20.5 (18.1,22.8)	16.5 (14.4,18.7)	21.0 (18.6,23.4)	22.9 (20.5,25.4)	22.0 (19.7,24.3)
	Portugal	Min	2.8 (1.9,3.7)	NA	NA	NA	NA	3.1 (2.1,4.1)
		Max	4.7 (3.4,6.0)	2.8 (1.8,3.8)	4.1 (2.9,5.3)	4.9 (3.6,6.3)	5.5 (4.1,6.9)	6.8 (4.9,8.6)
	Overall	Min	6.2 (5.1,7.3)	4.4 (3.4,5.4)	5.3 (4.2,6.3)	6.3 (5.2,7.5)	7.1 (5.9,8.4)	7.8 (6.6,9.1)
		Max	18.6 (16.3,20.9)	13.1 (11.1,15.0)	15.6 (13.5,17.7)	19.0 (16.7,21.3)	21.1 (18.7,23.6)	24.3 (21.7,27.0)
	Belgium	Min	44.2 (41.3,47.0)	35.7 (33.2,38.2)	41.4 (38.7,44.2)	45.4 (42.5,48.3)	48.1 (45.0,51.2)	51.4 (48.2,54.6)
		Max	172.4 (162.6,182.1)	138.2 (129.8,146.6)	148.4 (139.8,157.0)	185.0 (174.6,195.3)	194.8 (184.0,205.6)	205.8 (194.8,216.9)

	Country	Estimate	Study period	2014	2015	2016	2017	2018
**Non-IPF F-ILDs**	Denmark	Min	109.6 (106.6,112.7)	109.3 (106.2,112.4)	117.7 (114.5,120.9)	106.3 (103.3,109.3)	100.3 (97.4,103.3)	114.7 (111.6,117.8)
		Max	175.3 (171.5,179.2)	173.0 (169.1,176.9)	187.8 (183.7,191.8)	170.2 (166.4,174.0)	160.9 (157.2,164.6)	184.8 (180.8,188.7)
	Finland	Min	71.8 (63.4,80.2)	75.9 (67.2,84.5)	69.4 (61.2,77.7)	68.0 (59.8,76.2)	69.8 (61.5,78.0)	75.9 (67.4,84.5)
		Max	146.5 (134.6,158.5)	154.8 (142.4,167.2)	141.7 (129.9,153.5)	138.8 (127.1,150.5)	142.4 (130.6,154.2)	155.0 (142.7,167.3)
	Greece	Min	17.2 (14.9,19.6)	13.7 (11.6,15.8)	13.4 (11.3,15.5)	16.9 (14.6,19.3)	19.8 (17.3,22.4)	22.3 (19.6,25.0)
		Max	69.0 (61.9,76.1)	55.5 (49.2,61.8)	51.9 (45.7,58.0)	67.8 (60.8,74.9)	82.1 (74.3,89.8)	88.1 (80.0,96.1)
	Norway	Min	42.8 (40.4,45.2)	35.9 (33.6,38.2)	38.3 (35.9,40.6)	45.0 (42.5,47.5)	49.9 (47.4,52.5)	43.9 (41.5,46.2)
		Max	109.0 (103.7,114.4)	90.2 (85.1,95.2)	97.9 (92.7,103.0)	117.0 (111.4,122.6)	131.9 (126.0,137.8)	107.2 (102.2,112.2)
	Portugal	Min	17.5 (15.1,19.8)	NA	NA	NA	NA	23.6 (20.8,26.3)
		Max	29.3 (25.9,32.6)	18.2 (15.6,20.8)	24.3 (21.3,27.3)	27.3 (24.1,30.4)	31.4 (28.0,34.7)	51.1 (46.1,56.1)
	Overall	Min	51.8 (48.7,55.0)	48.9 (45.9,52.0)	50.6 (47.5,53.8)	51.0 (47.8,54.1)	52.5 (49.3,55.6)	56.2 (52.9,59.4)
		Max	117.4 (111.8,123.1)	105.0 (99.7,110.3)	108.6 (103.3,114.0)	117.7 (112.1,123.3)	123.9 (118.2,129.7)	132.0 (126.0,137.9)
**SSc-ILD**	Belgium	Min	4.1 (3.0,5.3)	3.0 (2.0,4.0)	3.7 (2.6,4.8)	4.5 (3.3,5.7)	4.4 (3.3,5.6)	4.9 (3.7,6.2)
		Max	5.5 (4.2,6.8)	4.0 (2.9,5.1)	4.9 (3.7,6.2)	6.0 (4.6,7.4)	5.9 (4.5,7.3)	6.5 (5.1,8.0)
	Denmark	Min	2.2 (1.8,2.7)	2.5 (2.1,3.0)	2.6 (2.1,3.0)	2.3 (1.8,2.7)	1.6 (1.3,2.0)	2.3 (1.8,2.7)
		Max	3.1 (2.6,3.6)	3.5 (2.9,4.0)	3.5 (3.0,4.1)	3.1 (2.6,3.6)	2.2 (1.8,2.7)	3.1 (2.6,3.6)
	Finland	Min	2.0 (0.6,3.4)	1.6 (0.4,2.9)	1.9 (0.5,3.3)	2.0 (0.6,3.4)	1.9 (0.5,3.2)	2.6 (1.0,4.2)
		Max	4.1 (2.1,6.1)	3.4 (1.5,5.2)	3.9 (1.9,5.8)	4.1 (2.1,6.1)	3.8 (1.9,5.7)	5.3 (3.0,7.6)
	Greece	Min	1.3 (0.7,2.0)	1.2 (0.6,1.8)	1.1 (0.5,1.7)	1.3 (0.6,1.9)	1.5 (0.8,2.3)	1.6 (0.9,2.4)
		Max	4.1 (2.4,5.9)	2.0 (0.8,3.2)	2.7 (1.3,4.2)	3.9 (2.2,5.6)	5.8 (3.7,7.8)	6.2 (4.1,8.4)
	Norway	Min	5.3 (4.4,6.1)	4.9 (4.0,5.7)	4.3 (3.5,5.0)	6.1 (5.2,7.0)	6.9 (5.9,7.8)	4.2 (3.4,4.9)
		Max	13.4 (11.5,15.3)	12.3 (10.4,14.1)	10.9 (9.1,12.6)	15.8 (13.7,17.9)	18.2 (16.0,20.4)	10.1 (8.6,11.7)
		Min	1.3 (0.7,2.0)	NA	NA	NA	NA	1.4 (0.8,2.1)

Country	Estimate	Study period	2014	2015	2016	2017	2018
Portugal	Max	2.3 (1.3,3.2)	1.4 (0.7,2.1)	2.1 (1.2,3.0)	2.3 (1.4,3.2)	2.6 (1.6,3.6)	3.1 (1.9,4.3)
Overall	Min	2.6 (1.9,3.4)	2.3 (1.6,3.0)	2.4 (1.7,3.1)	2.8 (2.1,3.6)	2.9 (2.1,3.6)	2.8 (2.0,3.5)
	Max	5.4 (4.3,6.6)	4.4 (3.4,5.5)	4.7 (3.6,5.8)	5.9 (4.6,7.1)	6.4 (5.2,7.7)	5.7 (4.5,6.9)

The table shows the widest variability observed. In Belgium, Greece and Norway and Portugal, this means the minimum adjusted and the maximum crude estimates (In Portugal, only one of the participating centres reported an extended population, and only for 2018, so minimum estimates could only be obtained for that year). In Denmark and Finland, there was only one participating centre which searched a national or regional database (respectively), so there were no reference and extended populations, but a single population (i.e. no maximum-minimum estimates, but a single estimate). In both countries, the single crude and adjusted estimates are shown.

Abbreviations: CI, confidence interval; ILDs, interstitial lung diseases; F-ILDs, fibrosing interstitial lung diseases; IPF, idiopathic pulmonary fibrosis; NA, not available; SSc-ILD, systemic sclerosis-associated interstitial lung disease.

sTable 4. Incidence per 10^5 person-years (95%CI) of non-IPF F-ILD subtypes in each country and overall, annually and for the whole study period

	Country	Estimate	Study period	2014	2015	2016	2017	2018
**iNSIP**	Belgium	Min	2.1 (1.7,2.5)	1.2 (0.7,2.2)	2.0 (1.3,3.2)	2.4 (1.6,3.7)	2.6 (1.8,4.0)	2.1 (1.4,3.3)
		Max	8.4 (7.0,10.0)	4.8 (2.8,8.1)	7.5 (5.0,11.2)	10.1 (7.0,14.6)	11.0 (7.8,15.7)	8.8 (5.9,13.0)
	Denmark	Min	0.4 (0.3,0.5)	0.4 (0.3,0.7)	0.5 (0.4,0.8)	0.4 (0.3,0.7)	0.3 (0.2,0.6)	0.4 (0.2,0.6)
		Max	0.6 (0.5,0.7)	0.6 (0.4,0.9)	0.7 (0.5,1.0)	0.6 (0.4,0.8)	0.5 (0.3,0.7)	0.5 (0.4,0.8)
	Finland	Min	1.7 (1.2,2.4)	2.4 (1.3,4.6)	1.0 (0.4,2.7)	1.0 (0.4,2.7)	2.4 (1.2,4.5)	1.7 (0.8,3.7)
		Max	3.5 (2.7,4.4)	4.9 (3.1,7.7)	2.1 (1.0,4.1)	2.0 (1.0,4.1)	4.8 (3.1,7.6)	3.5 (2.1,6.0)
	Greece	Min	0.4 (0.3,0.5)	0.2 (0.1,0.5)	0.5 (0.3,0.8)	0.4 (0.3,0.7)	0.5 (0.3,0.8)	0.2 (0.1,0.4)
		Max	0.8 (0.7,1.0)	0.7 (0.4,1.2)	1.0 (0.7,1.5)	0.9 (0.6,1.4)	1.0 (0.7,1.5)	0.5 (0.3,0.9)
	Norway	Min	0.9 (0.8,1.1)	NA	0.8 (0.5,1.2)	1.4 (1.0,1.9)	0.9 (0.6,1.4)	0.7 (0.4,1.0)
		Max	2.4 (2.0,2.8)	NA	2.0 (1.4,2.9)	3.6 (2.8,4.7)	2.5 (1.8,3.4)	1.6 (1.1,2.3)
	Portugal	Min	0.1 (0.1,0.2)	0.1 (0.1,0.4)	0.1 (0.0,0.3)	0.2 (0.1,0.5)	0.1 (0.0,0.4)	0.2 (0.1,0.4)
		Max	0.2 (0.1,0.3)	0.2 (0.1,0.5)	0.1 (0.0,0.3)	0.2 (0.1,0.5)	0.2 (0.1,0.4)	0.2 (0.1,0.5)
	Overall	Min	1.0 (0.6,1.4)	1.0 (0.6,1.4)	0.8 (0.5,1.2)	0.9 (0.6,1.3)	1.2 (0.8,1.6)	0.9 (0.5,1.3)
		Max	2.6 (1.9,3.4)	2.2 (1.6,2.9)	2.2 (1.5,2.9)	2.9 (2.1,3.7)	3.3 (2.5,4.2)	2.5 (1.8,3.2)
**uIIPs**	Belgium	Min	0.1 (0.1,0.2)	0.1 (0.1,0.4)	0.1 (0.0,0.3)	0.2 (0.1,0.5)	0.1 (0.0,0.4)	0.2 (0.1,0.4)
		Max	13.6 (11.8,15.6)	10.6 (7.5,15.1)	13.7 (10.2,18.4)	15.8 (11.8,21.3)	13.5 (9.9,18.6)	14.4 (10.6,19.6)
	Denmark	Min	3.0 (2.8,3.2)	3.3 (2.8,3.9)	3.1 (2.7,3.7)	3.0 (2.5,3.5)	2.7 (2.2,3.2)	3.0 (2.5,3.5)
		Max	4.1 (3.9,4.4)	4.5 (3.9,5.2)	4.3 (3.8,5.0)	4.1 (3.5,4.7)	3.7 (3.1,4.3)	4.1 (3.5,4.7)
	Finland	Min	1.0 (0.6,1.5)	0.8 (0.2,2.4)	0.6 (0.2,2.2)	0.9 (0.3,2.5)	0.7 (0.2,2.3)	1.7 (0.8,3.7)
		Max	1.9 (1.4,2.7)	1.6 (0.7,3.5)	1.3 (0.5,3.1)	1.8 (0.9,3.8)	1.5 (0.7,3.4)	3.5 (2.1,6.0)
	Greece	Min	1.0 (0.6,1.5)	0.8 (0.2,2.4)	0.6 (0.2,2.2)	0.9 (0.3,2.5)	0.7 (0.2,2.3)	1.7 (0.8,3.7)
		Max	1.7 (1.4,1.9)	1.1 (0.7,1.7)	1.1 (0.8,1.7)	2.0 (1.4,2.7)	2.3 (1.7,3.0)	1.7 (1.2,2.3)

	Country	Estimate	Study period	2014	2015	2016	2017	2018
	Norway	Min	0.9 (0.7,1.1)	NA	0.7 (0.4,1.1)	1.6 (1.2,2.1)	0.5 (0.3,0.8)	0.8 (0.6,1.2)
		Max	2.3 (1.9,2.7)	NA	1.8 (1.2,2.6)	4.0 (3.1,5.2)	1.2 (0.8,2.0)	2.0 (1.4,2.8)
	Portugal	Min	0.8 (0.7,1.0)	0.5 (0.3,0.9)	0.7 (0.4,1.1)	0.7 (0.5,1.2)	1.1 (0.7,1.6)	1.1 (0.8,1.5)
		Max	1.2 (1.0,1.4)	0.7 (0.5,1.2)	0.9 (0.6,1.4)	1.0 (0.7,1.5)	1.5 (1.1,2.1)	1.5 (1.1,2.0)
	Overall	Min	1.6 (1.1,2.1)	1.5 (1.0,1.9)	1.5 (1.0,2.0)	1.7 (1.2,2.2)	1.5 (1.0,1.9)	1.8 (1.2,2.3)
		Max	4.2 (3.2,5.1)	3.7 (2.8,4.6)	3.9 (3.0,4.8)	4.8 (3.8,5.8)	4.0 (3.1,4.9)	4.5 (3.6,5.5)
**RA-ILD**	Belgium	Min	0.6 (0.5,0.6)	0.6 (0.5,0.8)	0.5 (0.4,0.6)	0.6 (0.5,0.8)	0.6 (0.5,0.8)	0.5 (0.4,0.6)
		Max	1.7 (1.5,1.9)	1.8 (1.4,2.3)	1.5 (1.2,1.9)	1.9 (1.5,2.4)	1.9 (1.5,2.4)	1.5 (1.2,1.9)
	Denmark	Min	0.5 (0.4,0.6)	0.6 (0.4,0.9)	0.5 (0.3,0.7)	0.5 (0.3,0.7)	0.5 (0.3,0.7)	0.4 (0.3,0.7)
		Max	0.7 (0.6,0.8)	0.9 (0.6,1.2)	0.6 (0.4,0.9)	0.6 (0.4,0.9)	0.6 (0.4,0.9)	0.6 (0.4,0.9)
	Finland	Min	0.9 (0.6,1.5)	1.0 (0.4,2.7)	1.3 (0.5,3.1)	0.5 (0.1,2.0)	0.7 (0.2,2.3)	1.1 (0.4,2.8)
		Max	1.9 (1.4,2.6)	2.1 (1.0,4.1)	2.6 (1.4,4.8)	1.0 (0.4,2.7)	1.5 (0.7,3.4)	2.3 (1.2,4.4)
	Greece	Min	1.0 (0.8,1.1)	0.5 (0.3,0.8)	1.0 (0.8,1.4)	1.0 (0.7,1.3)	1.1 (0.8,1.5)	1.2 (0.9,1.6)
		Max	3.1 (2.8,3.5)	3.7 (2.9,4.8)	2.8 (2.1,3.6)	2.3 (1.7,3.0)	4.0 (3.2,4.9)	3.1 (2.4,4.0)
	Norway	Min	0.5 (0.4,0.7)	NA	0.2 (0.1,0.4)	0.7 (0.4,1.1)	0.6 (0.4,0.9)	0.7 (0.5,1.1)
		Max	1.4 (1.1,1.7)	NA	0.4 (0.2,0.9)	1.7 (1.2,2.6)	1.5 (1.0,2.3)	1.7 (1.2,2.5)
	Portugal	Min	0.4 (0.3,0.5)	0.3 (0.2,0.7)	0.3 (0.1,0.6)	0.4 (0.2,0.8)	0.4 (0.2,0.8)	0.5 (0.3,0.9)
		Max	0.6 (0.4,0.7)	0.5 (0.3,0.8)	0.4 (0.2,0.7)	0.6 (0.3,1.0)	0.6 (0.4,1.0)	0.7 (0.5,1.1)
	Overall	Min	0.7 (0.4,0.9)	0.6 (0.4,0.9)	0.6 (0.4,0.9)	0.6 (0.3,0.8)	0.6 (0.4,0.9)	0.7 (0.5,1.0)
		Max	1.6 (1.1,2.1)	1.8 (1.3,2.3)	1.4 (0.9,1.8)	1.4 (0.9,1.8)	1.7 (1.2,2.2)	1.7 (1.2,2.1)
**Mixed CTD-ILD**	Belgium	Min	0.1 (0.1,0.2)	0.1 (0.1,0.2)	0.1 (0.1,0.2)	0.1 (0.1,0.2)	0.1 (0.1,0.2)	0.1 (0.1,0.2)
		Max	0.4 (0.3,0.5)	0.3 (0.2,0.6)	0.4 (0.3,0.7)	0.4 (0.2,0.6)	0.4 (0.2,0.6)	0.4 (0.3,0.7)
		Min	0.4 (0.3,0.5)	0.4 (0.2,0.6)	0.4 (0.3,0.7)	0.5 (0.3,0.8)	0.4 (0.2,0.6)	0.4 (0.3,0.7)

	Country	Estimate	Study period	2014	2015	2016	2017	2018
	Denmark	Max	0.6 (0.5,0.7)	0.5 (0.3,0.8)	0.6 (0.4,0.9)	0.7 (0.5,1.0)	0.5 (0.3,0.8)	0.6 (0.4,0.9)
	Finland	Min	1.3 (0.9,1.9)	2.8 (1.5,5.1)	0.5 (0.1,2.0)	1.3 (0.5,3.0)	1.1 (0.4,2.9)	0.9 (0.3,2.5)
		Max	2.7 (2.0,3.5)	5.7 (3.7,8.6)	1.0 (0.4,2.7)	2.6 (1.4,4.8)	2.3 (1.2,4.4)	1.8 (0.8,3.7)
	Greece	Min	0.2 (0.1,0.2)	0.1 (0.0,0.3)	0.2 (0.1,0.4)	0.1 (0.0,0.3)	0.3 (0.2,0.6)	0.2 (0.1,0.4)
		Max	0.6 (0.4,0.7)	0.5 (0.3,1.0)	0.5 (0.3,0.9)	0.2 (0.1,0.5)	1.1 (0.8,1.7)	0.5 (0.3,0.9)
	Norway	Min	0.4 (0.3,0.6)	NA	0.3 (0.1,0.6)	0.8 (0.5,1.2)	0.3 (0.1,0.5)	0.4 (0.2,0.7)
		Max	1.1 (0.9,1.4)	NA	0.7 (0.4,1.3)	2.1 (1.5,3.0)	0.7 (0.4,1.3)	0.9 (0.6,1.5)
	Portugal	Min	0.1 (0.0,0.1)	0.1 (0.0,0.3)	0.1 (0.0,0.3)	0.0 (0.0, 0.0)	0.1 (0.0,0.3)	0.1 (0.0,0.3)
		Max	0.1 (0.1,0.2)	0.1 (0.0,0.3)	0.1 (0.0,0.4)	0.0 (0.0, 0.0)	0.1 (0.0,0.4)	0.1 (0.0,0.3)
	Overall	Min	0.5 (0.3,0.7)	0.9 (0.6,1.2)	0.3 (0.1,0.4)	0.5 (0.3,0.7)	0.4 (0.2,0.6)	0.4 (0.2,0.6)
		Max	0.9 (0.5,1.3)	1.4 (1.0,1.9)	0.6 (0.3,0.9)	1.0 (0.6,1.4)	0.9 (0.5,1.2)	0.7 (0.4,1.0)
**Other CTD-ILD**	Belgium	Min	0.3 (0.2,0.3)	0.4 (0.3,0.5)	0.3 (0.2,0.4)	0.2 (0.2,0.4)	0.3 (0.2,0.4)	0.2 (0.2,0.4)
		Max	0.9 (0.8,1.0)	1.2 (0.9,1.6)	0.8 (0.6,1.2)	0.8 (0.5,1.1)	0.9 (0.6,1.2)	0.8 (0.5,1.1)
	Denmark	Min	0.6 (0.5,0.8)	0.7 (0.5,0.9)	0.6 (0.5,0.9)	0.6 (0.4,0.9)	0.6 (0.4,0.9)	0.6 (0.4,0.9)
		Max	0.9 (0.8,1.0)	0.9 (0.7,1.2)	0.9 (0.7,1.2)	0.9 (0.6,1.2)	0.9 (0.6,1.2)	0.9 (0.6,1.2)
	Finland	Min	2.5 (1.9,3.3)	2.8 (1.5,5.1)	2.1 (1.1,4.2)	3.0 (1.7,5.3)	2.5 (1.3,4.7)	2.0 (1.0,4.0)
		Max	5.1 (4.2,6.2)	5.7 (3.7,8.6)	4.4 (2.7,7.0)	6.1 (4.1,9.2)	5.1 (3.3,7.9)	4.1 (2.5,6.6)
	Greece	Min	0.4 (0.4,0.6)	0.5 (0.3,0.8)	0.3 (0.2,0.5)	0.4 (0.3,0.7)	0.5 (0.3,0.8)	0.5 (0.3,0.8)
		Max	1.3 (1.1,1.6)	2.9 (2.2,3.9)	0.7 (0.4,1.2)	1.0 (0.6,1.5)	1.2 (0.8,1.8)	1.2 (0.8,1.8)
	Norway	Min	1.1 (0.9,1.3)	NA	1.5 (1.1,2.1)	0.9 (0.6,1.4)	0.9 (0.6,1.3)	1.0 (0.7,1.4)
		Max	2.7 (2.3,3.2)	NA	3.9 (3.0,5.1)	2.4 (1.7,3.4)	2.3 (1.7,3.3)	2.3 (1.7,3.2)
	Portugal	Min	0.6 (0.5,0.8)	0.2 (0.1,0.5)	0.8 (0.5,1.3)	0.6 (0.3,1.0)	0.6 (0.4,1.0)	0.8 (0.5,1.2)
		Max	0.8 (0.7,1.0)	0.3 (0.1,0.6)	1.1 (0.8,1.7)	0.8 (0.5,1.2)	0.9 (0.6,1.3)	1.1 (0.8,1.5)

	Country	Estimate	Study period	2014	2015	2016	2017	2018
	Overall	Min	1.0 (0.7,1.4)	1.1 (0.8,1.4)	1.0 (0.7,1.4)	1.1 (0.8,1.4)	1.0 (0.7,1.3)	0.9 (0.6,1.2)
		Max	2.0 (1.4,2.5)	2.2 (1.6,2.8)	2.0 (1.4,2.5)	2.0 (1.4,2.5)	1.9 (1.3,2.4)	1.7 (1.2,2.2)
**Exposure-related ILDs**	Belgium	Min	1.3 (1.0,1.7)	2.0 (1.2,3.1)	1.4 (0.8,2.4)	0.8 (0.4,1.6)	1.5 (0.9,2.6)	0.9 (0.5,1.9)
		Max	5.3 (4.2,6.6)	7.9 (5.2,11.8)	5.0 (3.1,8.1)	3.2 (1.7,6.2)	6.4 (4.0,10.2)	3.9 (2.1,7.0)
	Denmark	Min	2.2 (2.0,2.4)	2.2 (1.8,2.7)	1.7 (1.3,2.1)	2.4 (2.0,2.9)	2.4 (2.0,2.9)	2.3 (1.9,2.8)
		Max	3.0 (2.8,3.2)	3.0 (2.6,3.6)	2.3 (1.9,2.8)	3.3 (2.8,3.9)	3.3 (2.8,3.8)	3.1 (2.7,3.7)
	Finland	Min	3.2 (2.5,4.1)	6.0 (4.0,9.0)	3.4 (2.0,5.8)	2.4 (1.3,4.5)	2.0 (1.0,4.0)	2.1 (1.1,4.2)
		Max	6.4 (5.4,7.7)	12.1 (9.1,16.2)	6.9 (4.8,10.1)	4.9 (3.1,7.6)	4.1 (2.5,6.6)	4.3 (2.7,6.9)
	Greece	Min	0.3 (0.3,0.4)	0.2 (0.1,0.4)	0.3 (0.2,0.6)	0.4 (0.3,0.7)	0.3 (0.2,0.6)	0.4 (0.3,0.7)
		Max	0.8 (0.6,1.0)	0.6 (0.3,1.2)	0.7 (0.4,1.2)	0.9 (0.6,1.4)	0.7 (0.4,1.1)	1.0 (0.6,1.5)
	Norway	Min	0.2 (0.1,0.3)	NA	0.3 (0.2,0.6)	0.2 (0.1,0.4)	0.2 (0.1,0.5)	0.1 (0.0,0.3)
		Max	0.5 (0.4,0.7)	NA	0.9 (0.5,1.5)	0.4 (0.2,0.9)	0.6 (0.3,1.2)	0.2 (0.1,0.7)
	Portugal	Min	0.2 (0.2,0.3)	0.2 (0.1,0.5)	0.0 (0.0,0.3)	0.1 (0.1,0.4)	0.5 (0.3,0.9)	0.3 (0.2,0.6)
		Max	0.3 (0.2,0.5)	0.2 (0.1,0.5)	0.0 (0.0,0.3)	0.2 (0.1,0.5)	0.7 (0.4,1.1)	0.5 (0.3,0.8)
	Overall	Min	1.5 (1.0,1.9)	2.5 (1.8,3.1)	1.3 (0.9,1.8)	1.1 (0.7,1.6)	1.2 (0.8,1.6)	1.1 (0.7,1.5)
		Max	2.9 (2.1,3.6)	4.8 (3.8,5.8)	2.6 (1.9,3.4)	2.2 (1.5,2.8)	2.6 (1.9,3.4)	2.2 (1.5,2.8)
**Sarcoidosis**	Belgium	Min	4.3 (3.7,4.9)	4.4 (3.2,6.1)	4.7 (3.5,6.4)	3.9 (2.8,5.5)	4.8 (3.5,6.4)	3.6 (2.5,5.1)
		Max	17.2 (15.2,19.5)	17.8 (13.6,23.3)	17.1 (13.2,22.3)	16.5 (12.4,22.1)	20.0 (15.4,25.9)	14.8 (10.9,20.0)
	Denmark	Min	13.7 (13.3,14.2)	14.1 (13.1,15.3)	14.4 (13.3,15.5)	13.5 (12.4,14.6)	13.2 (12.2,14.3)	13.5 (12.5,14.6)
		Max	18.9 (18.4,19.5)	19.5 (18.3,20.9)	19.8 (18.5,21.1)	18.6 (17.3,19.9)	18.2 (17.0,19.5)	18.7 (17.5,20.0)
	Finland	Min	6.2 (5.2,7.5)	9.8 (7.1,13.4)	3.4 (2.0,5.8)	4.4 (2.7,7.0)	7.1 (4.9,10.3)	6.6 (4.5,9.7)
		Max	12.7 (11.3,14.4)	19.9 (15.9,24.9)	6.9 (4.8,10.1)	9.0 (6.4,12.5)	14.5 (11.2,18.8)	13.4 (10.3,17.6)
		Min	1.4 (1.2,1.6)	0.9 (0.7,1.3)	1.3 (1.0,1.7)	1.5 (1.2,2.0)	1.8 (1.4,2.3)	1.2 (0.9,1.7)

	Country	Estimate	Study period	2014	2015	2016	2017	2018
	Greece	Max	3.1 (2.8,3.5)	2.6 (1.9,3.6)	2.8 (2.2,3.6)	3.3 (2.6,4.2)	3.9 (3.2,4.9)	2.7 (2.1,3.5)
	Norway	Min	2.0 (1.8,2.3)	NA	2.3 (1.7,2.9)	2.1 (1.6,2.7)	2.4 (1.9,3.0)	1.3 (0.9,1.8)
		Max	5.1 (4.6,5.7)	NA	5.8 (4.6,7.2)	5.5 (4.4,6.9)	6.3 (5.1,7.7)	3.2 (2.4,4.2)
	Portugal	Min	1.5 (1.3,1.8)	0.9 (0.6,1.4)	2.0 (1.5,2.6)	1.1 (0.8,1.6)	1.4 (1.0,1.9)	2.2 (1.7,2.7)
		Max	2.1 (1.9,2.4)	1.2 (0.9,1.8)	2.7 (2.2,3.5)	1.6 (1.1,2.1)	1.9 (1.4,2.5)	3.0 (2.4,3.7)
	Overall	Min	5.1 (4.2,6.0)	6.4 (5.4,7.4)	4.5 (3.7,5.4)	4.4 (3.5,5.2)	5.2 (4.3,6.1)	4.8 (3.9,5.7)
		Max	10.1 (8.7,11.5)	12.2 (10.6,13.8)	9.2 (7.8,10.6)	9.1 (7.7,10.4)	10.8 (9.3,12.3)	9.3 (8.0,10.6)
**Other F-ILDs**	Belgium	Min	2.8 (2.3,3.3)	1.4 (0.8,2.4)	1.4 (0.8,2.4)	2.2 (1.4,3.4)	3.9 (2.8,5.5)	4.9 (3.7,6.6)
		Max	11.1 (9.5,13.0)	5.5 (3.4,8.9)	5.0 (3.1,8.1)	9.4 (6.4,13.7)	16.4 (12.3,21.9)	20.4 (15.8,26.4)
	Denmark	Min	10.0 (9.6,10.4)	9.1 (8.3,10.1)	11.7 (10.7,12.7)	11.2 (10.3,12.2)	8.6 (7.7,9.4)	9.2 (8.4,10.2)
		Max	23.8 (23.1,24.4)	22.4 (21.0,23.8)	26.8 (25.3,28.3)	26.3 (24.8,27.8)	21.0 (19.8,22.4)	22.4 (21.1,23.8)
	Finland	Min	0.4 (0.2,0.8)	0.4 (0.1,1.9)	0.6 (0.2,2.2)	0.3 (0.0,1.8)	0.2 (0.0,1.8)	0.4 (0.1,1.9)
		Max	0.8 (0.5,1.3)	0.8 (0.3,2.4)	1.3 (0.5,3.1)	0.5 (0.1,2.0)	0.5 (0.1,2.0)	0.8 (0.2,2.4)
	Greece	Min	0.5 (0.4,0.6)	0.4 (0.2,0.6)	0.5 (0.3,0.8)	0.5 (0.3,0.8)	0.6 (0.4,0.9)	0.7 (0.5,1.0)
		Max	1.2 (1.0,1.4)	1.0 (0.6,1.6)	1.0 (0.7,1.5)	1.0 (0.7,1.6)	1.3 (0.9,1.9)	1.5 (1.0,2.1)
	Norway	Min	1.8 (1.5,2.0)	NA	1.9 (1.5,2.5)	2.8 (2.2,3.4)	1.2 (0.8,1.6)	1.3 (0.9,1.8)
		Max	4.5 (4.0,5.1)	NA	4.9 (3.9,6.2)	7.2 (5.9,8.7)	3.1 (2.3,4.1)	3.1 (2.4,4.1)
	Portugal	Min	0.5 (0.4,0.6)	0.6 (0.3,1.0)	0.6 (0.3,1.0)	0.4 (0.2,0.8)	0.4 (0.2,0.8)	0.4 (0.2,0.7)
		Max	0.7 (0.5,0.8)	0.8 (0.5,1.2)	0.8 (0.5,1.2)	0.6 (0.4,1.0)	0.6 (0.4,1.0)	0.6 (0.3,0.9)
	Overall	Min	2.5 (1.8,3.1)	2.2 (1.6,2.8)	2.6 (2.0,3.2)	2.7 (2.0,3.3)	2.3 (1.7,2.9)	2.6 (1.9,3.3)
		Max	7.1 (5.9,8.3)	6.1 (5.0,7.2)	6.6 (5.4,7.8)	7.5 (6.3,8.7)	7.2 (5.9,8.4)	8.1 (6.9,9.4)
**HP**	Belgium	Min	0.2 (0.1,0.4)	0.0 (0.0, 0.0)	0.3 (0.1,0.9)	0.3 (0.1,0.9)	0.3 (0.1,0.9)	0.3 (0.1,0.9)
		Max	0.8 (0.5,1.5)	0.0 (0.0, 0.0)	0.9 (0.3,2.9)	1.1 (0.3,3.3)	1.1 (0.3,3.3)	1.1 (0.3,3.3)

Country	Estimate	Study period	2014	2015	2016	2017	2018
Denmark	Min	1.2 (1.1,1.4)	1.1 (0.9,1.5)	1.2 (0.9,1.6)	1.2 (0.9,1.6)	1.3 (1.0,1.7)	1.3 (1.0,1.7)
	Max	1.7 (1.5,1.9)	1.6 (1.2,2.0)	1.7 (1.3,2.1)	1.7 (1.3,2.1)	1.8 (1.4,2.2)	1.8 (1.4,2.2)
Finland	Min	0.9 (0.6,1.5)	1.1 (0.4,2.9)	0.9 (0.3,2.5)	0.9 (0.3,2.5)	0.9 (0.3,2.5)	0.9 (0.3,2.5)
	Max	1.9 (1.4,2.6)	2.3 (1.2,4.5)	1.8 (0.9,3.8)	1.8 (0.9,3.8)	1.8 (0.8,3.7)	1.8 (0.8,3.7)
Greece	Min	1.2 (1.0,1.3)	0.4 (0.3,0.7)	1.1 (0.8,1.5)	1.4 (1.0,1.8)	1.4 (1.1,1.9)	1.5 (1.2,2.0)
	Max	2.6 (2.3,3.0)	1.2 (0.8,1.9)	2.3 (1.8,3.1)	2.9 (2.3,3.7)	3.1 (2.4,4.0)	3.2 (2.6,4.1)
Norway	Min	0.3 (0.2,0.4)	NA	0.3 (0.2,0.6)	0.3 (0.1,0.5)	0.3 (0.2,0.6)	0.3 (0.2,0.6)
	Max	0.8 (0.6,1.1)	NA	0.9 (0.5,1.5)	0.7 (0.4,1.3)	0.9 (0.5,1.5)	0.7 (0.4,1.3)
Portugal	Min	1.6 (1.4,1.8)	1.4 (1.0,2.0)	1.7 (1.3,2.3)	1.5 (1.1,2.1)	1.7 (1.2,2.3)	1.5 (1.1,2.0)
	Max	2.2 (1.9,2.4)	2.0 (1.5,2.6)	2.4 (1.8,3.1)	2.1 (1.6,2.8)	2.3 (1.8,3.0)	2.1 (1.6,2.6)
Overall	Min	0.9 (0.5,1.3)	0.9 (0.5,1.2)	0.9 (0.5,1.3)	0.9 (0.5,1.3)	1.0 (0.6,1.3)	0.9 (0.5,1.3)
	Max	1.7 (1.1,2.3)	1.4 (0.9,2.0)	1.7 (1.1,2.2)	1.7 (1.1,2.3)	1.8 (1.2,2.4)	1.8 (1.2,2.4)

The table shows the widest variability observed. In Belgium, Greece and Norway, this means the minimum adjusted and the maximum crude estimates (the participating centre in Norway could not retrieve incident cases for 2014, and thus incidence could not be estimated for that year). In Denmark and Finland, there was only one participating centre which searched a national or regional database (respectively), so there were no reference and extended populations, but a single population (i.e. no maximum-minimum estimates, but a single estimate). In these countries, the single crude and adjusted estimates are shown. In Portugal, only one of the participating centres reported an extended population, and only for 2018. Plus, incident cases for 2018 could not be retrieved in this centre, so minimum adjusted estimates could not be obtained for Portugal. Instead, the table shows the nearest estimates (maximum adjusted) as minimum values.

Abbreviations: CI, confidence interval; CTD-ILD, connective tissue disease-associated interstitial lung disease; F-ILDs, fibrosing interstitial lung diseases; HP, hypersensitivity pneumonitis; iNSIP, idiopathic non-specific interstitial pneumonia; IPF, idiopathic pulmonary fibrosis; RA-ILD, rheumatoid arthritis-associated interstitial lung disease; SSc-ILD, systemic sclerosis-associated interstitial lung disease; uIIPs, unclassifiable idiopathic interstitial pneumonias.

sTable 5. Prevalence per 10^5 persons (95%CI) of non-IPF F-ILD subtypes in each country and overall, annually and for the whole study period

Subtype	Country	Estimate	Study period	2014	2015	2016	2017	2018
**iNSIP**	Belgium	Min	3.1 (2.0,4.3)	1.5 (0.7,2.3)	2.8 (1.7,3.9)	3.7 (2.4,4.9)	3.9 (2.6,5.2)	3.7 (2.5,5.0)
		Max	12.6 (8.6,16.7)	6.2 (3.3,9.0)	10.3 (6.8,13.8)	15.5 (10.8,20.1)	16.4 (11.7,21.1)	15.5 (10.9,20.1)
	Denmark	Min	1.7 (1.3,2.1)	1.6 (1.2,1.9)	1.9 (1.5,2.3)	1.9 (1.5,2.3)	1.5 (1.1,1.8)	1.6 (1.3,2.0)
		Max	2.3 (1.9,2.8)	2.2 (1.7,2.6)	2.6 (2.2,3.1)	2.6 (2.1,3.1)	2.0 (1.6,2.4)	2.2 (1.8,2.7)
	Finland	Min	5.4 (3.1,7.7)	4.4 (2.3,6.5)	4.9 (2.7,7.1)	4.4 (2.3,6.5)	6.1 (3.7,8.5)	7.1 (4.5,9.7)
		Max	11.0 (7.7,14.3)	9.0 (6.0,12.0)	10.0 (6.9,13.2)	9.0 (6.0,11.9)	12.5 (9.0,16.0)	14.4 (10.7,18.2)
	Greece	Min	1.5 (0.8,2.1)	1.4 (0.7,2.1)	1.4 (0.7,2.1)	1.4 (0.7,2.0)	1.3 (0.6,1.9)	1.8 (1.1,2.6)
		Max	4.1 (2.4,5.9)	4.0 (2.3,5.6)	4.0 (2.3,5.7)	3.9 (2.2,5.6)	3.6 (2.0,5.3)	5.2 (3.2,7.2)
	Norway	Min	5.5 (4.6,6.4)	4.1 (3.3,4.9)	4.8 (4.0,5.6)	5.8 (4.9,6.7)	6.6 (5.7,7.6)	5.9 (5.0,6.8)
		Max	14.0 (12.1,15.9)	10.4 (8.7,12.1)	12.3 (10.5,14.1)	15.1 (13.1,17.1)	17.5 (15.3,19.6)	14.4 (12.6,16.3)
	Portugal	Min	0.5 (0.1,1.0)	NA	NA	NA	NA	0.5 (0.1,0.8)
		Max	0.9 (0.3,1.5)	0.9 (0.3,1.5)	0.9 (0.3,1.4)	0.9 (0.3,1.4)	0.9 (0.4,1.5)	1.0 (0.3,1.7)
	Overall	Min	3.1 (2.3,3.9)	2.4 (1.7,3.1)	2.9 (2.1,3.6)	3.0 (2.2,3.8)	3.5 (2.6,4.3)	3.6 (2.8,4.5)
		Max	7.5 (6.1,9.0)	5.4 (4.2,6.7)	6.7 (5.3,8.1)	7.8 (6.3,9.3)	8.8 (7.3,10.4)	8.8 (7.2,10.4)
**uIIPs**	Belgium	Min	6.0 (4.4,7.6)	5.3 (3.8,6.8)	6.4 (4.7,8.1)	6.1 (4.5,7.8)	6.6 (4.9,8.2)	5.8 (4.2,7.4)
		Max	24.3 (18.7,30.0)	21.2 (15.9,26.5)	23.4 (18.1,28.7)	25.9 (19.9,31.9)	27.4 (21.3,33.6)	23.9 (18.2,29.6)
	Denmark	Min	9.4 (8.5,10.3)	10.8 (9.8,11.7)	11.2 (10.2,12.2)	8.7 (7.8,9.5)	7.7 (6.9,8.5)	8.8 (7.9,9.6)
		Max	13.0 (11.9,14.0)	14.8 (13.7,16.0)	15.5 (14.3,16.6)	12.0 (11.0,13.0)	10.6 (9.7,11.6)	12.1 (11.1,13.1)
	Finland	Min	2.3 (0.8,3.8)	1.3 (0.1,2.4)	1.6 (0.4,2.9)	2.1 (0.7,3.6)	2.6 (1.0,4.2)	3.8 (1.9,5.8)
		Max	4.7 (2.6,6.9)	2.6 (1.0,4.2)	3.3 (1.5,5.2)	4.4 (2.3,6.4)	5.3 (3.1,7.6)	7.9 (5.1,10.6)
	Greece	Min	1.4 (0.7,2.0)	0.5 (0.1,0.9)	0.8 (0.3,1.3)	1.3 (0.7,2.0)	1.9 (1.1,2.7)	2.3 (1.5,3.2)
		Max	3.9 (2.2,5.6)	1.5 (0.5,2.5)	2.2 (1.0,3.5)	3.8 (2.1,5.5)	5.5 (3.5,7.5)	6.6 (4.4,8.8)

Subtype	Country	Estimate	Study period	2014	2015	2016	2017	2018
	Norway	Min	3.8 (3.1,4.5)	2.5 (1.9,3.1)	3.0 (2.3,3.6)	4.3 (3.5,5.1)	4.4 (3.6,5.1)	4.6 (3.8,5.3)
		Max	9.6 (8.0,11.2)	6.2 (4.9,7.6)	7.6 (6.2,9.0)	11.1 (9.4,12.9)	11.5 (9.8,13.3)	11.1 (9.5,12.7)
	Portugal	Min	2.1 (1.3,2.9)	NA	NA	NA	NA	2.6 (1.7,3.6)
		Max	3.5 (2.4,4.7)	1.8 (1.0,2.6)	2.8 (1.8,3.8)	3.6 (2.5,4.7)	4.3 (3.0,5.5)	5.7 (4.0,7.4)
	Overall	Min	4.0 (3.1,4.9)	3.4 (2.5,4.2)	3.9 (3.0,4.9)	4.0 (3.1,4.9)	4.2 (3.2,5.1)	4.5 (3.6,5.5)
		Max	9.9 (8.2,11.5)	8.0 (6.5,9.5)	9.1 (7.5,10.7)	10.1 (8.4,11.8)	10.8 (9.1,12.5)	11.2 (9.4,13.0)
**RA-ILD**	Belgium	Min	4.6 (4.2,5.0)	4.2 (3.8,4.6)	4.5 (4.1,4.9)	4.8 (4.4,5.3)	4.6 (4.2,5.0)	4.9 (4.5,5.3)
		Max	14.3 (13.1,15.4)	12.9 (11.8,14.0)	14.0 (12.8,15.1)	15.0 (13.8,16.2)	14.2 (13.1,15.4)	15.2 (14.0,16.4)
	Denmark	Min	1.3 (1.0,1.7)	1.3 (1.0,1.6)	1.2 (0.9,1.5)	1.4 (1.0,1.7)	1.5 (1.1,1.8)	1.3 (1.0,1.7)
		Max	1.8 (1.4,2.2)	1.8 (1.4,2.2)	1.6 (1.3,2.0)	1.9 (1.5,2.3)	2.0 (1.6,2.4)	1.9 (1.5,2.2)
	Finland	Min	3.1 (1.3,4.8)	2.0 (0.6,3.4)	2.9 (1.2,4.6)	3.3 (1.5,5.1)	3.4 (1.6,5.2)	3.8 (1.9,5.8)
		Max	6.3 (3.8,8.8)	4.1 (2.1,6.2)	5.9 (3.5,8.3)	6.7 (4.1,9.2)	6.9 (4.3,9.5)	7.9 (5.1,10.6)
	Greece	Min	2.5 (1.6,3.4)	1.9 (1.1,2.7)	2.1 (1.3,2.9)	2.7 (1.7,3.6)	2.9 (1.9,3.9)	3.0 (2.0,4.0)
		Max	16.0 (12.6,19.4)	13.0 (9.9,16.0)	13.2 (10.1,16.4)	16.7 (13.2,20.2)	19.0 (15.2,22.7)	18.1 (14.4,21.7)
	Norway	Min	0.8 (0.5,1.1)	0.1 (0.0,0.2)	0.4 (0.1,0.6)	1.1 (0.7,1.5)	1.4 (0.9,1.8)	1.0 (0.7,1.4)
		Max	2.1 (1.3,2.8)	0.2 (0.0,0.5)	0.9 (0.4,1.4)	2.9 (2.0,3.7)	3.6 (2.6,4.6)	2.5 (1.7,3.3)
	Portugal	Min	1.9 (1.1,2.6)	NA	NA	NA	NA	1.9 (1.1,2.7)
		Max	3.1 (2.0,4.2)	2.3 (1.4,3.3)	2.7 (1.7,3.7)	3.2 (2.1,4.2)	3.5 (2.4,4.6)	4.1 (2.7,5.6)
	Overall	Min	2.4 (1.9,2.9)	1.8 (1.4,2.3)	2.2 (1.7,2.7)	2.6 (2.0,3.1)	2.7 (2.1,3.3)	2.7 (2.1,3.3)
		Max	7.3 (6.1,8.4)	5.7 (4.7,6.8)	6.4 (5.3,7.5)	7.7 (6.5,8.9)	8.2 (6.9,9.4)	8.3 (7.0,9.5)
**Mixed CTD-ILD**	Belgium	Min	0.2 (0.1,0.3)	0.2 (0.1,0.3)	0.2 (0.1,0.3)	0.2 (0.1,0.3)	0.2 (0.1,0.3)	0.2 (0.1,0.3)
		Max	0.6 (0.4,0.9)	0.6 (0.4,0.9)	0.6 (0.4,0.9)	0.6 (0.3,0.8)	0.6 (0.4,0.9)	0.6 (0.4,0.9)
		Min	2.4 (2.0,2.9)	2.6 (2.1,3.0)	2.6 (2.1,3.1)	2.3 (1.9,2.8)	2.3 (1.8,2.7)	2.3 (1.9,2.8)

Subtype	Country	Estimate	Study period	2014	2015	2016	2017	2018
	Denmark	Max	3.3 (2.8,3.9)	3.5 (3.0,4.1)	3.6 (3.0,4.1)	3.2 (2.7,3.7)	3.1 (2.6,3.6)	3.2 (2.7,3.7)
	Finland	Min	2.7 (1.1,4.3)	2.9 (1.2,4.6)	1.6 (0.4,2.9)	2.5 (0.9,4.1)	3.1 (1.4,4.9)	3.4 (1.5,5.2)
		Max	5.5 (3.2,7.9)	5.9 (3.5,8.4)	3.3 (1.5,5.2)	5.1 (2.9,7.4)	6.4 (3.9,8.9)	6.8 (4.3,9.4)
	Greece	Min	0.4 (0.1,0.8)	0.3 (0.0,0.7)	0.3 (0.0,0.5)	0.4 (0.0,0.7)	0.6 (0.1,1.0)	0.6 (0.1,1.0)
		Max	2.5 (1.2,3.9)	2.1 (0.8,3.3)	1.4 (0.4,2.4)	2.2 (0.9,3.5)	3.6 (1.9,5.2)	3.4 (1.8,5.0)
	Norway	Min	2.8 (2.2,3.4)	1.2 (0.7,1.6)	1.6 (1.1,2.1)	3.6 (2.9,4.3)	3.7 (3.0,4.4)	3.8 (3.1,4.5)
		Max	7.2 (5.8,8.6)	2.9 (2.0,3.8)	4.0 (3.0,5.1)	9.3 (7.7,10.9)	9.8 (8.2,11.4)	9.3 (7.9,10.8)
	Portugal	Min	0.2 (0.0,0.5)	NA	NA	NA	NA	0.2 (0.0,0.5)
		Max	0.3 (0.0,0.7)	0.2 (0.1,0.5)	0.3 (0.0,0.6)	0.3 (0.0,0.7)	0.5 (0.1,0.9)	0.5 (0.0,1.0)
	Overall	Min	1.5 (1.1,2.0)	1.3 (0.9,1.7)	1.1 (0.7,1.5)	1.6 (1.2,2.0)	1.8 (1.3,2.2)	1.8 (1.4,2.3)
		Max	3.2 (2.4,4.0)	2.5 (1.8,3.3)	2.2 (1.6,2.9)	3.5 (2.6,4.3)	4.0 (3.1,4.9)	4.0 (3.1,4.9)
**Other CTD-ILD**	Belgium	Min	1.1 (0.9,1.3)	1.1 (0.9,1.4)	1.1 (0.9,1.3)	1.1 (0.8,1.3)	1.2 (0.9,1.4)	1.1 (0.9,1.4)
		Max	3.5 (2.9,4.1)	3.5 (3.0,4.1)	3.5 (2.9,4.0)	3.3 (2.7,3.8)	3.6 (3.0,4.1)	3.6 (3.0,4.1)
	Denmark	Min	2.4 (2.0,2.9)	2.5 (2.0,2.9)	2.4 (2.0,2.9)	2.4 (2.0,2.9)	2.4 (1.9,2.8)	2.4 (1.9,2.8)
		Max	3.3 (2.8,3.9)	3.4 (2.8,3.9)	3.4 (2.8,3.9)	3.3 (2.8,3.8)	3.3 (2.8,3.8)	3.3 (2.7,3.8)
	Finland	Min	6.1 (3.7,8.6)	6.1 (3.6,8.5)	5.7 (3.3,8.0)	7.0 (4.4,9.7)	5.9 (3.5,8.2)	6.1 (3.6,8.5)
		Max	12.5 (9.0,16.0)	12.4 (8.9,15.9)	11.6 (8.2,15.0)	14.3 (10.6,18.1)	12.0 (8.5,15.4)	12.4 (8.9,15.9)
	Greece	Min	1.3 (0.6,1.9)	1.4 (0.7,2.1)	1.0 (0.5,1.6)	1.3 (0.7,2.0)	1.3 (0.7,1.9)	1.2 (0.6,1.8)
		Max	6.7 (4.5,8.9)	8.2 (5.8,10.6)	5.3 (3.4,7.3)	7.2 (4.9,9.5)	6.9 (4.7,9.2)	5.9 (3.8,7.9)
	Norway	Min	6.8 (5.8,7.7)	4.8 (4.0,5.7)	7.7 (6.6,8.7)	7.0 (6.0,7.9)	7.7 (6.7,8.7)	6.5 (5.6,7.4)
		Max	17.2 (15.1,19.4)	12.1 (10.3,13.9)	19.7 (17.4,22.0)	18.1 (15.9,20.3)	20.3 (18.0,22.6)	15.9 (14.0,17.9)
	Portugal	Min	1.7 (0.9,2.4)	NA	NA	NA	NA	2.4 (1.5,3.3)
		Max	2.8 (1.8,3.8)	1.1 (0.5,1.8)	2.1 (1.3,3.0)	2.6 (1.7,3.6)	3.4 (2.3,4.6)	5.2 (3.6,6.8)

Subtype	Country	Estimate	Study period	2014	2015	2016	2017	2018
	Overall	Min	3.4 (2.8,4.1)	3.0 (2.4,3.6)	3.4 (2.7,4.0)	3.7 (3.0,4.3)	3.6 (2.9,4.3)	3.4 (2.8,4.1)
		Max	7.7 (6.5,8.9)	6.8 (5.6,7.9)	7.6 (6.4,8.8)	8.1 (6.9,9.4)	8.2 (7.0,9.5)	7.7 (6.5,8.9)
**Exposure-related ILDs**	Belgium	Min	4.9 (3.5,6.4)	5.8 (4.2,7.4)	5.2 (3.7,6.7)	5.2 (3.7,6.7)	4.2 (2.8,5.5)	4.3 (2.9,5.6)
		Max	19.9 (14.7,25.0)	23.3 (17.7,28.8)	19.0 (14.2,23.8)	21.9 (16.4,27.4)	17.5 (12.6,22.3)	17.6 (12.7,22.5)
	Denmark	Min	3.9 (3.4,4.5)	4.1 (3.5,4.7)	3.3 (2.8,3.8)	4.1 (3.5,4.6)	3.8 (3.2,4.3)	4.5 (3.9,5.1)
		Max	5.4 (4.8,6.1)	5.7 (5.0,6.4)	4.6 (3.9,5.2)	5.6 (4.9,6.3)	5.2 (4.5,5.8)	6.2 (5.5,6.9)
	Finland	Min	17.7 (13.5,21.8)	20.1 (15.7,24.6)	19.8 (15.4,24.2)	17.3 (13.2,21.4)	16.3 (12.3,20.3)	14.9 (11.1,18.7)
		Max	36.1 (30.1,42.0)	41.1 (34.7,47.5)	40.4 (34.1,46.7)	35.3 (29.4,41.2)	33.3 (27.6,39.0)	30.4 (25.0,35.8)
	Greece	Min	0.6 (0.2,1.1)	0.3 (0.0,0.6)	0.5 (0.1,0.8)	0.7 (0.2,1.2)	0.8 (0.3,1.3)	1.0 (0.4,1.5)
		Max	1.8 (0.7,3.0)	0.8 (0.0,1.6)	1.3 (0.3,2.3)	2.1 (0.8,3.3)	2.3 (1.0,3.6)	2.7 (1.3,4.1)
	Norway	Min	0.5 (0.2,0.7)	0.6 (0.3,0.9)	0.6 (0.3,0.9)	0.3 (0.1,0.5)	0.5 (0.2,0.7)	0.4 (0.1,0.6)
		Max	1.2 (0.6,1.7)	1.5 (0.8,2.1)	1.6 (0.9,2.2)	0.8 (0.4,1.3)	1.2 (0.7,1.8)	0.9 (0.4,1.3)
	Portugal	Min	0.6 (0.1,1.0)	NA	NA	NA	NA	1.6 (0.9,2.4)
		Max	1.0 (0.4,1.6)	0.5 (0.1,1.0)	0.4 (0.0,0.8)	0.5 (0.1,0.9)	0.5 (0.1,1.0)	3.6 (2.2,4.9)
	Overall	Min	5.6 (4.6,6.7)	6.3 (5.1,7.5)	6.0 (4.9,7.2)	5.6 (4.5,6.7)	5.2 (4.1,6.2)	5.2 (4.1,6.2)
		Max	10.9 (9.2,12.7)	12.1 (10.3,14.0)	11.2 (9.4,13.0)	11.0 (9.3,12.8)	10.0 (8.3,11.7)	10.2 (8.5,11.9)
**Sarcoidosis**	Belgium	Min	16.6 (13.9,19.3)	13.9 (11.4,16.3)	16.4 (13.8,19.1)	17.0 (14.3,19.8)	17.7 (14.9,20.5)	18.0 (15.2,20.8)
		Max	67.0 (57.6,76.4)	55.8 (47.2,64.3)	60.2 (51.7,68.6)	71.9 (62.0,81.9)	74.1 (64.0,84.2)	74.2 (64.2,84.3)
	Denmark	Min	60.4 (58.1,62.6)	63.8 (61.4,66.1)	65.1 (62.8,67.5)	57.5 (55.3,59.7)	55.3 (53.1,57.4)	60.3 (58.1,62.6)
		Max	83.3 (80.6,85.9)	88.0 (85.2,90.8)	89.8 (87.1,92.6)	79.3 (76.7,81.9)	76.3 (73.7,78.8)	83.2 (80.6,85.9)
	Finland	Min	24.9 (19.9,29.8)	27.2 (22.0,32.4)	22.4 (17.7,27.1)	22.3 (17.6,27.0)	24.8 (19.9,29.7)	27.6 (22.4,32.7)
		Max	50.8 (43.7,57.8)	55.6 (48.1,63.0)	45.8 (39.1,52.5)	45.6 (38.9,52.3)	50.6 (43.6,57.6)	56.2 (48.8,63.6)
		Min	5.0 (3.7,6.3)	4.8 (3.5,6.0)	4.2 (3.0,5.4)	4.9 (3.7,6.2)	5.5 (4.1,6.8)	5.7 (4.4,7.1)

Subtype	Country	Estimate	Study period	2014	2015	2016	2017	2018
	Greece	Max	14.3 (11.1,17.6)	13.5 (10.4,16.7)	12.0 (9.1,15.0)	14.1 (10.9,17.3)	15.6 (12.3,19.0)	16.4 (12.9,19.9)
	Norway	Min	7.7 (6.7,8.7)	10.3 (9.1,11.5)	7.4 (6.4,8.5)	6.4 (5.5,7.4)	8.0 (7.0,9.0)	6.5 (5.6,7.4)
		Max	19.6 (17.3,21.9)	25.9 (23.2,28.6)	19.0 (16.8,21.3)	16.7 (14.6,18.8)	21.2 (18.8,23.5)	15.9 (14.0,17.8)
	Portugal	Min	10.8 (8.9,12.6)	NA	NA	NA	NA	10.7 (8.9,12.6)
		Max	18.0 (15.4,20.7)	14.5 (12.2,16.8)	16.8 (14.4,19.3)	17.9 (15.3,20.4)	19.1 (16.5,21.8)	23.3 (19.9,26.7)
	Overall	Min	21.2 (19.1,23.4)	22.0 (19.8,24.2)	21.3 (19.1,23.4)	20.2 (18.1,22.3)	21.0 (18.9,23.1)	21.8 (19.7,23.9)
		Max	42.3 (38.8,45.7)	42.2 (38.7,45.7)	40.6 (37.2,44.0)	40.9 (37.5,44.3)	42.8 (39.4,46.3)	44.9 (41.3,48.4)
**Other F-ILDs**	Belgium	Min	4.1 (2.7,5.4)	2.4 (1.4,3.4)	2.2 (1.2,3.2)	3.4 (2.2,4.6)	5.2 (3.7,6.7)	7.1 (5.3,8.8)
		Max	16.3 (11.7,21.0)	9.6 (6.0,13.1)	8.1 (5.0,11.2)	14.4 (9.9,18.8)	21.7 (16.3,27.2)	29.2 (22.9,35.5)
	Denmark	Min	22.4 (21.0,23.8)	16.8 (15.6,18.0)	23.9 (22.5,25.4)	22.5 (21.2,23.9)	20.8 (19.5,22.1)	27.8 (26.3,29.4)
		Max	55.0 (52.8,57.2)	45.4 (43.4,47.4)	58.4 (56.2,60.7)	54.8 (52.6,56.9)	51.2 (49.1,53.2)	64.9 (62.6,67.3)
	Finland	Min	1.1 (0.1,2.1)	0.8 (0.1,1.6)	1.3 (0.1,2.4)	1.1 (0.1,2.2)	1.0 (0.0,2.0)	1.4 (0.2,2.5)
		Max	2.3 (0.8,3.7)	1.6 (0.3,2.8)	2.6 (1.0,4.2)	2.3 (0.8,3.8)	2.0 (0.6,3.4)	2.8 (1.1,4.4)
	Greece	Min	0.8 (0.3,1.3)	0.6 (0.1,1.0)	0.6 (0.2,1.0)	0.7 (0.2,1.2)	0.9 (0.4,1.5)	1.1 (0.5,1.7)
		Max	2.2 (0.9,3.4)	1.6 (0.5,2.6)	1.7 (0.6,2.8)	2.0 (0.8,3.2)	2.6 (1.2,4.0)	3.1 (1.6,4.5)
	Norway	Min	7.8 (6.8,8.8)	4.8 (4.0,5.7)	6.7 (5.7,7.7)	8.9 (7.8,10.1)	9.1 (8.0,10.2)	9.1 (8.0,10.2)
		Max	19.9 (17.6,22.2)	12.1 (10.3,13.9)	17.1 (15.0,19.3)	23.2 (20.8,25.7)	24.0 (21.5,26.5)	22.2 (20.0,24.5)
	Portugal	Min	1.6 (0.9,2.4)	NA	NA	NA	NA	1.7 (1.0,2.5)
		Max	2.7 (1.7,3.8)	1.8 (1.0,2.6)	2.4 (1.5,3.4)	2.8 (1.8,3.9)	3.2 (2.1,4.2)	3.8 (2.4,5.1)
	Overall	Min	5.9 (4.7,7.0)	4.1 (3.2,5.1)	5.7 (4.6,6.8)	6.0 (4.9,7.1)	6.1 (4.9,7.2)	7.5 (6.2,8.7)
		Max	16.4 (14.3,18.6)	12.0 (10.2,13.9)	15.1 (13.0,17.1)	16.6 (14.4,18.7)	17.4 (15.2,19.7)	21.0 (18.6,23.4)
**HP**	Belgium	Min	1.1 (0.4,1.8)	0.3 (0.1,0.6)	0.8 (0.2,1.3)	1.0 (0.4,1.7)	1.3 (0.5,2.0)	2.1 (1.2,3.1)
		Max	4.4 (2.0,6.8)	1.0 (0.1,2.2)	2.8 (1.0,4.6)	4.3 (1.9,6.8)	5.3 (2.6,8.0)	8.8 (5.3,12.2)

Subtype	Country	Estimate	Study period	2014	2015	2016	2017	2018
	Denmark	Min	3.4 (2.9,3.9)	3.4 (2.9,4.0)	3.4 (2.9,3.9)	3.2 (2.7,3.7)	3.6 (3.1,4.2)	3.3 (2.8,3.9)
		Max	4.7 (4.1,5.3)	4.7 (4.1,5.4)	4.7 (4.1,5.3)	4.4 (3.8,5.0)	5.0 (4.3,5.6)	4.6 (4.0,5.2)
	Finland	Min	3.3 (1.5,5.0)	3.0 (1.3,4.8)	2.6 (1.0,4.3)	3.4 (1.6,5.2)	3.6 (1.7,5.5)	3.6 (1.7,5.5)
		Max	6.7 (4.1,9.2)	6.2 (3.7,8.7)	5.4 (3.1,7.7)	6.9 (4.3,9.5)	7.4 (4.7,10.1)	7.3 (4.7,10.0)
	Greece	Min	2.5 (1.6,3.4)	1.4 (0.7,2.0)	1.6 (0.9,2.3)	2.3 (1.5,3.2)	3.3 (2.2,4.3)	4.0 (2.9,5.2)
		Max	7.2 (4.9,9.5)	3.9 (2.2,5.6)	4.6 (2.7,6.4)	6.7 (4.5,9.0)	9.4 (6.8,12.0)	11.5 (8.6,14.4)
	Norway	Min	1.9 (1.4,2.4)	2.6 (2.0,3.2)	1.8 (1.3,2.3)	1.4 (1.0,1.9)	1.7 (1.3,2.2)	1.9 (1.4,2.4)
		Max	4.8 (3.7,5.9)	6.5 (5.2,7.9)	4.6 (3.5,5.7)	3.8 (2.8,4.8)	4.6 (3.5,5.7)	4.6 (3.5,5.6)
	Portugal	Min	6.1 (4.8,7.5)	NA	NA	NA	NA	6.6 (5.2,8.1)
		Max	10.3 (8.3,12.3)	7.0 (5.4,8.6)	9.2 (7.3,11.0)	10.5 (8.5,12.4)	11.6 (9.5,13.6)	14.3 (11.7,17.0)
	Overall	Min	3.1 (2.3,4.0)	2.6 (1.9,3.4)	2.8 (2.0,3.5)	3.1 (2.3,4.0)	3.6 (2.7,4.5)	3.5 (2.7,4.4)
		Max	6.4 (5.1,7.7)	4.9 (3.7,6.1)	5.2 (4.0,6.4)	6.1 (4.8,7.4)	7.2 (5.8,8.6)	8.5 (7.0,10.1)

The table shows the widest variability observed. In Belgium, Greece and Norway and Portugal, this means the minimum adjusted and the maximum crude estimates (In Portugal, only one of the participating centres reported an extended population, and only for 2018, so minimum estimates could only be obtained for that year). In Denmark and Finland, there was only one participating centre which searched a national or regional database (respectively), so there were no reference and extended populations, but a single population (i.e. no maximum-minimum estimates, but a single estimate). In both countries, the single crude and adjusted estimates are shown.

Abbreviations: CI, confidence interval; CTD-ILD, connective tissue disease-associated interstitial lung disease; F-ILDs, fibrosing interstitial lung diseases; HP, hypersensitivity pneumonitis; iNSIP, idiopathic non-specific interstitial pneumonia; IPF, idiopathic pulmonary fibrosis; RA-ILD, rheumatoid arthritis-associated interstitial lung disease; SSc-ILD, systemic sclerosis-associated interstitial lung disease; uIIPs, unclassifiable idiopathic interstitial pneumonias.

sTable 6. Relative percentage (95%CI) of each non-IPF F-ILD subtype in each country, annually and for the whole study period

Country	Subtype	Study period	2014	2015	2016	2017	2018
**Belgium**	iNSIP	3.0 (2.0,3.9)	1.7 (0.9,2.4)	2.8 (1.8,3.7)	3.6 (2.5,4.7)	3.6 (2.6,4.6)	3.2 (2.3,4.2)
	uIIPs	5.7 (4.4,7.0)	5.8 (4.4,7.2)	6.3 (4.9,7.7)	6.0 (4.7,7.4)	6.0 (4.7,7.3)	5.0 (3.9,6.2)
	RA-ILD	47.0 (44.2,49.8)	48.2 (45.2,51.2)	47.6 (44.8,50.4)	51.0 (48.2,53.8)	45.5 (42.7,48.2)	46.0 (43.3,48.7)
	Mixed CTD-ILD	2.0 (1.2,2.8)	2.4 (1.5,3.3)	2.1 (1.3,2.9)	1.9 (1.1,2.7)	2.0 (1.2,2.7)	1.9 (1.2,2.7)
	Other CTD-ILD	11.5 (9.7,13.3)	13.2 (11.2,15.2)	11.8 (10.0,13.6)	11.2 (9.4,13.0)	11.4 (9.7,13.1)	10.8 (9.1,12.4)
	Exposure-related ILDs	4.7 (3.5,5.9)	6.3 (4.9,7.8)	5.1 (3.9,6.4)	5.1 (3.9,6.4)	3.8 (2.8,4.9)	3.7 (2.7,4.7)
	Sarcoidosis	15.8 (13.8,17.9)	15.1 (13.0,17.3)	16.3 (14.2,18.4)	16.8 (14.7,18.9)	16.2 (14.2,18.3)	15.6 (13.6,17.5)
	Other F-ILDs	3.9 (2.8,4.9)	2.6 (1.6,3.6)	2.2 (1.4,3.0)	3.4 (2.3,4.4)	4.8 (3.6,5.9)	6.1 (4.8,7.4)
	SSc-ILD	5.4 (4.1,6.6)	4.5 (3.2,5.7)	5.1 (3.8,6.3)	6.0 (4.7,7.4)	5.6 (4.3,6.8)	5.9 (4.6,7.1)
	HP	1.0 (0.5,1.6)	0.3 (0.0,0.6)	0.8 (0.3,1.3)	1.0 (0.4,1.6)	1.2 (0.6,1.8)	1.8 (1.1,2.6)
**Denmark**	iNSIP	1.6 (1.3,1.9)	1.4 (1.2,1.7)	1.6 (1.3,1.9)	1.8 (1.4,2.1)	1.5 (1.2,1.8)	1.4 (1.1,1.7)
	uIIPs	8.8 (8.1,9.5)	9.9 (9.1,10.6)	9.5 (8.9,10.2)	8.2 (7.5,8.8)	7.8 (7.1,8.5)	7.6 (7.0,8.3)
	RA-ILD	1.2 (1.0,1.5)	1.2 (0.9,1.4)	1.0 (0.8,1.2)	1.3 (1.0,1.6)	1.5 (1.2,1.8)	1.2 (0.9,1.4)
	Mixed CTD-ILD	2.3 (1.9,2.6)	2.3 (2.0,2.7)	2.2 (1.9,2.5)	2.2 (1.8,2.5)	2.3 (1.9,2.7)	2.0 (1.7,2.4)
	Other CTD-ILD	2.3 (1.9,2.6)	2.2 (1.9,2.6)	2.1 (1.7,2.4)	2.3 (1.9,2.6)	2.4 (2.0,2.8)	2.1 (1.7,2.4)
	Exposure-related ILDs	3.7 (3.2,4.1)	3.8 (3.3,4.2)	2.8 (2.4,3.2)	3.8 (3.4,4.3)	3.8 (3.3,4.3)	3.9 (3.5,4.4)
	Sarcoidosis	56.3 (55.1,57.5)	58.4 (57.2,59.5)	55.4 (54.2,56.5)	54.1 (52.9,55.3)	55.9 (54.7,57.1)	52.6 (51.5,53.8)

Country	Subtype	Study period	2014	2015	2016	2017	2018
	Other F-ILDs	20.9 (19.9,21.9)	15.4 (14.5,16.3)	20.3 (19.4,21.3)	21.2 (20.2,22.2)	21.0 (20.0,22.0)	24.3 (23.3,25.3)
	SSc-ILD	2.1 (1.8,2.4)	2.3 (2.0,2.7)	2.2 (1.8,2.5)	2.1 (1.8,2.5)	1.6 (1.3,2.0)	2.0 (1.7,2.3)
	HP	3.2 (2.8,3.6)	3.1 (2.7,3.5)	2.9 (2.5,3.3)	3.0 (2.6,3.4)	3.7 (3.2,4.1)	2.9 (2.5,3.3)
**Finland**	iNSIP	7.9 (5.6,10.1)	6.4 (4.3,8.4)	7.8 (5.4,10.1)	6.7 (4.6,8.9)	8.9 (6.5,11.3)	10.1 (7.6,12.5)
	uIIPs	3.4 (1.9,4.9)	1.8 (0.7,2.9)	2.6 (1.2,4.0)	3.3 (1.7,4.8)	3.8 (2.2,5.4)	5.5 (3.6,7.3)
	RA-ILD	4.5 (2.8,6.2)	2.9 (1.5,4.3)	4.6 (2.8,6.4)	5.0 (3.1,6.9)	4.9 (3.1,6.7)	5.5 (3.6,7.3)
	Mixed CTD-ILD	4.0 (2.3,5.6)	4.2 (2.5,5.9)	2.6 (1.2,4.0)	3.8 (2.2,5.5)	4.5 (2.8,6.3)	4.8 (3.0,6.5)
	Other CTD-ILD	9.0 (6.6,11.4)	8.7 (6.4,11.1)	9.0 (6.5,11.5)	10.7 (8.1,13.4)	8.5 (6.2,10.9)	8.6 (6.3,11.0)
	Exposure-related ILDs	25.8 (22.1,29.5)	29.0 (25.2,32.8)	31.3 (27.3,35.4)	26.4 (22.7,30.2)	23.8 (20.2,27.3)	21.2 (17.8,24.5)
	Sarcoidosis	36.3 (32.3,40.3)	39.2 (35.1,43.3)	35.5 (31.3,39.7)	34.1 (30.0,38.2)	36.1 (32.1,40.1)	39.2 (35.1,43.2)
	Other F-ILDs	1.6 (0.6,2.7)	1.1 (0.2,2.0)	2.0 (0.8,3.2)	1.7 (0.6,2.8)	1.5 (0.5,2.5)	1.9 (0.8,3.1)
	SSc-ILD	2.9 (1.5,4.3)	2.4 (1.1,3.6)	3.0 (1.5,4.5)	3.1 (1.6,4.5)	2.7 (1.4,4.1)	3.7 (2.1,5.3)
	HP	4.8 (3.0,6.5)	4.4 (2.7,6.1)	4.2 (2.4,6.0)	5.2 (3.3,7.1)	5.3 (3.4,7.1)	5.1 (3.3,6.9)
**Greece**	iNSIP	8.4 (5.0,11.7)	11.3 (6.7,15.8)	10.4 (6.2,14.6)	8.0 (4.7,11.4)	6.3 (3.6,9.1)	8.1 (5.2,11.0)
	uIIPs	7.9 (4.7,11.2)	4.3 (1.4,7.2)	5.8 (2.6,9.0)	7.8 (4.5,11.1)	9.6 (6.3,12.9)	10.4 (7.1,13.6)
	RA-ILD	14.6 (10.3,18.9)	15.6 (10.4,20.8)	15.7 (10.7,20.7)	15.8 (11.3,20.2)	14.6 (10.6,18.6)	13.6 (9.9,17.2)
	Mixed CTD-ILD	2.4 (0.6,4.3)	2.7 (0.4,5.0)	1.9 (0.0,3.7)	2.2 (0.4,3.9)	2.8 (1.0,4.7)	2.6 (0.9,4.3)
	Other CTD-ILD	7.3 (4.1,10.4)	11.3 (6.7,15.8)	7.6 (4.0,11.3)	7.9 (4.6,11.2)	6.5 (3.7,9.3)	5.4 (3.0,7.9)

Country	Subtype	Study period	2014	2015	2016	2017	2018
	Exposure-related ILDs	3.7 (1.4,6.0)	2.3 (0.2,4.5)	3.3 (0.9,5.8)	4.2 (1.7,6.7)	4.0 (1.8,6.2)	4.2 (2.1,6.4)
	Sarcoidosis	29.0 (23.5,34.5)	38.7 (31.7,45.7)	31.1 (24.7,37.4)	28.9 (23.3,34.4)	27.4 (22.3,32.4)	25.6 (20.9,30.3)
	Other F-ILDs	4.4 (1.9,6.9)	4.5 (1.5,7.5)	4.4 (1.6,7.3)	4.0 (1.6,6.4)	4.6 (2.2,7.0)	4.8 (2.5,7.0)
	SSc-ILD	7.7 (4.5,11.0)	9.5 (5.3,13.7)	8.0 (4.3,11.8)	7.4 (4.2,10.7)	7.8 (4.7,10.7)	7.3 (4.5,10.1)
	HP	14.6 (10.3,18.9)	11.1 (6.6,15.6)	11.8 (7.4,16.3)	13.8 (9.6,18.0)	16.4 (12.2,20.6)	18.0 (13.9,22.1)
**Norway**	iNSIP	12.9 (11.2,14.5)	13.3 (11.3,15.3)	12.6 (10.8,14.3)	12.9 (11.3,14.5)	13.3 (11.8,14.8)	13.5 (11.9,15.1)
	uIIPs	8.8 (7.4,10.2)	8.0 (6.4,9.6)	7.8 (6.4,9.2)	9.5 (8.1,10.9)	8.8 (7.5,10.0)	10.4 (9.0,11.8)
	RA-ILD	1.9 (1.2,2.6)	0.3 (0.0,0.6)	0.9 (0.4,1.5)	2.4 (1.7,3.2)	2.8 (2.0,3.5)	2.3 (1.6,3.1)
	Mixed CTD-ILD	6.6 (5.4,7.8)	3.7 (2.6,4.9)	4.1 (3.1,5.2)	8.0 (6.7,9.3)	7.4 (6.2,8.6)	8.7 (7.4,10.1)
	Other CTD-ILD	15.8 (14.0,17.6)	15.5 (13.4,17.7)	20.1 (18.0,22.3)	15.5 (13.8,17.2)	15.4 (13.8,17.0)	14.9 (13.2,16.6)
	Exposure-related ILDs	1.1 (0.6,1.6)	1.9 (1.1,2.7)	1.6 (0.9,2.3)	0.7 (0.3,1.1	0.93 (0.5,1.4)	0.8 (0.4,1.2)
	Sarcoidosis	18.0 (16.1,19.9)	33.2 (30.4,36.0)	19.5 (17.4,21.6)	14.3 (12.6,16.0)	16.1 (14.4,17.7)	14.8 (13.2,16.5)
	Other F-ILDs	18.3 (16.4,20.2)	15.5 (13.4,17.7)	17.5 (15.5,19.5)	20.0 (18.0,21.8)	18.2 (16.5,19.9)	20.8 (18.9,22.7)
	SSc-ILD	12.3 (10.7,13.9)	15.7 (13.5,17.9)	11.1 (9.5,12.8)	13.5 (11.9,15.2)	13.8 (12.2,15.3)	9.5 (8.1,10.9)
	HP	4.4 (3.4,5.4)	8.4 (6.7,10.0)	4.7 (3.6,5.8)	3.2 (2.4,4.1)	3.5 (2.7,4.3)	4.3 (3.3,5.2)
**Portugal**	iNSIP	2.0 (0.7,3.3)	2.9 (1.1,4.7)	2.2 (0.8,3.5)	1.9 (0.7,3.2)	1.9 (0.7,3.1)	1.6 (0.5,2.6)
	uIIPs	7.8 (5.3,10.3)	5.6 (3.1,8.0)	7.1 (4.6,9.5)	8.1 (5.6,10.5)	8.6(6.2,11.04)	8.9 (6.4,11.4)
	RA-ILD	6.9 (4.6,9.3)	7.4 (4.6,10.2)	6.8 (4.4,9.2)	7.1 (4.8,9.4)	7.0 (4.8,9.2)	6.4 (4.3,8.6)

Country	Subtype	Study period	2014	2015	2016	2017	2018
	Mixed CTD-ILD	0.8 (0.0,1.6)	0.6 (0.0,1.4)	0.7 (0.0,1.5)	0.7 (0.0,1.5)	1.0 (0.1,1.8)	0.8 (0.0,1.5)
	Other CTD-ILD	6.2 (4.0,8.4)	3.6 (1.6,5.6)	5.4 (3.2,7.5)	5.9 (3.8,8.1)	6.9 (4.8,9.1)	8.1 (5.7,10.5)
	Exposure-related ILDs	2.1 (0.8,3.5)	1.7 (0.3,3.0)	1.1 (0.1,2.1)	1.1 (0.1,2.0)	1.0 (0.2,1.9)	5.5 (3.5,7.5)
	Sarcoidosis	40.1 (35.6,44.7)	45.8 (40.4,51.1)	42.4 (37.7,47.2)	40.1 (35.7,44.6)	38.6 (34.4,42.7)	36.1 (31.8,40.3)
	Other F-ILDs	6.1 (3.9,8.3)	5.7 (3.2,8.2)	6.1 (3.8,8.4)	6.4 (4.2,8.6)	6.4 (4.3,8.5)	5.8 (3.8,7.9)
	SSc-ILD	5.0 (3.0,7.1)	4.5 (2.3,6.8)	5.3 (3.1,7.4)	5.2 (3.2,7.2)	5.2 (3.3,7.1)	4.8 (2.9,6.7)
	HP	22.9 (19.0,26.8)	22.3 (17.8,26.8)	23.1 (19.0,27.1)	23.5 (19.6,27.3)	23.4 (19.7,27.0)	22.2 (18.5,25.8)

Percentages calculated based on the total number of prevalent cases of non-IPF F-ILD in each country.
Abbreviations: CI, confidence interval; CTD-ILD, connective tissue disease-associated interstitial lung disease; F-ILDs, fibrosing interstitial lung diseases; HP, hypersensitivity pneumonitis; iNSIP, idiopathic non-specific interstitial pneumonia; IPF, idiopathic pulmonary fibrosis; RA-ILD, rheumatoid arthritis-associated interstitial lung disease; SSc-ILD, systemic sclerosis-associated interstitial lung disease; uIIPs, unclassifiable idiopathic interstitial pneumonias.

sTable 7. Incidence per 10^5 person-years and prevalence per 10^5 persons (95%CI) of non-F-ILDs in each country and overall, annually and for the whole study period

	Country	Estimate	Study period	2014	2015	2016	2017	2018
**Incidence**	Belgium	Min	1.2 (0.9,1.6)	0.8 (0.4,1.6)	1.1 (0.6,2.1)	1.2 (0.7,2.2)	1.1 (0.6,2.1)	1.8 (1.1,2.9)
		Max	4.8 (3.8,6.1)	3.1 (1.6,5.9)	4.1 (2.4,7.0)	5.0 (3.0,8.5)	4.6 (2.7,8.0)	7.4 (4.8,11.3)
	Denmark	Min	1.0 (0.9,1.2)	1.0 (0.7,1.3)	1.0 (0.8,1.3)	1.1 (0.8,1.4)	1.0 (0.8,1.4)	1.1 (0.8,1.4)
		Max	1.4 (1.3,1.6)	1.4 (1.1,1.7)	1.4 (1.1,1.8)	1.5 (1.2,1.9)	1.4 (1.1,1.8)	1.5 (1.1,1.9)
	Finland	Min	3.8 (3.0,4.8)	3.7 (2.2,6.2)	2.5 (1.3,4.7)	4.4 (2.7,7.0)	3.6 (2.1,6.1)	4.8 (3.1,7.6)
		Max	7.8 (6.6,9.1)	7.5 (5.2,10.8)	5.1 (3.3,8.0)	9.0 (6.4,12.5)	7.4 (5.1,10.6)	9.9 (7.2,13.5)
	Greece	Min	0.8 (0.7,0.9)	0.5 (0.3,0.8)	0.6 (0.4,0.9)	0.8 (0.6,1.2)	1.0 (0.7,1.3)	1.0 (0.7,1.4)
		Max	1.8 (1.5,2.0)	1.4 (1.0,2.2)	1.3 (0.9,1.9)	1.7 (1.2,2.4)	2.1 (1.5,2.8)	2.1 (1.6,2.9)
	Norway	Min	1.3 (1.1,1.5)	NA	1.3 (0.9,1.8)	2.1 (1.6,2.7)	1.0 (0.7,1.4)	0.8 (0.5,1.2)
		Max	3.2 (2.8,3.7)	NA	3.2 (2.4,4.3)	5.4 (4.3,6.8)	2.6 (1.9,3.6)	2.0 (1.4,2.8)
	Portugal	Min	0.9 (0.7,1.1)	0.9 (0.6,1.4)	0.7 (0.5,1.2)	1.2 (0.8,1.7)	0.8 (0.5,1.2)	0.7 (0.5,1.1)
		Max	1.2 (1.0,1.4)	1.3 (0.9,1.8)	1.0 (0.7,1.5)	1.7 (1.2,2.3)	1.1 (0.8,1.6)	1.0 (0.7,1.4)
	Overall	Min	1.7 (1.1,2.2)	1.6 (1.1,2.1)	1.3 (0.8,1.7)	2.0 (1.4,2.5)	1.6 (1.1,2.1)	1.9 (1.3,2.5)
		Max	3.4 (2.5,4.2)	2.9 (2.1,3.7)	2.7 (1.9,3.4)	4.1 (3.1,5.0)	3.2 (2.4,4.0)	4.0 (3.1,4.8)
**Prevalence**	Belgium	Min	1.6 (0.7,2.4)	0.9 (0.2,1.5)	1.2 (0.5,1.9)	1.7 (0.8,2.6)	1.5 (0.7,2.3)	2.6 (1.5,3.6)
		Max	6.3 (3.4,9.2)	3.4 (1.3,5.5)	4.4 (2.1,6.7)	7.2 (4.0,10.3)	6.4 (3.5,9.4)	10.6 (6.8,14.3)
	Denmark	Min	2.6 (2.1,3.0)	2.7 (2.2,3.2)	2.5 (2.0,3.0)	2.5 (2.1,3.0)	2.5 (2.1,3.0)	2.6 (2.1,3.1)
		Max	3.6 (3.0,4.1)	3.7 (3.2,4.3)	3.4 (2.9,4.0)	3.5 (2.9,4.0)	3.5 (2.9,4.0)	3.6 (3.0,4.1)
	Finland	Min	7.9 (5.2,10.7)	6.5 (3.9,9.0)	5.9 (3.5,8.3)	8.5 (5.6,11.4)	8.6 (5.7,11.5)	10.2 (7.0,13.3)
		Max	16.2 (12.2,20.2)	13.2 (9.6,16.8)	12.1 (8.6,15.5)	17.4 (13.3,21.6)	17.5 (13.4,21.7)	20.8 (16.3,25.3)
		Min	1.1 (0.5,1.7)	0.7 (0.2,1.2)	0.7 (0.2,1.2)	0.9 (0.4,1.5)	1.4 (0.7,2.0)	1.9 (1.1,2.7)

Country	Estimate	Study period	2014	2015	2016	2017	2018
Greece	Max	3.2 (1.6,4.7)	1.9 (0.8,3.1)	2.0 (0.8,3.2)	2.7 (1.3,4.1)	3.9 (2.2,5.6)	5.4 (3.4,7.4)
Norway	Min	4.9 (4.0,5.7)	4.2 (3.4,5.0)	4.7 (3.9,5.5)	6.8 (5.8,7.7)	4.8 (4.0,5.6)	3.9 (3.2,4.6)
	Max	12.4 (10.6,14.2)	10.5 (8.8,12.2)	12.1 [illegible]	17.5 (15.4,19.7)	12.6 (10.8,14.4)	9.6 (8.1,11.1)
Portugal	Min	1.6 (0.9,2.3)	NA	NA	NA	NA	2.4 (1.6,3.3)
	Max	2.7 (1.7,3.8)	1.6 (0.8,2.3)	2.1 (1.2,3.0)	2.5 (1.6,3.5)	2.9 (1.9,3.9)	5.3 (3.7,6.9)
Overall	Min	3.6 (2.7,4.5)	2.9 (2.1,3.7)	3.0 (2.2,3.8)	4.0 (3.1,4.9)	3.8 (2.9,4.7)	4.3 (3.4,5.3)
	Max	7.4 (6.0,8.9)	5.7 (4.5,7.0)	6.0 (4.7,7.3)	8.5 (6.9,10.0)	7.8 (6.3,9.3)	9.2 (7.6,10.8)

The table shows the widest variability observed. In Belgium, Greece and Norway, this means the minimum adjusted and the maximum crude estimates. The participating centre in Norway could not retrieve incident cases for 2014, and thus incidence could not be estimated for that year. In Portugal, only one of the participating centres reported an extended population, and only for 2018. Therefore, minimum prevalence estimates could only be obtained for that year. Plus, incident cases for 2018 could not be retrieved in this centre, so minimum adjusted incidence estimates could not be obtained for Portugal. Instead, the table shows the nearest estimates (maximum adjusted) as minimum values. In Denmark and Finland, there was only one participating centre which searched a national or regional database (respectively), so there were no reference and extended populations, but a single population (i.e. no maximum-minimum estimates, but a single estimate). In both countries, the single crude and adjusted estimates are shown.

Abbreviations: CI, confidence interval; ILD, interstitial lung disease; F-ILDs, fibrosing interstitial lung diseases; IPF, idiopathic pulmonary fibrosis; NA, not available; SSc-ILD, systemic sclerosis-associated interstitial lung disease.

sTable 8. Relative percentage (95% CI) of PF behaviour only, UIP-like pattern only, both or none among total non-IPF F-ILD cases and by subtype (all countries)

Subtype	PF only	UIP-like only	PF plus UIP-like	Non-PF-non-UIP-like
iNSIP	22.5 (10.8,34.1)	8.2 (0.5,15.8)	12.2 (3.1,21.4)	57.1 (43.3,71.0)
uIIPs	24.3 (14.5,34.1)	18.9 (10.0,27.8)	17.6 (8.9,26.2)	39.2 (28.1,50.3)
RA-ILD	11.1 (1.9,20.3)	33.3 (19.6,47.1)	26.7 (13.7,39.6)	28.9 (15.6,42.1)
Mixed CTD-ILD	0.0 (0.0,0.0)	10.0 (0.0,28.6)	20.0 (0.0, 44.8)	70.0 (41.6,98.4)
Other CTD-ILD	14.6 (4.6,24.6)	16.7 (6.1,27.2)	25.0 (12.8,37.3)	43.8 (29.7,57.8)
Exposure-related ILDs	18.2 (5.0,31.3)	9.1 (0.0,18.9)	18.2 (5.0,31.3)	54.6 (37.6,71.5)
Sarcoidosis	8.5 (4.3,12.8)	4.9 (1.6,8.2)	1.8 (0.0,3.9)	84.8 (79.3,90.3)
Other F-ILDs	25.0 (14.5,34.8)	16.0 (7.3,24.6)	8.7 (2.0,15.3)	50.7 (38.9,62.52)
SSc-ILD	5.3 (0.0,11.1)	38.6 (26.5,51.2)	31.6 (19.5,43.7)	24.6 (13.4,35.7)
HP	27.4 (19.6,35.3)	10.5 (5.1,15.9)	22.6 (15.2,30.0)	39.5 (30.9,48.1)
Total non-IPF F-ILDs	16.2 (13.1,19.2)	14.4 (11.5,17.3)	14.0 (11.1,16.9)	55.5 (51.3,59.6)

Only centres reporting complete data (i.e. all non-IPF F-ILD subtypes, and both pulmonary and rheumatology cases) were considered. Percentages were calculated based on the total number of cases for each subtype, and in the last row, based on the total number of non-IPF F-ILD cases.

Abbreviations: CI, confidence interval; CTD-ILD, connective tissue disease-associated interstitial lung disease; F-ILDs, fibrosing interstitial lung diseases; HP, hypersensitivity pneumonitis; iNSIP, idiopathic non-specific interstitial pneumonia; IPF, idiopathic pulmonary fibrosis; PF, progressive-fibrosing; RA-ILD, rheumatoid arthritis-associated interstitial lung disease; SSc-ILD, systemic sclerosis-associated interstitial lung disease; ; uIIPs, unclassifiable idiopathic interstitial pneumonias; UIP, usual interstitial pneumonia.

sTable 9. Exploratory p-values for pairwise comparisons of PF behaviour percentage between non-IPF F-ILD subtypes (all countries)

Subtype	iNSIP	uIIPs	RA-ILD	Mixed CTD-ILD	Other CTD-ILD	Exposure-related ILDs	Sarcoidosis	Other F-ILDs	SSc-ILD	HP
iNSIP	-	**0.0072**	0.9019	**0.0015**	0.0683	0.2583	1.0000	0.3749	**0.0366**	**<0.0001**
uIIPs	-	-	**0.0087**	**<0.0001**	0.3857	**0.0002**	**0.0072**	0.0619	0.5818	**0.0032**
RA-ILD	-	-	-	**0.0010**	0.0820	0.2054	0.9019	0.4357	**0.0442**	**<0.0001**
Mixed CTD-ILD	-	-	-	-	**<0.0001**	**0.0263**	**0.0015**	**0.0001**	**<0.0001**	**<0.0001**
Other CTD-ILD	-	-	-	-	-	**0.0037**	0.0683	0.3287	0.7666	**0.0002**
Exposure-related ILDs	-	-	-	-	-	-	0.2583	**0.0445**	**0.0016**	**<0.0001**
Sarcoidosis	-	-	-	-	-	-	-	0.3749	**0.0366**	**<0.0001**
Other F-ILDs	-	-	-	-	-	-	-	-	0.2069	**<0.0001**
SSc-ILD	-	-	-	-	-	-	-	-	-	**0.0008**
HP	-	-	-	-	-	-	-	-	-	-

P-values below the significance level of p<0.05 are shown in bold.

Abbreviations: CTD-ILD, connective tissue disease-associated interstitial lung disease; F-ILDs, fibrosing interstitial lung diseases; HP, hypersensitivity pneumonitis; iNSIP, idiopathic non-specific interstitial pneumonia; IPF, idiopathic pulmonary fibrosis; PF, progressive-fibrosing; RA-ILD, rheumatoid arthritis-associated interstitial lung disease; SSc-ILD, systemic sclerosis-associated interstitial lung disease; uIIPs, unclassifiable idiopathic interstitial pneumonias.

sTable 10. Incidence per 10^5 person-years (95%CI) of PF-ILDs in each country and overall, annually and for the whole study period. Primary analysis (Method 1) and sensitivity analyses (Methods 1b-3)

Country	Method	Estimate	Study period	2014	2015	2016	2017	2018
**Belgium**	1	Min	5.8 (5.5,6.1)	4.6 (4.1,5.2)	5.6 (5.0,6.4)	5.7 (5.1,6.4)	6.9 (6.2,7.7)	6.5 (5.8,7.3)
		Max	13.7 (13.0,14.6)	12.0 (10.5,13.8)	12.8 (11.2,14.6)	13.7 (12.0,15.6)	15.9 (14.0,18.0)	14.5 (12.7,16.5)
	1b	Min	5.8 (5.5,6.1)	4.6 (4.1,5.2)	5.6 (5.0,6.4)	5.7 (5.1,6.4)	6.9 (6.2,7.7)	6.5 (5.8,7.3)
		Max	13.7 (13.0,14.6)	12.0 (10.5,13.8)	12.8 (11.2,14.6)	13.7 (12.0,15.6)	15.9 (14.0,18.0)	14.5 (12.7,16.5)
	2	Min	**5.6 (5.2,6.0)**	**4.4 (3.7,5.3)**	**5.4 (4.6,6.4)**	**5.4 (4.6,6.4)**	**6.6 (5.7,7.7)**	**6.2 (5.3,7.3)**
		Max	22.1 (20.3,24.1)	17.5 (14.3,21.4)	19.6 (16.1,23.9)	22.6 (18.6,27.4)	27.3 (22.8,32.7)	25.3 (21.0,30.6)
	2b	Min	**5.6 (5.3,6.1)**	**4.4 (3.9,5.2)**	**5.4 (4.8,6.4)**	**5.4 (4.8,6.4)**	**6.6 (5.9,7.7)**	**6.2 (5.5,7.3)**
		Max	13.1 (12.4,14.0)	11.5 (10.0,13.3)	12.2 (10.6,14.0)	13.1 (11.4,15.0)	15.2 (13.4,17.2)	13.8 (12.1,15.8)
	3	Min	6.3 (5.7,6.9)	5.0 (4.0,6.3)	6.1 (4.9,7.6)	6.1 (5.0,7.6)	7.5 (6.2,9.1)	7.0 (5.7,8.6)
		Max	**25.1 (23.8,26.4)**	**19.8 (17.6,22.3)**	**22.2 (19.7,24.9)**	**25.5 (22.8,28.6)**	**30.9 (27.8,34.4)**	**28.7 (25.6,32.1)**
**Denmark**	1	Min	8.5 (8.3,8.5)	8.5 (7.9,8.9)	9.1 (8.5,9.4)	8.8 (8.2,9.2)	8.0 (7.4,8.3)	8.3 (7.7,8.6)
		Max	14.4 (13.9,14.9)	14.3 (13.2,15.5)	15.3 (14.2,16.5)	15.0 (14.0,16.2)	13.4 (12.4,14.5)	14.0 (12.9,15.1)
	1b	Min	**8.3 (8.0,8.5)**	**8.2 (7.6,8.9)**	**8.8 (8.2,9.4)**	**8.5 (7.9,9.2)**	**7.7 (7.1,8.3)**	**8.0 (7.5,8.6)**
		Max	13.9 (13.5,14.4)	13.8 (12.8,15.0)	14.8 (13.7,16.0)	14.5 (13.5,15.7)	13.0 (12.0,14.1)	13.5 (12.5,14.6)
	2	Min	11.5 (11.2,11.8)	11.5 (10.8,12.2)	12.2 (11.5,13.0)	11.9 (11.2,12.7)	10.7 (10.1,11.4)	11.2 (10.5,11.9)
		Max	**19.4 (18.8,20.0)**	**19.3 (18.0,20.6)**	**20.6 (19.3,22.0)**	**20.3 (19.0,21.6)**	**18.1 (16.9,19.4)**	**18.8 (17.6,20.1)**
	2b	Min	10.4 (10.1,8.5)	10.4 (9.7,8.9)	11.1 (10.4,9.4)	10.8 (10.1,9.2)	9.7 (9.1,8.3)	10.1 (9.5,8.6)
		Max	17.6 (17.0,18.1)	17.5 (16.3,18.7)	18.7 (17.4,20.0)	18.4 (17.1,19.6)	16.4 (15.3,17.6)	17.1 (15.9,18.3)
	3	Min	9.2 (8.8,9.6)	9.1 (8.3,10.0)	9.7 (8.9,10.7)	9.6 (8.7,10.5)	8.5 (7.7,9.4)	8.9 (8.1,9.8)
		Max	12.6 (12.4,12.9)	12.5 (12.1,13.1)	13.4 (12.9,13.9)	13.2 (12.7,13.7)	11.8 (11.3,12.3)	12.3 (11.8,12.7)
		Min	5.0 (4.5,4.5)	7.6 (6.3,7.5)	3.9 (3.0,4.1)	4.0 (3.1,4.2)	4.7 (3.7,4.9)	4.8 (3.8,5.0)

Country	Method	Estimate	Study period	2014	2015	2016	2017	2018
**Finland**	1	Max	10.1 (8.8,11.6)	15.4 (12.0,19.9)	7.9 (5.5,11.2)	8.1 (5.7,11.5)	9.5 (6.9,13.1)	9.7 (7.1,13.4)
	1b	Min	**4.0 (3.6,4.5)**	**6.1 (5.0,7.5)**	**3.1 (2.4,4.1)**	**3.2 (2.5,4.2)**	**3.8 (3.0,4.9)**	**3.9 (3.0,5.0)**
		Max	8.2 (7.0,9.6)	12.5 (9.4,16.6)	6.4 (4.3,9.5)	6.6 (4.5,9.7)	7.7 (5.4,11.0)	7.9 (5.6,11.2)
	2	Min	6.7 (6.2,7.3)	10.2 (8.8,11.9)	5.2 (4.2,6.5)	5.4 (4.3,6.6)	6.3 (5.2,7.6)	6.4 (5.3,7.8)
		Max	**13.7 (12.1,15.4)**	**20.8 (16.7,25.9)**	**10.6 (7.8,14.4)**	**10.9 (8.1,14.8)**	**12.9 (9.8,16.9)**	**13.2 (10.0,17.3)**
	2b	Min	**4.0 (3.6,4.5)**	**6.1 (5.0,7.5)**	**3.1 (2.4,4.1)**	**3.2 (2.5,4.2)**	**3.8 (3.0,4.9)**	**3.9 (3.0,5.0)**
		Max	8.2 (7.0,9.6)	12.5 (9.4,16.6)	6.4 (4.3,9.5)	6.6 (4.5,9.7)	7.7 (5.4,11.0)	7.9 (5.6,11.2)
	3	Min	**4.0 (3.2,5.0)**	**6.1 (4.1,9.2)**	**3.1 (1.8,5.5)**	**3.2 (1.9,5.6)**	**3.8 (2.3,6.3)**	**3.9 (2.3,6.4)**
		Max	8.2 (7.7,8.8)	12.5 (11.0,14.3)	6.4 (5.3,7.7)	6.6 (5.5,7.9)	7.7 (6.6,9.1)	7.9 (6.7,9.3)
**Greece**	1	Min	2.1 (1.9,1.9)	1.3 (1.0,1.5)	2.0 (1.6,2.0)	2.3 (1.9,2.2)	2.7 (2.3,2.6)	2.3 (2.0,2.3)
		Max	5.2 (4.8,5.7)	5.1 (4.1,6.3)	4.5 (3.7,5.5)	4.8 (4.0,5.9)	6.6 (5.6,7.8)	5.0 (4.1,6.0)
	1b	Min	1.8 (1.6,1.9)	1.2 (0.9,1.5)	1.6 (1.3,2.0)	1.8 (1.5,2.2)	2.2 (1.8,2.6)	1.9 (1.6,2.3)
		Max	4.3 (3.9,4.7)	4.8 (3.9,6.0)	3.7 (3.0,4.6)	3.9 (3.1,4.8)	5.3 (4.4,6.4)	4.0 (3.2,4.9)
	2	Min	2.2 (2.1,2.4)	1.3 (1.1,1.7)	2.1 (1.7,2.5)	2.4 (2.0,2.8)	2.8 (2.4,3.3)	2.5 (2.1,2.9)
		Max	**5.5 (5.0,6.0)**	**5.4 (4.3,6.6)**	**4.8 (3.9,5.8)**	**5.1 (4.2,6.2)**	**6.9 (5.9,8.2)**	**5.2 (4.3,6.3)**
	2b	Min	1.8 (1.7,1.9)	1.2 (1.0,1.5)	1.7 (1.4,2.0)	1.9 (1.6,2.2)	2.2 (1.9,2.6)	2.0 (1.6,2.3)
		Max	4.5 (4.1,4.9)	5.1 (4.1,6.3)	3.9 (3.1,4.8)	4.0 (3.2,4.9)	5.5 (4.5,6.6)	4.1 (3.3,5.1)
	3	Min	**1.5 (1.4,1.7)**	**0.9 (0.7,1.3)**	**1.4 (1.1,1.9)**	**1.7 (1.3,2.1)**	**2.0 (1.6,2.5)**	**1.7 (1.3,2.2)**
		Max	3.8 (3.6,4.0)	3.7 (3.3,4.2)	3.3 (2.9,3.7)	3.5 (3.1,3.9)	4.8 (4.3,5.3)	3.6 (3.2,4.0)
**Norway**	1	Min	2.7 (2.5,2.9)	NA	2.7 (2.2,3.2)	3.6 (3.1,4.2)	2.4 (2.0,2.8)	2.1 (1.8,2.6)
		Max	6.8 (6.2,7.5)	NA	6.8 (5.6,8.3)	9.4 (7.9,11.1)	6.2 (5.1,7.7)	5.2 (4.2,6.4)
	1b	Min	**2.7 (2.5,2.9)**	NA	**2.7 (2.3,3.2)**	**3.6 (3.1,4.2)**	**2.4 (2.0,2.8)**	**2.1 (1.8,2.6)**
		Max	6.9 (6.3,7.6)	NA	6.9 (5.6,8.4)	9.5 (8.0,11.2)	6.3 (5.1,7.7)	5.2 (4.2,6.4)

Country	Method	Estimate	Study period	2014	2015	2016	2017	2018
	2	Min	3.2 (2.9,3.4)	NA	3.2 (2.7,3.7)	4.3 (3.7,4.9)	2.8 (2.4,3.3)	2.5 (2.1,3.0)
		Max	**8.1 (7.4,8.9)**	NA	**8.1 (6.7,9.7)**	**11.1 (9.5,13.0)**	**7.4 (6.1,8.9)**	**6.1 (5.0,7.4)**
	2b	Min	**2.7 (2.5,2.9)**	NA	**2.7 (2.3,3.2)**	**3.6 (3.1,4.2)**	**2.4 (2.0,2.8)**	**2.1 (1.8,2.6)**
		Max	6.9 (6.3,7.6)	NA	6.9 (5.6,8.4)	9.5 (8.0,11.2)	6.3 (5.1,7.7)	5.2 (4.2,6.4)
	3	Min	**2.7 (2.4,3.0)**	NA	**2.7 (2.1,3.4)**	**3.6 (3.0,4.4)**	**2.4 (1.9,3.0)**	**2.1 (1.7,2.7)**
		Max	6.9 (6.5,7.3)	NA	6.9 (6.2,7.7)	9.5 (8.6,10.4)	6.3 (5.6,7.0)	5.2 (4.6,5.8)
**Portugal**	1	Min	2.1 (1.9,2.9)	1.5 (1.2,2.5)	2.1 (1.7,3.2)	1.8 (1.5,2.8)	2.3 (1.9,3.4)	2.6 (2.2,3.6)
		Max	2.9 (2.6,3.2)	2.1 (1.6,2.8)	2.9 (2.3,3.6)	2.5 (2.0,3.3)	3.2 (2.5,3.9)	3.5 (2.9,4.3)
	1b	Min	2.6 (2.4,2.9)	2.1 (1.7,2.5)	2.7 (2.3,3.2)	2.3 (1.9,2.8)	2.9 (2.5,3.4)	3.1 (2.7,3.6)
		Max	**3.6 (3.3,4.0)**	**2.9 (2.3,3.6)**	**3.7 (3.0,4.6)**	**3.2 (2.6,4.0)**	**4.0 (3.3,4.9)**	4.2 (3.6,5.0)
	2	Min	**1.9 (1.7,2.1)**	**1.4 (1.1,1.8)**	**1.9 (1.5,2.3)**	**1.7 (1.3,2.1)**	**2.1 (1.7,2.5)**	**2.3 (2.0,2.8)**
		Max	2.6 (2.4,2.9)	1.9 (1.4,2.6)	2.6 (2.0,3.3)	2.3 (1.8,3.0)	2.9 (2.3,3.6)	3.2 (2.6,3.9)
	2b	Min	2.6 (2.4,2.9)	2.0 (1.7,2.5)	2.6 (2.2,3.2)	2.3 (1.9,2.8)	2.8 (2.4,3.4)	2.9 (2.5,3.6)
		Max	3.5 (3.2,3.9)	2.8 (2.2,3.6)	3.6 (3.0,4.5)	3.1 (2.5,3.9)	3.9 (3.2,4.7)	4.0 (3.4,4.8)
	3	Min	2.6 (2.3,2.9)	1.9 (1.4,2.5)	2.6 (2.0,3.3)	2.3 (1.8,3.0)	2.8 (2.2,3.6)	3.2 (2.6,3.9)
		Max	**3.6 (3.3,3.8)**	2.6 (2.2,3.1)	3.5 (3.1,4.1)	3.1 (2.7,3.7)	3.9 (3.4,4.5)	**4.4 (3.9,4.9)**
**Overall**	1	Min	4.4 (3.7,5.0)	5.0 (4.2,5.5)	4.1 (3.5,4.7)	4.3 (3.6,4.8)	4.4 (3.7,5.0)	4.4 (3.7,4.9)
		Max	9.0 (7.8,10.3)	9.8 (8.5,11.2)	8.4 (7.3,9.6)	8.9 (7.8,10.2)	9.1 (7.9,10.5)	8.6 (7.5,9.9)
	1b	Min	4.2 (3.5,5.0)	4.6 (3.9,5.5)	4.0 (3.3,4.7)	4.1 (3.4,4.8)	4.2 (3.5,5.0)	4.2 (3.5,4.9)
		Max	8.5 (7.4,9.8)	9.2 (8.0,10.6)	8.0 (7.0,9.3)	8.6 (7.4,9.8)	8.7 (7.5,10.0)	8.2 (7.1,9.5)
	2	Min	5.3 (4.6,6.3)	6.2 (5.3,7.3)	4.9 (4.2,5.8)	5.1 (4.4,6.0)	5.2 (4.5,6.2)	5.2 (4.4,6.1)
		Max	**12.1 (10.6,13.8)**	**13.0 (11.4,14.8)**	**11.1 (9.7,12.6)**	**12.1 (10.6,13.7)**	**12.6 (11.0,14.4)**	**12.0 (10.5,13.7)**
		Min	4.5 (3.8,5.0)	5.0 (4.3,5.5)	4.3 (3.6,4.7)	4.4 (3.7,4.8)	4.5 (3.8,5.0)	4.4 (3.8,4.9)

Country	Method	Estimate	Study period	2014	2015	2016	2017	2018
	2b	Max	9.1 (8.0,10.4)	9.9 (8.7,11.3)	8.6 (7.5,9.8)	9.1 (8.0,10.4)	9.2 (8.0,10.5)	8.7 (7.6,9.9)
	3	Min	**4.1 (3.3,4.9)**	**4.5 (3.6,5.3)**	**3.9 (3.1,4.6)**	**4.0 (3.2,4.8)**	**4.1 (3.3,4.9)**	**4.1 (3.3,4.9)**
		Max	10.2 (8.8,11.6)	10.2 (8.8,11.7)	9.3 (7.9,10.7)	10.2 (8.8,11.7)	10.9 (9.4,12.4)	10.3 (8.9,11.7)

Within each method, the table shows the widest variability observed. This means the minimum adjusted and the maximum crude estimates. The participating centre in Norway could not retrieve incident cases for 2014, and thus incidence could not be estimated for that year. In Portugal, only one of the participating centres reported an extended population, and only for 2018. Plus, incident cases for 2018 could not be retrieved in this centre, so minimum adjusted incidence estimates could not be obtained for Portugal. Instead, the table shows the nearest estimates (maximum adjusted) as minimum values. Values highlighted in bold are the lowest and highest obtained in the sensitivity analyses (as a whole) per country and time period.

Abbreviations: CI, confidence interval; NA, not available; PF-ILD, interstitial lung disease.

sTable 11. Prevalence per 10^5 persons (95%CI) of PF-ILDs in each country and overall, annually and for the whole study period. Primary analysis (Method 1) and sensitivity analyses (Methods 1b-3)

Country	Method	Estimate	Study period	2014	2015	2016	2017	2018
**Belgium**	1	Min	16.7 (15.0,20.4)	13.5 (12.0,16.7)	15.7 (14.0,19.2)	17.2 (15.4,21.0)	18.2 (16.3,22.1)	19.5 (17.5,23.5)
		Max	65.3 (59.3,71.3)	52.4 (47.2,57.6)	56.2 (51.0,61.5)	70.1 (63.7,76.5)	73.8 (67.2,80.5)	78.0 (71.2,84.8)
	1b	Min	18.5 (16.7,20.4)	15.1 (13.5,16.7)	17.4 (15.7,19.2)	19.1 (17.2,21.0)	20.1 (18.1,22.1)	21.5 (19.4,23.5)
		Max	64.6 (58.7,70.6)	52.4 (47.3,57.6)	56.4 (51.1,61.7)	69.0 (62.7,75.3)	72.3 (65.8,78.9)	76.4 (69.7,83.2)
	2	Min	**15.6 (13.9,17.3)**	**12.6 (11.1,14.1)**	**14.6 (13.0,16.3)**	**16.0 (14.3,17.8)**	**17.0 (15.2,18.8)**	**18.2 (16.3,20.1)**
		Max	60.9 (55.1,66.7)	48.9 (43.9,53.8)	52.4 (47.3,57.5)	65.4 (59.2,71.5)	68.8 (62.4,75.3)	72.7 (66.2,79.3)
	2b	Min	17.7 (15.8,20.4)	14.4 (12.8,16.7)	16.6 (14.9,19.2)	18.2 (16.3,21.0)	19.1 (17.2,22.1)	20.4 (18.4,23.5)
		Max	60.8 (55.0,66.6)	49.3 (44.3,54.3)	53.1 (48.0,58.2)	64.9 (58.7,71.0)	68.0 (61.6,74.3)	71.8 (65.3,78.3)
	3	Min	17.7 (15.8,19.5)	14.3 (12.7,15.9)	16.6 (14.8,18.3)	18.2 (16.3,20.0)	19.2 (17.3,21.2)	20.6 (18.5,22.6)
		Max	**69.0 (62.8,75.1)**	**55.3 (50.0,60.6)**	**59.4 (53.9,64.8)**	**74.0 (67.5,80.5)**	**77.9 (71.1,84.8)**	**82.3 (75.3,89.3)**
**Denmark**	1	Min	25.3 (NE)	25.2 (NE)	27.1 (NE)	24.5 (NE)	23.2 (NE)	26.5 (NE)
		Max	40.4 (38.6,42.3)	39.9 (38.1,41.8)	43.3 (41.4,45.2)	39.3 (37.4,41.1)	37.1 (35.4,38.9)	42.6 (40.7,44.5)
	1b	Min	**25.3 (NE)**	**25.2 (NE)**	**27.1 (NE)**	**24.5 (NE)**	**23.1 (NE)**	**26.4 (NE)**
		Max	40.4 (38.6,42.3)	39.9 (38.0,41.7)	43.3 (41.4,45.2)	39.2 (37.4,41.1)	37.1 (35.3,38.9)	42.6 (40.7,44.5)
	2	Min	38.7 (NE)	38.6 (NE)	41.6 (NE)	37.6 (NE)	35.5 (NE)	40.5 (NE)
		Max	**62.0 (59.7,64.3)**	**61.1 (58.8,63.4)**	**66.4 (64.0,68.7)**	**60.2 (57.9,62.4)**	**56.9 (54.7,59.0)**	**65.3 (63.0,67.6)**
	2b	Min	35.1 (NE)	35.0 (NE)	37.7 (NE)	34.0 (NE)	32.2 (NE)	36.8 (NE)
		Max	56.2 (54.0,58.4)	55.4 (53.2,57.6)	60.1 (57.9,62.4)	54.5 (52.4,56.7)	51.6 (49.5,53.7)	59.3 (57.0,61.5)
	3	Min	29.2 (27.7,30.8)	28.8 (27.3,30.4)	31.3 (29.7,32.9)	28.4 (26.8,29.9)	26.8 (25.3,28.3)	30.8 (29.2,32.4)
		Max	40.3 (38.5,42.2)	39.8 (37.9,41.6)	43.2 (41.3,45.1)	39.2 (37.3,41.0)	37.0 (35.2,38.8)	42.5 (40.6,44.4)
		Min	18.4 (14.2,22.4)	19.5 (15.1,23.5)	17.8 (13.6,21.7)	17.4 (13.3,21.3)	17.9 (13.7,21.8)	19.5 (15.1,23.5)

Country	Method	Estimate	Study period	2014	2015	2016	2017	2018
**Finland**	1	Max	37.6 (31.5,43.7)	39.7 (33.4,46.0)	36.3 (30.4,42.3)	35.6 (29.7,41.5)	36.5 (30.6,42.5)	39.8 (33.5,46.0)
	1b	Min	18.1 (13.9,22.4)	19.2 (14.8,23.5)	17.5 (13.4,21.7)	17.2 (13.1,21.3)	17.6 (13.5,21.8)	19.2 (14.9,23.5)
		Max	37.0 (31.0,43.0)	39.1 (32.9,45.3)	35.8 (29.8,41.7)	35.1 (29.2,40.9)	36.0 (30.0,41.9)	39.1 (33.0,45.3)
	2	Min	25.4 (20.4,30.4)	26.8 (21.6,32.0)	24.5 (19.6,29.5)	24.0 (19.2,28.9)	24.7 (19.8,29.6)	26.8 (21.7,31.9)
		Max	**51.8 (44.7,58.9)**	**54.7 (47.3,62.1)**	**50.1 (43.0,57.1)**	**49.0 (42.1,56.0)**	**50.3 (43.3,57.3)**	**54.8 (47.5,62.1)**
	2b	Min	24.5 (19.6,22.4)	25.9 (20.8,23.5)	23.7 (18.8,21.7)	23.2 (18.4,21.3)	23.8 (19.0,21.8)	25.9 (20.9,23.5)
		Max	50.0 (43.0,57.0)	52.8 (45.5,60.0)	48.3 (41.4,55.2)	47.3 (40.5,54.1)	48.6 (41.7,55.4)	52.8 (45.7,60.0)
	3	Min	**15.3 (11.4,19.1)**	**16.1 (12.1,20.1)**	**14.8 (10.9,18.6)**	**14.5 (10.7,18.2)**	**14.8 (11.0,18.6)**	**16.1 (12.2,20.1)**
		Max	31.1 (25.6,36.7)	32.9 (27.2,38.6)	30.1 (24.7,35.6)	29.5 (24.1,34.9)	30.3 (24.8,35.7)	32.9 (27.3,38.6)
**Greece**	1	Min	5.4 (4.0,15.2)	4.3 (3.1,11.7)	4.2 (3.0,14.1)	5.3 (4.0,15.7)	6.2 (4.8,16.7)	6.9 (5.4,17.7)
		Max	21.5 (17.5,25.4)	17.3 (13.7,20.8)	16.1 (12.7,19.6)	21.1 (17.2,25.0)	25.5 (21.2,29.9)	27.4 (22.9,31.9)
	1b	Min	13.1 (11.1,15.2)	9.9 (8.2,11.7)	12.1 (10.1,14.1)	13.6 (11.5,15.7)	14.5 (12.4,16.7)	15.5 (13.2,17.7)
		Max	50.7 (44.6,56.7)	41.5 (36.1,47.0)	46.1 (40.3,51.9)	53.1 (46.8,59.3)	58.0 (51.5,64.5)	56.4 (49.9,62.8)
	2	Min	6.1 (4.7,7.5)	4.8 (3.6,6.1)	4.7 (3.5,6.0)	6.0 (4.6,7.4)	7.0 (5.5,8.5)	7.9 (6.3,9.5)
		Max	24.4 (20.2,28.6)	19.6 (15.8,23.4)	18.3 (14.7,22.0)	24.0 (19.8,28.2)	29.0 (24.4,33.6)	31.1 (26.3,35.9)
	2b	Min	14.8 (12.6,15.2)	11.2 (9.3,11.7)	13.7 (11.6,14.1)	15.4 (13.2,15.7)	16.4 (14.1,16.7)	17.5 (15.1,17.7)
		Max	**57.3 (50.8,63.8)**	**47.1 (41.2,52.9)**	**52.2 (46.0,58.4)**	**60.1 (53.4,66.7)**	**65.6 (58.7,72.5)**	**63.7 (56.9,70.6)**
	3	Min	**4.2 (3.0,5.4)**	**3.3 (2.3,4.4)**	**3.3 (2.3,4.3)**	**4.1 (3.0,5.3)**	**4.8 (3.6,6.1)**	**5.5 (4.1,6.8)**
		Max	16.9 (13.4,20.4)	13.6 (10.4,16.7)	12.7 (9.6,15.7)	16.6 (13.1,20.1)	20.0 (16.2,23.9)	21.5 (17.5,25.5)
**Norway**	1	Min	13.1 (11.7,14.2)	11.0 (9.7,12.1)	11.7 (10.4,12.8)	13.8 (12.4,15.0)	15.3 (13.8,16.5)	13.4 (12.1,14.6)
		Max	33.3 (30.4,36.3)	27.5 (24.8,30.3)	29.9 (27.0,32.8)	35.7 (32.7,38.8)	40.3 (37.0,43.5)	32.7 (30.0,35.5)
	1b	Min	12.9 (11.6,14.2)	10.8 (9.6,12.1)	11.6 (10.3,12.8)	13.6 (12.2,15.0)	15.1 (13.7,16.5)	13.3 (12.0,14.6)
		Max	33.1 (30.2,36.1)	27.4 (24.6,30.1)	29.7 (26.9,32.6)	35.5 (32.4,38.6)	40.0 (36.8,43.3)	32.6 (29.8,35.3)

Country	Method	Estimate	Study period	2014	2015	2016	2017	2018
	2	Min	15.1 (13.7,16.6)	12.7 (11.3,14.0)	13.5 (12.1,14.9)	15.9 (14.4,17.4)	17.6 (16.1,19.2)	15.5 (14.1,16.9)
		Max	**38.5 (35.4,41.7)**	**31.9 (28.9,34.8)**	**34.6 (31.5,37.7)**	**41.3 (38.0,44.7)**	**46.6 (43.1,50.1)**	**37.9 (34.9,40.9)**
	2b	Min	13.6 (12.3,14.2)	11.4 (10.1,12.1)	12.2 (10.8,12.8)	14.3 (12.9,15.0)	15.8 (14.3,16.5)	14.2 (12.8,14.6)
		Max	36.5 (33.5,39.6)	30.2 (27.3,33.1)	32.8 (29.8,35.8)	39.1 (35.9,42.4)	44.1 (40.7,47.5)	36.1 (33.2,39.0)
	3	Min	**12.8 (11.5,14.2)**	**10.8 (9.5,12.0)**	**11.5 (10.2,12.8)**	**13.5 (12.1,14.9)**	**15.0 (13.6,16.4)**	**13.2 (11.9,14.5)**
		Max	32.7 (29.8,35.6)	27.0 (24.3,29.8)	29.4 (26.5,32.2)	35.1 (32.0,38.2)	39.6 (36.3,42.8)	32.1 (29.4,34.9)
**Portugal**	1	Min	6.7 (5.3,10.3)	NA	NA	NA	NA	9.1 (7.4,10.3)
		Max	11.3 (9.2,13.4)	7.0 (5.4,8.6)	9.4 (7.5,11.2)	10.5 (8.6,12.5)	12.1 (10.0,14.2)	19.7 (16.6,22.8)
	1b	Min	8.6 (7.0,10.3)	NA	NA	NA	NA	8.6 (7.0,10.3)
		Max	**33.3 (29.7,36.9)**	**10.6 (8.6,12.6)**	**13.9 (11.6,16.1)**	**14.7 (12.4,17.0)**	**16.5 (14.0,18.9)**	**34.5 (30.3,38.6)**
	2	Min	**6.2 (4.8,7.6)**	NA	NA	NA	NA	8.3 (6.7,10.0)
		Max	10.4 (8.4,12.3)	**6.4 (4.9,8.0)**	**8.6 (6.8,10.3)**	**9.6 (7.8,11.5)**	**11.1 (9.1,13.1)**	18.1 (15.1,21.1)
	2b	Min	7.9 (6.3,10.3)	NA	NA	NA	NA	**7.9 (6.3,10.3)**
		Max	30.9 (27.4,34.3)	9.9 (8.0,11.8)	13.0 (10.8,15.2)	13.8 (11.6,16.1)	15.5 (13.1,17.9)	32.0 (28.0,36.0)
	3	Min	8.4 (6.8,10.0)	NA	NA	NA	NA	11.3 (9.4,13.2)
		Max	14.1 (11.7,16.4)	8.7 (7.0,10.5)	11.7 (9.6,13.7)	13.1 (10.9,15.3)	15.1 (12.7,17.4)	24.5 (21.0,28.0)
**Overall**	1	Min	14.5 (12.5,18.2)	13.4 (11.5,16.8)	14.0 (12.1,17.9)	14.3 (12.4,18.3)	14.9 (12.9,18.9)	15.8 (13.8,19.3)
		Max	35.1 (32.0,38.2)	30.6 (27.8,33.5)	31.9 (29.0,34.8)	35.4 (32.3,38.5)	37.6 (34.4,40.7)	40.0 (36.8,43.3)
	1b	Min	16.1 (14.1,18.2)	14.8 (12.8,16.8)	15.8 (13.8,17.9)	16.2 (14.1,18.3)	16.8 (14.7,18.9)	17.2 (15.0,19.3)
		Max	40.8 (37.5,44.1)	35.2 (32.1,38.2)	37.5 (34.4,40.7)	41.1 (37.8,44.4)	43.3 (39.9,46.7)	46.9 (43.4,50.5)
	2	Min	18.3 (16.1,20.5)	17.3 (15.1,19.5)	17.9 (15.7,20.1)	18.0 (15.8,20.2)	18.5 (16.3,20.8)	19.8 (17.5,22.2)
		Max	41.5 (38.2,44.8)	37.1 (33.9,40.3)	38.4 (35.2,41.6)	41.6 (38.3,44.9)	43.8 (40.4,47.2)	46.6 (43.1,50.2)
		Min	19.2 (17.0,18.2)	17.9 (15.7,16.8)	18.9 (16.7,17.9)	19.2 (16.9,18.3)	19.7 (17.4,18.9)	20.5 (18.1,19.3)

Country	Method	Estimate	Study period	2014	2015	2016	2017	2018
	2b	Max	**46.4 (42.9,50.0)**	**40.8 (37.5,44.1)**	**43.3 (39.9,46.7)**	**46.6 (43.1,50.2)**	**48.9 (45.3,52.5)**	**52.6 (48.9,56.4)**
	3	Min	**14.0 (12.4,15.6)**	**12.8 (11.2,14.3)**	**13.5 (11.9,15.1)**	**13.9 (12.2,15.5)**	**14.5 (12.8,16.1)**	**15.3 (13.6,17.0)**
		Max	34.2 (31.2,37.3)	29.6 (26.7,32.4)	31.1 (28.2,33.9)	34.6 (31.5,37.6)	36.6 (33.5,39.8)	39.3 (36.1,42.6)

Within each method, the table shows the widest variability observed (minimum adjusted, maximum crude). In Portugal, only one of the participating centres reported an extended population, and only for 2018. Therefore, minimum prevalence estimates could only be obtained for that year. Values highlighted in bold are the lowest and highest obtained in the sensitivity analyses (as a whole) per country and time period.

Abbreviations: CI, confidence interval; NA, not available; NE, not estimable; PF-ILDs, progressive-fibrosing interstitial lung diseases.

References

1. European Commission. Eurostat [Internet]. 2019 [cited 2019 Oct 4].Available from: https://ec.europa.eu/eurostat/web/population-demography-migration-projections/data/database.

2. Hyldgaard C, Hilberg O, Muller A, Bendstrup E. A cohort study of interstitial lung diseases in central Denmark. *Respir Med* 2014; 108: 793–799.

3. Statistics Norway. 04859: Area and population of urban settlements (US) 2000 - 2019 [Internet]. [cited 2020 Sep 17].Available from: https://www.ssb.no/en/statbank/table/04859/.

4. Statbel. Structure de la population [Internet]. 2019 [cited 2019 Feb 17].Available from: https://statbel.fgov.be/fr/themes/population/structure-de-la-population.

5. Le Portail des Statistiques Grand-Duché de Luxemburg. Population structure [Internet]. 2020 [cited 2020 Nov 5].Available from: https://statistiques.public.lu/stat/ReportFolders/ReportFolder.aspx?IF_Language=eng&MainTheme=2&FldrName=1.
